# Farnesoid X receptor agonist tropifexor attenuates cholestasis in a randomised trial in patients with primary biliary cholangitis

**DOI:** 10.1016/j.jhepr.2022.100544

**Published:** 2022-07-21

**Authors:** Christoph Schramm, Heiner Wedemeyer, Andrew Mason, Gideon M. Hirschfield, Cynthia Levy, Kris V. Kowdley, Piotr Milkiewicz, Ewa Janczewska, Elena Sergeevna Malova, Johanne Sanni, Phillip Koo, Jin Chen, Subhajit Choudhury, Lloyd B. Klickstein, Michael K. Badman, David Jones

**Affiliations:** 1 Medizinische Klinik und Poliklinik Universitätsklinikum Hamburg Eppendorf, Hamburg, Germany; 2 Martin Zeitz Center for Rare Diseases, University Medical Center Hamburg-Eppendorf, Hamburg, Germany; 3 Hamburg Center of Translational Immunology, Hamburg, Germany; 4 Department of Gastroenterology and Hepatology, Essen University Hospital, Essen, Germany; 5 Division of Gastroenterology, University of Alberta, Edmonton, AB, Canada; 6 Toronto Centre for Liver Disease, Department of Medicine, University of Toronto, Toronto, ON, Canada; 7 University of Miami, Schiff Center for Liver Diseases, Miami, FL, USA; 8 Liver Institute Northwest, Washington State University, Seattle, WA, USA; 9 Liver and Internal Medicine Unit, Medical University of Warsaw, Warsaw, Poland; 10 Translational Medicine Group, Pomeranian Medical University, Szczecin, Poland; 11 ID Clinic, Myslowice Poland; 12 Department of Basic Medical Sciences, School of Health Sciences in Bytom, Medical University of Silesia, Bytom, Poland; 13 Medical Company Hepatolog, LLC, Samara, Russia; 14 Novartis Institutes for Biomedical Research, Basel, Switzerland; 15 Sannity Consulting Ltd, Worthing, UK; 16 Novartis Pharmaceuticals Corporation, East Hanover, NJ, USA; 17 Novartis Healthcare Pvt. Ltd., Hyderabad, India; 18 Novartis Institutes for Biomedical Research, Cambridge, MA, USA; 19 The Newcastle Upon Tyne Hospitals, NHS Foundation Trust, Royal Victoria Infirmary, Newcastle, UK

**Keywords:** Tropifexor, Farnesoid X receptor, Primary biliary cholangitis, Proof of concept, γ-Glutamyl transferase, Pruritus, AE, adverse event, ALP, alkaline phosphatase, ALT, alanine aminotransferase, AUC, area under the concentration–time curve, C4, 7-alpha-hydroxy-4-cholesten-3-one, CL/F,ss, the apparent systemic clearance following oral administration at steady state, C_max_, maximum plasma concentration, FGF19, fibroblast growth factor 19, FXR, farnesoid X receptor, GGT, γ-glutamyl transferase, HDL, high-density lipoprotein, LDL, low-density lipoprotein, NASH, non-alcoholic steatohepatitis, OCA, obeticholic acid, pBAD, primary bile acid diarrhoea, PBC, primary biliary cholangitis, PD, pharmacodynamic, PRO, patient-reported outcome, qd, once daily, QoL, quality of life, R_acc_, accumulation ratio, SAE, serious adverse event, T_max_, time to reach C_max_, ULN, upper limit of normal, VAS, visual analogue scale

## Abstract

**Background & Aims:**

The safety, tolerability, and efficacy of the non-bile acid farnesoid X receptor agonist tropifexor were evaluated in a phase II, double-blind, placebo-controlled study as potential second-line therapy for patients with primary biliary cholangitis (PBC) with an inadequate ursodeoxycholic acid response.

**Methods:**

Patients were randomised (2:1) to receive tropifexor (30, 60, 90, or 150 μg) or matched placebo orally once daily for 28 days, with follow-up on Days 56 and 84. Primary endpoints were safety and tolerability of tropifexor and reduction in levels of γ-glutamyl transferase (GGT) and other liver biomarkers. Other objectives included patient-reported outcome measures using the PBC-40 quality-of-life (QoL) and visual analogue scale scores and tropifexor pharmacokinetics.

**Results:**

Of 61 enrolled patients, 11, 9, 12, and 8 received 30-, 60-, 90-, and 150-μg tropifexor, respectively, and 21 received placebo; 3 patients discontinued treatment because of adverse events (AEs) in the 150-μg tropifexor group. Pruritus was the most frequent AE in the study (52.5% [tropifexor] *vs*. 28.6% [placebo]), with most events of mild to moderate severity. Decreases seen in LDL-, HDL-, and total-cholesterol levels at 60-, 90-, and 150 μg doses stabilised after treatment discontinuation. By Day 28, tropifexor caused 26–72% reduction in GGT from baseline at 30- to 150-μg doses (*p* <0.001 at 60-, 90-, and 150-μg tropifexor *vs*. placebo). Day 28 QoL scores were comparable between the placebo and tropifexor groups. A dose-dependent increase in plasma tropifexor concentration was observed, with 5- to 5.55-fold increases in AUC_0-8h_ and C_max_ between 30- and 150-μg doses.

**Conclusions:**

Tropifexor showed improvement in cholestatic markers relative to placebo, predictable pharmacokinetics, and an acceptable safety–tolerability profile, thereby supporting its potential further clinical development for PBC.

**Lay summary:**

The bile acid ursodeoxycholic acid (UDCA) is the standard-of-care therapy for primary biliary cholangitis (PBC), but approximately 40% of patients have an inadequate response to this therapy. Tropifexor is a highly potent non-bile acid agonist of the farnesoid X receptor that is under clinical development for various chronic liver diseases. In the current study, in patients with an inadequate response to UDCA, tropifexor was found to be safe and well tolerated, with improved levels of markers of bile duct injury at very low (microgram) doses. Itch of mild to moderate severity was observed in all groups including placebo but was more frequent at the highest tropifexor dose.

**Clinical Trials Registration:**

This study is registered at ClinicalTrials.gov (NCT02516605).

## Highlights

• Phase II study on the farnesoid X receptor agonist, tropifexor, in patients with primary biliary cholangitis.
• Itch of mild to moderate severity was the most frequent adverse event.
• Markers of bile duct injury were reduced with tropifexor after 4 weeks of therapy.
• Higher tropifexor exposure was observed in patients with PBC than that in healthy volunteers.

## Graphical abstract

**Figure:**
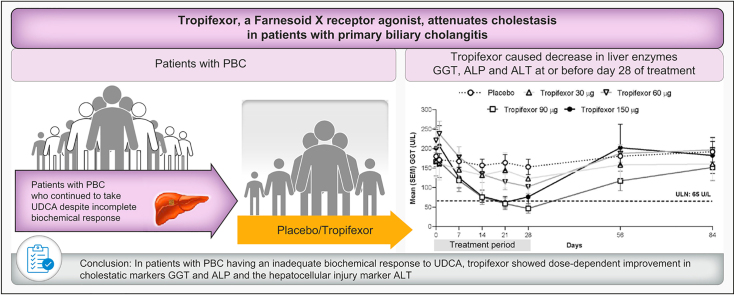

## Introduction

Primary biliary cholangitis (PBC) is a chronic, cholestatic liver disease characterised by damage to, and destruction of, biliary epithelial cells and the intrahepatic bile ducts that they line, leading to cholestasis and subsequent progression to biliary fibrosis and cirrhosis.^1^ PBC predominantly affects women aged >40 years and has an overall prevalence of 1.91–40.2 per 100,000 people.[1], [2], [3], [4] Pruritus, fatigue, and upper abdominal discomfort are the most frequent symptoms of PBC, with a negative effect on the quality of life (QoL) of patients.^2^^,^^5^

Ursodeoxycholic acid (UDCA) as first-line, standard-of-care therapy for management of PBC has shown to extend transplant-free survival[6], [7], [8]; nevertheless, 25–50% of patients exhibit an inadequate biochemical response to UDCA,^1^ which substantially increases the risk of death or need for liver transplantation.^8^^,^^9^

The farnesoid X receptor (FXR) has long been a target of interest for intrahepatic cholestasis.^10^^,^^11^ The bile acid FXR agonist obeticholic acid (OCA) has been clinically validated in multiple phase II and III studies[12], [13], [14] and has been approved in combination with UDCA for patients with PBC having an inadequate response to UDCA or as monotherapy in patients intolerant of UDCA.^15^ However, a high incidence of pruritus and impact of persistent decrease in HDL cholesterol on long-term cardiovascular risk remain areas of uncertainty with OCA.^12^^,^^13^

Tropifexor (LJN452) is a non-bile acid FXR agonist with sub-nanomolar potency attributed to a unique bicyclic nortropine-substituted benzothiazole carboxylic acid moiety that has been optimised for enhanced fit within the ligand-binding domain of FXR.^16^ In rodents, tropifexor potently regulated FXR target genes in the liver and intestine and showed superior efficacy to OCA in models of non-alcoholic steatohepatitis (NASH)^16^^,^^17^ and cholestasis.^18^ Tropifexor has a pharmacokinetic profile suitable for once daily (qd) dosing in humans^19^ and has shown effective FXR target engagement via transient and dose-dependent increases in fibroblast growth factor 19 (FGF19) in healthy volunteers^19^ and patients with NASH^20^^,^^21^ and primary bile acid diarrhoea (pBAD).^22^

The purpose of this study was to evaluate the safety, tolerability, efficacy, pharmacokinetics, and pharmacodynamics of multiple doses of tropifexor in patients with PBC with an inadequate response to UDCA. This study was originally designed in 2 parts. However, the study was terminated early, and part 2 was not executed because data revealed that part 1 fulfilled the strategic purpose of the study. We report the results of part 1 of the study here.

## Patients and methods

### Patient population

Patients aged ≥18 years with BMI of 18–40 kg/m^2^, a confirmed diagnosis of PBC (presence of ≥2 of 3 diagnostic criteria), and presence of ≥1 marker of disease severity and taking UDCA for ≥12 or ≥6 months and having reached a plateau in alkaline phosphatase (ALP) response, with no changes in dose for ≥3 months before Day 1, were included in the study. All patients provided written informed consent. Detailed inclusion and exclusion criteria are described in Table S1.

### Study design and treatments

This was a randomised, multicentre, double-blind, placebo-controlled study (NCT02516605) to assess the safety, tolerability, and efficacy of multiple, escalating doses of tropifexor in patients with PBC who had an incomplete biochemical response to, but continued to take, UDCA. The study was conducted between September 2015 and August 2018 at 23 centres in 6 countries (Canada, Germany, Poland, Russia, the United Kingdom, and the United States). The study protocol was approved by the respective institutional review boards and conformed to the ethical guidelines of the Declaration of Helsinki. Four cohorts of ∼15 patients each were enrolled and randomised in a 2:1 ratio to receive tropifexor (30, 60, 90, or 150 μg) qd or matched placebo (Fig. S1). Each patient underwent a screening visit, baseline assessments, a 28-day treatment period, and follow-up visits on Days 56 and 84. Dose-escalation review and interim analysis were performed when ≥12 patients completed dosing and Day 28 assessments in a specific cohort. The next higher dose was initiated once the previous dose-escalation review was complete.

### Randomisation and blinding

All eligible patients were assigned randomisation numbers. To ensure that the treatment assignment was unbiased and concealed from the participants and investigator staff, the randomisation list was generated using a validated system that automated the random assignment of treatment arms to randomization numbers in the specified ratio.

In this double-blind study, participants, investigator staff (except for the pharmacy staff or authorised designee responsible for dispensing study medication), persons performing the assessments, and data analysts remained blinded to the identity of study treatments. The identity of the treatments was concealed using study drugs that were all identical in packaging, labelling, schedule of administration, appearance, and odour. The sponsor was unblinded, and the randomisation was released to the clinical trial team including the modeler when the interim analysis was performed after participants in a cohort had completed Day 28 of the study.

### Study objectives and endpoints

The primary objectives were to evaluate (1) the safety and tolerability of tropifexor as assessed using adverse events (AEs) and serious AEs (SAEs) and (2) the effect of tropifexor on cholestatic markers. Secondary objectives included the evaluation of (1) tropifexor pharmacokinetics and (2) patient-reported outcome (PRO) assessment by change in PBC-40 QoL tool scores and 100-mm visual analogue scale (VAS) itch scores. Levels of FXR engagement markers, namely, FGF19 and 7-alpha-hydroxy-4-cholesten-3-one (C4), were evaluated as exploratory objectives. Total, LDL, and HDL cholesterol levels were assessed to determine the effect of tropifexor on systemic lipids. Although ALP is the recognised surrogate marker for response to therapy, a primary endpoint of GGT reduction was chosen to avoid a potential confounding effect of tropifexor on serum ALP levels via ALP gene induction resulting from FXR activation.^18^^,^^23^ The global PBC study group data support the use of GGT as a prognostic marker in PBC.^24^

### Assessments

Levels of liver biomarkers GGT, ALP, alanine aminotransferase (ALT), and total bilirubin and those of plasma lipids were assessed at baseline and Days 1, 7, 14, 21, 28, 56, and 84. The PBC-40 questionnaire^25^ was used to estimate patient-reported QoL scores at baseline and Days 28, 56, and 84. The measure consisted of 40 questions, grouped into 6 domains (itch, symptoms, fatigue, cognition, emotional well-being, and social/family well-being), each scored on a scale of 1 (least impact) to 5 (greatest impact); the higher the scores, the poorer the QoL. Itch severity was assessed at baseline and Days 7, 14, 21, 28, 56, and 84 using global VAS^26^ (score ranges: 0 [none at all] to 10 [worst imaginable itch]).

Pharmacokinetic samples were collected at predose, 1, 2, 4, 6 (optional), and 8 (optional) h on Day 1 and at predose, 1, 2, 3, 4, 6, 8 (optional), and 24 (optional) h on Day 28. Samples were also collected on Days 7, 14, and 21 predose and on Day 56. Plasma tropifexor levels were determined using a validated liquid chromatography mass spectrometry method.^19^ The lower limit of quantification was ≤20 pg/ml. The primary pharmacokinetic assessments included area under the concentration–time curve from time 0 to *t* (AUC_0-t_), where *t* was a defined time point after administration; maximum plasma concentration (C_max_); time to C_max_ (T_max_); the apparent systemic clearance from plasma at steady state following oral administration (CL/F,ss); and accumulation ratio (R_acc_).

Serum samples for pharmacodynamic biomarkers FGF19 and C4 were collected on Day 1 predose; at 1, 2, 4, 6, and 8 h on Days 7, 14, and 21 predose; on Day 28 predose; and at 1, 2, 4, 6, 8, and 24 h on Day 56.

Safety assessments included evaluation of all AEs, with their severity and relationship to study drug, and SAEs.

### Statistical analysis

Details of sample size calculation and statistical analysis methods are described in Table S2. All analyses were performed using pooled placebo across all cohorts as control, and no multiplicity adjustments were applied. For the primary endpoint assessment, serum GGT values at all time points were logarithmically transformed, and change from baseline was calculated as the difference between each log-transformed post-dose serum GGT value and log-transformed baseline serum GGT value and converted to percent change from baseline. Log-transformed ratio to baseline was analysed using repeated-measures analysis of covariance.

## Results

### Patient characteristics

Out of 61 enrolled patients, 40 were randomised to receive tropifexor and 21 comprised the pooled placebo group (Fig. S2). In all, 11, 9, 12, and 8 patients received 30-, 60-, 90-, and 150-μg tropifexor, respectively. In cohort 4, a total of 3 patients were discontinued from treatment because of AEs and 1 patient because of subject decision. One patient randomised to receive placebo in cohort 2 was discontinued because of protocol deviation.

Demographics and baseline characteristics were comparable across all groups. Most patients (96.7%) were female, and the predominant race was Caucasian (93.4%; Table 1). More than half the patients (55.7%) were on UDCA for >5 years. Liver biomarker levels and PBC-40 scores at baseline were comparable between groups.

**Table 1:** **Demographics and baseline characteristics (safety analysis set)**.

Parameter		Placebo	Tropifexor			
			30 μg	60 μg	90 μg	150 μg
		**n** = **21**	**n** = **11**	**n** = **9**	**n** = **12**	**n** = **8**
Age (years), mean (SD)		53.7 (10.19)	58.6 (12.42)	57.9 (11.21)	53.6 (7.42)	57.4 (13.81)
Female sex, n (%)		21 (100.0)	11 (100.0)	7 (77.8)	12 (100.0)	8 (100.0)
Race, n (%)						
Caucasian		19 (90.5)	10 (90.9)	8 (88.9)	12 (100.0)	8 (100.0)
Asian/other		2 (9.5)	1 (9.1)	1 (11.1)	–	–
BMI (kg/m^2^), mean (SD)		27.3 (5.70)	26.4 (4.08)	26.7 (4.91)	29.1 (7.00)	26.2 (4.17)
Total daily UDCA dose (mg/kg), mean (SD)		14.9 (2.64) n = 20∗	15.2 (3.21)	13.7 (3.35)	14.2 (4.55) n = 11∗	16.8 (5.35) n = 7∗
History of UDCA use, n (%)						
>5 years		10 (47.6)	7 (63.6)	6 (66.7)	7 (58.3)	4 (50.0)
>3 to ≤5 years		2 (9.5)	2 (18.2)	1 (11.1)	3 (25.0)	—
>6 months to ≤3 years		8 (38.1)	2 (18.2)	2 (22.2)	2 (16.7)	4 (50.0)
Liver function tests, mean (SD)∗	**Normal range**	**n** = **19**	**n** = **10**	**n** = **9**	**n** = **11**	**n** = **8**
Alkaline phosphatase (U/L)	35–104	338 (122.1)	316 (173.6)	303 (96.0)	306 (92.0)	284 (109.4)
γ-glutamyl transferase (U/L)	2–65	171 (69.2)	162 (137.1)	238 (97.9)	167 (136.2)	207 (147.6)
Alanine aminotransferase (U/L)	0–45	54.2 (22.22)	55.2 (42.19)	52.3 (19.75)	51.5 (22.90)	54.6 (28.80)
Aspartate aminotransferase (U/L)	0–41	48.9 (8.75) n = 16	58.6 (39.12)	57.3 (19.24)	45.6 (16.98)	47.3 (20.67)
Total bilirubin (μmol/L)	2–21	11.4 (6.64)	8.2 (4.66)	11.4 (8.06)	8.6 (3.32)	10.4 (4.07)
Total PBC-40 QoL score, mean (SD)†		**n** = **18**	**n** = **9**	**n** = **9**	**n** = **12**	**n** = **8**
		105.5 (38.33)	96.1 (31.68)	91.8 (38.96)	88.3 (26.50)	94.8 (42.23)
VAS itch score (mm), median (min, max)†		**n** = **20**	**n** = **9**	**n** = **9**	**n** = **12**	**n** = **8**
		25.0 (0.0, 90.0)	33.0 (15.0, 67.0)	35.0 (6.0, 70.0)	5.0 (0.0, 88.0)	24.5 (1.0, 86.0)
VAS itch score ≥40 mm, n (%)		10 (50.0)	4 (44.4)	4 (44.4)	2 (16.7)	3 (37.5)

QoL, quality of life; UDCA, ursodeoxycholic acid; VAS, visual analogue scale.

∗ One patient was not taking UDCA in the placebo group and data on exact total daily UDCA dose was not available for 1 patient each in the tropifexor 90 and 150 μg groups.

† Based on pharmacodynamics analysis set.

### Safety and tolerability of tropifexor

AEs were mostly of grade 1 severity (73%). Pruritus was the most frequent AE in all groups (Table 2) with 2 patients in placebo and 2 in each tropifexor dose group experiencing grade 1 pruritus. Grade 2 pruritus was experienced by 3 patients in the placebo group and by 1, 4, 3, and 2 patients receiving 30-, 60-, 90-, and 150-μg tropifexor, respectively. One patient in the placebo group and 3 in the 150-μg tropifexor group experienced grade 3 pruritus. No deaths or SAEs were reported. AEs leading to treatment discontinuation were observed in 3 patients in the 150-μg tropifexor group. One patient was discontinued from treatment after 12 days owing to elevated ALT; 1 after 11 days owing to pruritus, insomnia, and trace proteinuria (present before dosing); and 1 after 8 days owing to pruritus.

**Table 2:** **Adverse events (safety analysis set)**.

Parameter, n (%)	Placebo	Tropifexor			
		30 μg	60 μg	90 μg	150 μg
	n = 21	n = 11	n = 9	n = 12	n = 8
At least 1 AE	16 (76.2)	9 (81.8)	8 (88.9)	11 (91.7)	8 (100)

Incidence ≥15% in any group					
Pruritus	6 (28.6)	3 (27.3)	6 (66.7)	5 (41.7)	7 (87.5)
Grade 1	2 (9.5)	2 (18.2)	2 (22.2)	2 (16.7)	2 (25.0)
Grade 2	3 (14.3)	1 (9.1)	4 (44.4)	3 (25.0)	2 (25.0)
Grade 3	1 (4.8)	–	–	–	3 (37.5)
Nausea	3 (14.3)	1 (9.1)	1 (11.1)	2 (16.7)	0 (0.0)
Headache	3 (14.3)	–	–	2 (16.7)	1 (12.5)
Dyspepsia	–	1 (9.1)	2 (22.2)	–	1 (12.5)
Nasopharyngitis	1 (4.8)	2 (18.2)	–	1 (8.3)	–
Lower abdominal pain	–	2 (18.2)	–	–	–
Increased ALT	–	–	–	–	2 (25.0)
Arthropod bite	–	2 (18.2)	–	–	–
Muscle spasms	–	2 (18.2)	–	–	–
Urinary tract infection	–	–	–	2 (16.7)	–

Incidence of SAEs	0 (0.0)	0 (0.0)	0 (0.0)	0 (0.0)	0 (0.0)
Incidence of study drug-related AEs	9 (42.9)	4 (36.4)	6 (66.7)	8 (66.7)	8 (100.0)
Incidence of study drug-related AEs leading to treatment discontinuation	–	–	–	–	3 (37.5)∗

AE, adverse event; ALT, alanine aminotransferase; AST, aspartate aminotransferase; SAE, serious adverse event.

∗ One patient owing to both insomnia and proteinuria; 1, increased AST; 2, pruritus.

**Fig. 1:**
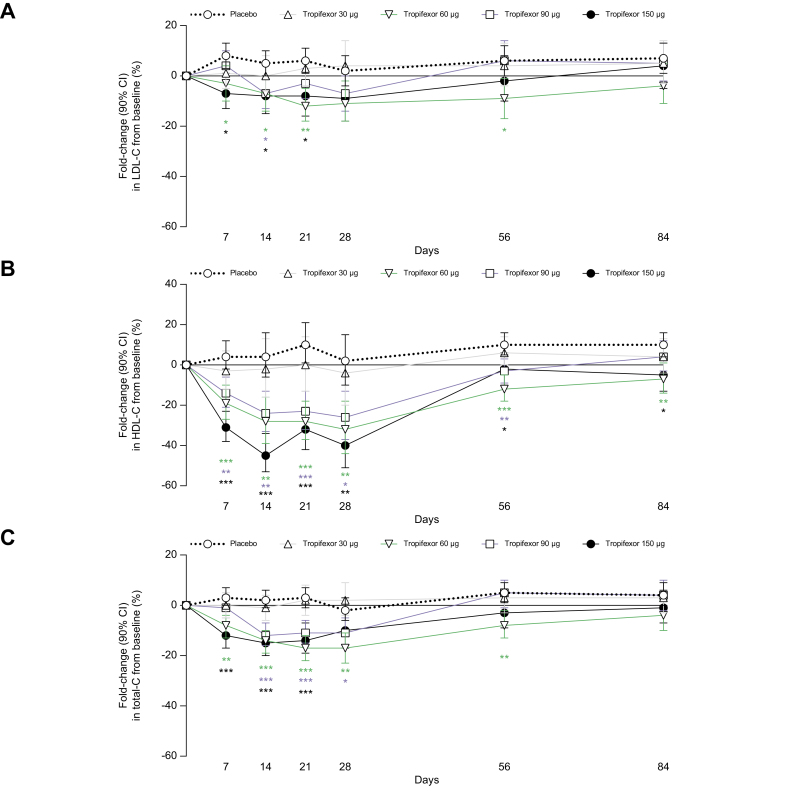
Changes in cholesterol levels. Percent fold-change (90% CI) from baseline to Day 84 in (A) LDL-C, (B) HDL-C, and (C) total-C following once daily administration of placebo or tropifexor at doses 30, 60, 90, and 150 μg. ∗*p* <0.05, ∗∗*p* <0.01, and ∗∗∗*p* <0.001 compared with placebo. HDL-C, HDL cholesterol; LDL-C, LDL cholesterol; total-C, total cholesterol.

A significant decrease in LDL cholesterol from baseline was observed with tropifexor 60, 90 and 150 μg relative to placebo on Days 7, 14, and 21, after which levels restored to baseline values (Fig. 1A). A dose-dependent and significant decrease in HDL cholesterol was also observed with tropifexor 60-, 90-, and 150-μg doses relative to placebo from Days 7 to 84 (Fig. 1B). These resulted in a significant decrease in total cholesterol from baseline relative to placebo in the tropifexor 60, 90, and 150 μg groups (Fig. 1C).

### Effect of tropifexor on liver biomarkers

**Fig. 2:**
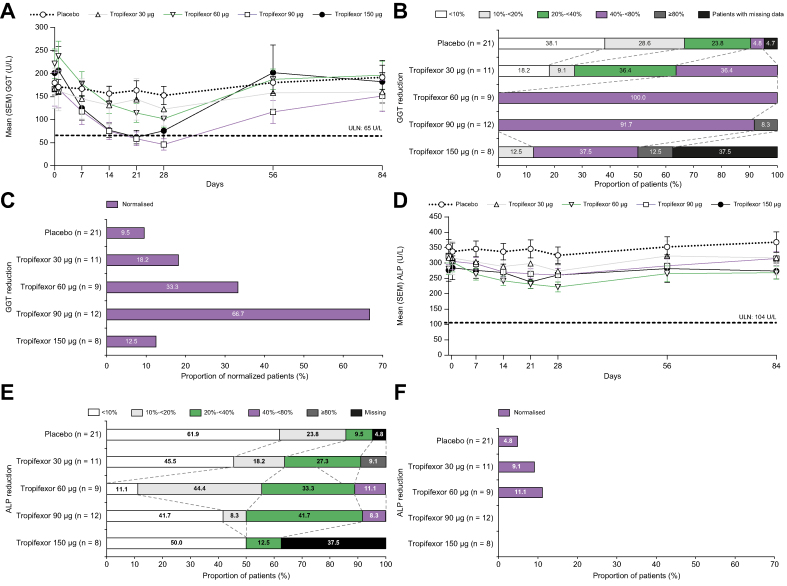

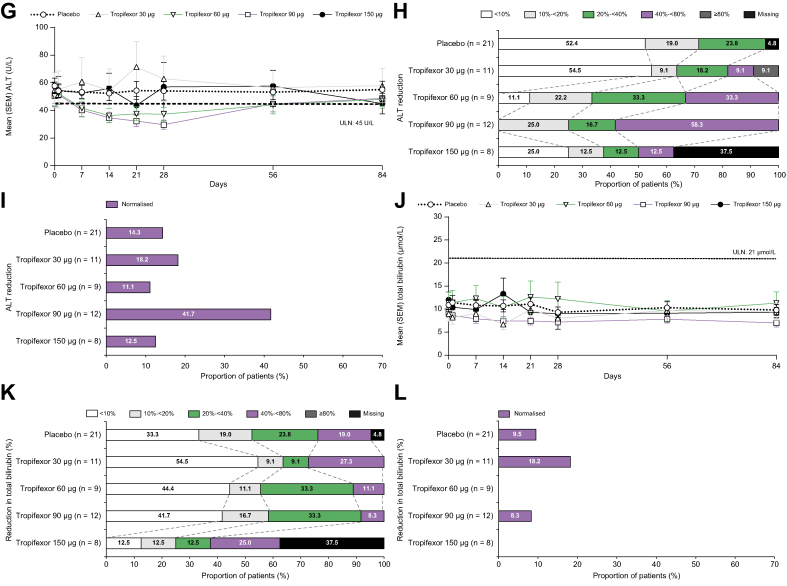
Changes in liver biomarkers. Mean (SEM) levels from baseline to Day 84 and percent reduction on Day 28 in (A–C) GGT, (D–F) ALP, (G-I) ALT, and (J–L) total bilirubin. Patients who achieved GGT <65 U/L, ALP <1.67× ULN (<174 U/L), ALT <45 U/L, or total bilirubin <0.6× ULN (12.6 μmol/L) by Day 28 were considered to have normalised response. ALP, alkaline phosphatase; ALT, alanine aminotransferase; GGT, γ-glutamyl transferase; ULN, upper limit of normal.

Tropifexor caused decreases in liver enzymes GGT, ALP, and ALT at or before Day 28 of treatment (Fig. 2). Mean GGT levels fell below the upper limit of normal (ULN; 65 U/L) in both the 90- and 150-μg tropifexor cohorts by Day 21 (Fig. 2A). Changes were brisk, with a significant decrease in GGT on Day 7 relative to baseline in patients receiving all dose levels of tropifexor. Compared with placebo, after 28 days of treatment, the fold decrease in GGT was significant at 60-, 90-, and 150-μg tropifexor doses (Fig. S3A; *p* <0.001). Most patients in the 60-, 90-, and 150-μg tropifexor groups showed 40 to <80% reduction from baseline GGT levels (Fig. 2B), whereas GGT levels were normalised by Day 28 in 18.2, 33.3, 66.7, and 12.5% of patients in the 30-, 60-, 90-, and 150-μg tropifexor groups, respectively (Fig. 2C).

Mean ALP levels were reduced in the 60- and 90-μg tropifexor groups but remained above the ULN (104 U/L) throughout the study in all treatment groups (Fig. 2D). This reduction reached statistical significance (*p* = 0.001) in comparison with placebo at Day 28 in the 60-μg tropifexor group (Fig. S3B). A dose-dependent 20 to <40% reduction from baseline ALP was observed in 27.3, 33.3, and 41.7% of patients receiving 30-, 60-, and 90-μg tropifexor, respectively (Fig. 2E); however, very few patients achieved reduction of ALP to levels <1.67× ULN on Day 28 (Fig. 2F).

Mean ALT levels were reduced below the ULN (45 U/L) in the 60- and 90-μg tropifexor groups from Days 7 to 28. After tropifexor was discontinued on Day 28, ALT levels rose and steadied to around the ULN by Day 84 (Fig. 2G). A dose-dependent 40−80% decrease from baseline ALT was seen in 9.1, 33.3, 58.3, and 12.5% patients receiving 30-, 60-, 90-, and 150-μg tropifexor (Fig. 2H). Only 18.2, 11.1, 41.7, and 12.5% of patients in the 30-, 60-, 90-, and 150-μg tropifexor groups achieved normalisation (<45 U/L) of ALT response by Day 28, respectively (Fig. 2I). Dose-dependent decreases in ALT levels were observed only for the 60- and 90-μg tropifexor groups on Day 28 (Fig. S3C).

The mean total bilirubin levels were below the ULN (21 μmol/L) in all groups including placebo throughout the study (Fig. 2J), without any significant changes from baseline relative to placebo (Fig. S3D). A reduction of more than 80% in total bilirubin was seen in 2 (25%) patients receiving 150-μg tropifexor (Fig. 2K). Total bilirubin levels were normalised to levels <0.6× ULN in 18.2 and 8.3% of patients receiving 30- and 90-μg tropifexor, respectively (Fig. 2L).

### Effect of tropifexor on PROs

**Fig. 3:**
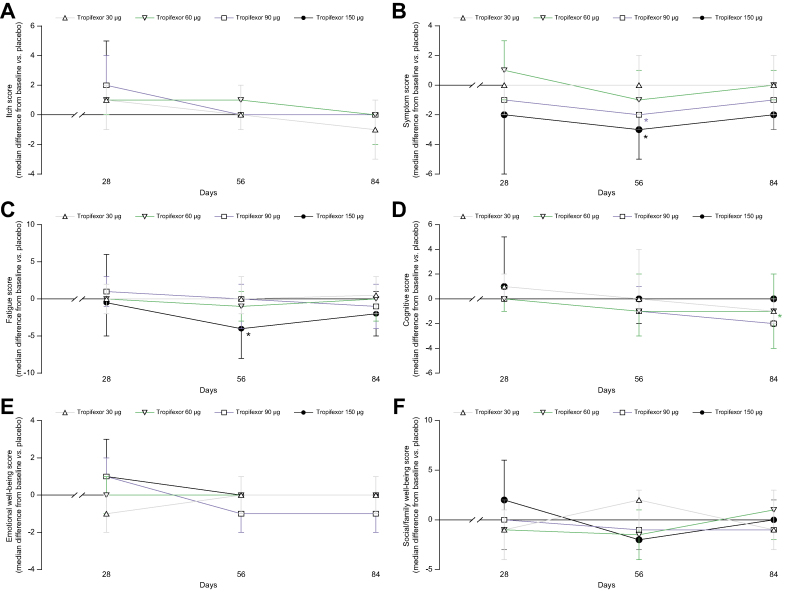
Changes in PBC-40 QoL scores (PD analysis set). Median (90% CI) change from baseline in (A) itch, (B) symptom, (C) fatigue, (D) cognitive, (E) emotional well-being, and (F) social/family well-being scores on Days 28, 56, and 84. ∗*p* <0.05 compared with placebo. PD, pharmacodynamic; QoL, quality of life.

The PBC-40 questionnaire was used to evaluate PROs on QoL and specifically on itch. Although a non-significant increase in median itch scores was observed for all tropifexor groups relative to placebo during the treatment period (Fig. 3A), median symptoms domain scores decreased in the 90- and 150-μg tropifexor groups on Day 28 (*p* >0.05) and after treatment completion (Day 56: 90 μg, *p* = 0.028, and 150 μg, *p* = 0.01; Fig. 3B). Median fatigue scores also significantly improved in the 150-μg tropifexor group by Day 56 (*p* = 0.036; Fig. 3C); however only 4 patients received all 28 doses of study drug. No meaningful changes were observed in the median scores of other domains during treatment (Fig. 3D–F).

**Fig. 4:**
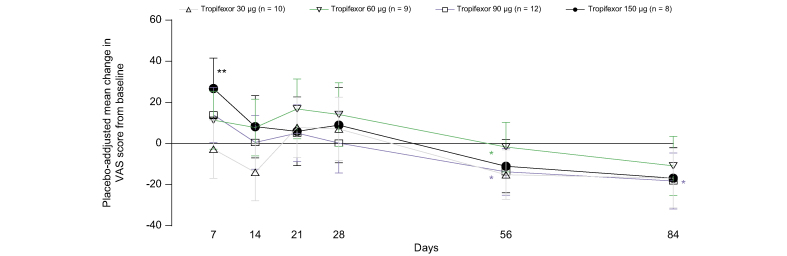
Changes in VAS scores (PD analysis set). Adjusted mean change (90% CI) from baseline in VAS itch scores on Days 7, 14, 21, 28, and 56. ∗*p* <0.05 and ∗∗*p* <0.01 compared with placebo. PD, pharmacodynamic; VAS, visual analogue scale.

On the VAS itch scale, a trend toward increase in mean VAS scores in the 60-, 90-, and 150-μg tropifexor groups relative to placebo was observed on Day 7, which reached statistical significance (*p* = 0.004) only in the 150-μg tropifexor group. After treatment completion, there were significant decreases in these scores for the 30-μg (*p* = 0.039) and 90-μg (*p* = 0.047) tropifexor groups on Day 56 and the 90-μg tropifexor group on Day 84 (*p* = 0.029; Fig. 4).

### Pharmacokinetics and pharmacodynamics of tropifexor

**Fig. 5:**
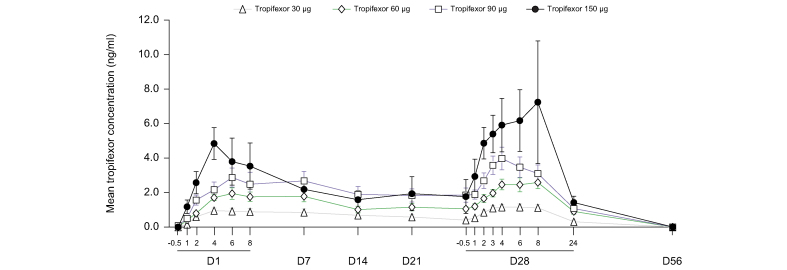
Pharmacokinetic profile of tropifexor. Plasma concentration–time profile of tropifexor following once daily oral administration of tropifexor at doses 30, 60, 90, and 150 μg. Data are represented as mean (SEM). D, day.

A dose-dependent increase in tropifexor plasma concentration was observed following qd oral administration of 30-, 60-, 90-, and 150-μg doses, with a median T_max_ of 4–5 h (range: 0–8 h) post dose (Fig. 5). A 5-fold increase in dose from 30 to 150 μg led to an increase of 5- and 5.55-fold in C_max_ and AUC_0-8h_, respectively; thus, an approximately dose-proportional exposure was demonstrated over this dose range (Table S3). The inter-subject variability ranged from 32.5 to 65.5% for C_max_ and from 22.3 to 64.9% for AUC_0-8h_ across all dose groups. Steady state was reached before Day 14 with an accumulation less than 2-fold.

**Fig 6:**
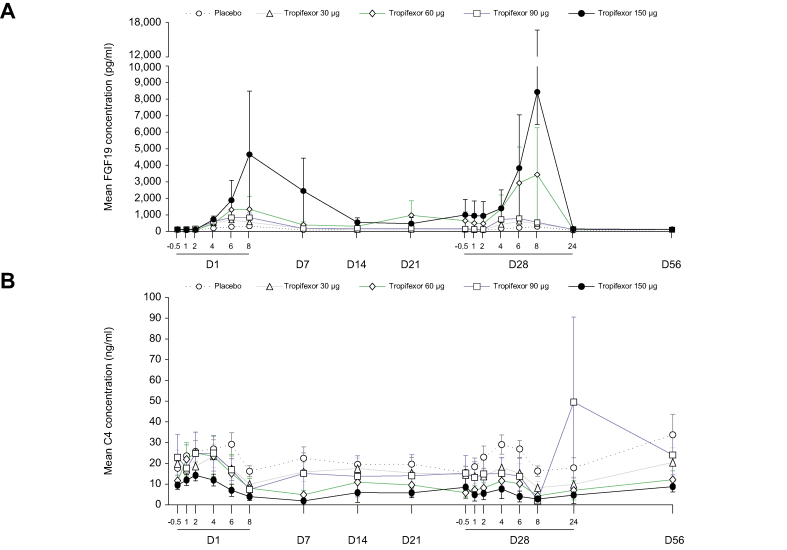
Pharmacodynamic effect of tropifexor. Concentration–time profile of (A) FGF19 and (B) C4 following single ascending doses of tropifexor. Data are represented as mean (SEM). C4, 7-alpha-hydroxy-4-cholesten-3-one; D, day; FGF19, fibroblast growth factor 19.

The pharmacodynamic effects of tropifexor were evaluated by assessing plasma levels of FGF19 and C4 (Fig. 6). FGF19 C_max_ levels were significantly increased relative to placebo on Day 1 (4 h) in all tropifexor-treated groups and on Day 28 in the tropifexor 30 μg (median difference *vs*. placebo [90% CI] pg∗h/ml: 285.1 [160.00–501.50]), 60-μg (797.0 [253.00–1143.30]), and 90-μg tropifexor groups (583.3 [402.00–745.00]). C4 exposure decreased with increasing doses of tropifexor on Days 1 and 28, but these decreases were not significant relative to placebo on Day 28 (*p* >0.05; Table S4).

## Discussion

This was the first study to evaluate the safety, efficacy, pharmacokinetics, and pharmacodynamics of a highly potent, non-bile acid FXR agonist, tropifexor, in patients with PBC who had an inadequate response to UDCA.^16^^,^^17^^,^[19], [20], [21]^,^^27^

The efficacy of tropifexor was confirmed by dose-dependent and significant reductions in cholestatic marker GGT at 4 weeks. Although tropifexor caused reduction in mean GGT levels at all doses tested and normalisation of GGT in a substantial proportion of patients receiving up to 90-μg dose, reduction in ALP was observed only at 60- and 90-μg doses with very few patients achieving normalisation of ALP response. These findings indicate that induction of ALP gene transcription via FXR activation by tropifexor could confound the downstream effect of ALP reduction at higher doses or that treatment beyond 28 days may be required for normalisation of ALP response by tropifexor. Chronic elevation in ALP is one of the key diagnostic criteria for PBC,^1^^,^^3^^,^^4^^,^^28^ and ALP being a strong predictor of long-term outcomes has gained acceptance as a surrogate endpoint and treatment goal for clinical trials in PBC.^29^ Hence, confirmatory studies assessing the efficacy of novel treatments for inadequate UDCA responders have focused on ALP and biomarkers other than GGT as the primary endpoint.^12^^,^^13^^,^^30^ Nonetheless, recent findings from the Global PBC Study Group comprising 14 European and North American centres show that among patients with ALP <1.5× ULN, the risk of liver transplantation or liver-related death is significantly higher when serum GGT levels are >3.2× ULN *vs*. <3.2× ULN, thereby supporting the potential use of GGT as a surrogate clinical endpoint and a primary endpoint in our study.^24^

Tropifexor caused moderate reductions in ALT over the 28-day treatment period; mean ALT levels reduced to the normal range in the 60- and 90-μg tropifexor groups. These modest responses are consistent with those observed by Hirschfield *et al.*^12^ for OCA over 3 months of treatment.

No significant decreases in total bilirubin levels were observed during the 28-day treatment period in our study, most likely because patients with advanced disease were excluded; hence, the majority of patients started and remained below the 0.6× ULN level from baseline through 28 days.

The overall safety and tolerability profile of tropifexor in this study was consistent with that observed in healthy volunteers^19^ and in patients with NASH^20^^,^^21^^,^^27^ and pBAD.^22^ In this study, tropifexor was well tolerated at daily doses of 30, 60, and 90 μg. Although significant decreases in LDL, HDL, and total cholesterol were observed during the 28-day treatment period with 60-, 90-, and 150-μg tropifexor doses, the lipid levels returned to baseline upon treatment discontinuation. Another, less potent, non-bile acid FXR agonist, cilofexor, showed a significant decrease in HDL cholesterol, but not in LDL cholesterol, over 12 weeks of treatment in patients with primary sclerosing cholangitis,^31^ and the bile acid FXR agonist OCA showed dose-dependent decreases in total and HDL cholesterol, but not in LDL cholesterol, over 12 weeks to 12 months of treatment.^12^^,^^13^ Unlike in patients with PBC, tropifexor doses up to 200 μg over 12 weeks caused a dose-dependent and transient increase in LDL cholesterol and decrease in HDL cholesterol in patients with NASH^20^ and moderate increases in total and LDL cholesterol levels relative to placebo without any significant reduction in HDL cholesterol up to 2 weeks of treatment with 60-μg dose in patients with pBAD.^22^ Thus, changes in cholesterol levels in response to FXR agonism with tropifexor vary with the underlying disease.

The most commonly reported AE in the study was pruritus, with the highest incidence reported in the 150-μg tropifexor group. The majority of these events were of severity grade 1 or 2 and led to treatment discontinuation only with the highest (150 μg) dose. To further understand the impact of itch, PBC-40 questionnaire and global VAS scores were used to assess the severity and impact of pruritus on QoL as PROs. Indeed, increases in mean VAS itch score and median PBC-40 itch domain score were noted at all tropifexor doses. Although the changes in itch scores at low doses were modest, a number of factors make the increased reporting of itch in the 150-μg tropifexor group difficult to interpret: (1) the sequential ascending dose cohort design could have introduced reporting bias; (2) approval of OCA during the study could have caused patients with less itch who were prescribed OCA to be excluded from later cohorts of the study; and (3) initiation of recruitment in countries with no access to common antipruritic therapies in this cohort.

Pruritus has not been reported with tropifexor in healthy volunteers,^19^ but dose-dependent increase in pruritus has been observed with OCA in patients with PBC having an inadequate response to UDCA.^12^ In this regard, it is important to note that no improvement in PBC-40 itch domain scores over 12 months of treatment and significant exacerbation, instead of improvement, of VAS itch scores in the first 3 months of OCA treatment were observed in the pivotal PBC OCA International Study of Efficacy study.^13^ These findings indicate that occurrence of pruritus with FXR agonists in NASH^20^^,^^21^^,^^27^ and PBC^12^^,^^13^ could be related to the pathophysiology of these conditions. Unlike OCA, however, tropifexor did not cause clinically relevant pruritus at doses that elicited a liver biomarker response (*i.e.* <150 μg). Because tropifexor is a non-bile acid agonist with lack of activity on TGR5,^16^ a receptor mediating bile acid-induced itch in cholestatic diseases,^32^ further mechanistic studies to elucidate the role of FXR agonists on pruritus including autotaxin or lysophosphatidic acid or Mas-related G protein-coupled receptor X4-mediated pruritus may be warranted.^33^^,^^34^

Other QoL indicators assessed using the PBC-40 questionnaire such as symptom and fatigue domains showed some improvement relative to placebo with the 90- and/or 150-μg tropifexor doses after treatment completion, that is, on Day 56. Given that the scores at Day 56 include a period of sustained effect of tropifexor on biomarker activity, studies with longer treatment duration will be required to understand the long-term effect of therapy on fatigue score response.

Exposure of tropifexor with 28 days of daily dosing was dose-proportional for the 30- to 150-μg range and appeared with greater increases in both C_max_ and AUC and decrease in CL/F,ss than that previously observed in healthy volunteers over 14 days of daily dosing.^19^ The increase in exposure could be caused by either an increase in bioavailability or a decrease in clearance, or both. It is possible that absorption is increased, which could have resulted from an altered bile acid pool in these patients who had been receiving long-term treatment with UDCA.^35^ Given that tropifexor is mainly eliminated via biliary excretion, it is also possible that tropifexor clearance was decreased in patients with PBC, although no patients with hepatic impairment were included in this study.

FXR target engagement in the intestine and liver was confirmed by dose-dependent increases in FGF19 levels and decreases in C4 levels with greater changes observed on Day 28 than on Day 1. This pharmacodynamic profile in patients with PBC is different from that observed in healthy volunteers, where median change in FGF19 from baseline was comparable on Days 1 and 13.^19^ Nevertheless, the enhanced pharmacokinetic–pharmacodynamic profile resulted in a dose–response relationship among tropifexor, FGF19, and the cholestatic marker GGT.

The main limitation of our study was the relatively short treatment duration of 28 days, unlike other (pivotal) studies with novel agents that span at least 12 weeks of treatment.^36^ Secondly, the number of patients randomised in the individual cohorts of this proof-of-concept study in a rare disease was necessarily low. In the case of tropifexor 150 μg cohort, only 4 patients received all doses. Because the strategic purpose of the study was achieved at the end of part 1, the previously planned 12-week dosing cohort (part 2) was not executed. To better understand the impact of decreases in HDL cholesterol, future studies should include cardiovascular risk assessments. Furthermore, inconsistent changes in itch scores following tropifexor treatment need longer follow-up in larger cohorts. Consistent with previous studies, we used biochemical surrogate endpoints for evaluating the efficacy of tropifexor, but future studies should focus on outcomes-driven endpoints.

Overall, the safety profile of tropifexor was consistent with the known safety profile of FXR agonists. Tropifexor improved liver biomarker levels and demonstrated its anticholestatic potential for treatment in PBC.

The similarity of the anticholestatic effect of tropifexor and OCA in PBC as well as the similarity in the side effects around itch and lipids demonstrated that these effects pertain to FXR agonists as a class of drugs and are not caused by alternative biological actions, given the bile acid structure of OCA *vs*. the non-bile acid structure of tropifexor.

In conclusion, in patients with PBC having an inadequate biochemical response to UDCA, tropifexor was generally safe and well tolerated at daily doses of 30–90 μg; tropifexor showed dose-dependent improvement in cholestatic markers GGT and ALP and the hepatocellular injury marker ALT and had higher exposure than that in healthy volunteers most likely owing to increased absorption. These data support future development of tropifexor for treatment of PBC.

## Financial support

This study was funded by 10.13039/100015311Novartis Institutes for Biomedical Research, Cambridge, USA.

## Authors’ contributions

Study conduct: CS, HW, AM, GMH, KK, PM, EJ, ESM, DJ, JS, PK JC, LBK, MKB. Data interpretation: CS, HW, AM, GMH, KK, PM, EJ, ESM, DJ, CL, JS, PK JC, LBK, MKB, SC. Co-authored the manuscript: CS, HW, AM, GMH, KK, PM, EJ, ESM, DJ, SC, JS, PK JC, LBK, MKB. Study enrolment: CL. Critical review of the manuscript: CL. Study design: JS, PK JC, LBK, MKB. Statistical analysis: SC.

## Data availability statement

The study protocol and the statistical analysis plan can be accessed on ClinicalTrials.gov (https://clinicaltrials.gov/ct2/show/study/NCT02516605). Anonymised patient-level data from clinical trials may be shared by Novartis in a consortium called ClinicalStudyDataRequest.com (CSDR) in accordance with Novartis’ policy for sharing clinical trial data (https://www.clinicalstudydatarequest.com/Study-Sponsors/Study-Sponsors-Novartis.aspx).

## Conflicts of interest

CS is a consultant for Novartis and BiomX and has received honoraria from Falk Pharma and research funding from Galapagos and BiomX. HW is a consultant for or has received speaker fees from Falk Foundation, BMS, AbbVie, Norgine, Merz, Mallinckrodt, MYR GmbH, Gilead, MSD, and Intercept. AM has research grants from Merck and Intercept and has received speaker’s and teaching honoraria from Intercept. GMH has consulted for Intercept, Genfit, Cymabay, GSK, Novartis, Pliant, Falk Pharma. CL has research grants from Novartis, Gilead, Intercept, Enanta, NGM, Genfit, Cara Therapeutics, CymaBay, Target PharmaSolutions, Genkyotex, GSK, Durect, Pliant, High Tide and Zydus, and is a consultant for Genfit, GSK, CymaBay, Mirum, Cara therapeutics, Pliant, Shire, Target PharmaSolutions, Calliditas, and Escient. KVK is on the Advisory Committee or Review Panel of 89Bio, Gilead, CymaBay, Intercept, Genfit, and Madrigal; is a consultant for Calliditas, Intercept, HighTide, Mirum, and Novo Nordisk; has received grant/research support from Allergan, Gilead, Intercept, Genfit, Novartis, Enanta, HighTide, CymaBay, GSK, Pfizer, Madrigal, Viking, Metacrine, Pliant, and Hanmi; has received speaker’s and teaching honoraria from AbbVie, Gilead, and Intercept; and is a stock shareholder of Inipharm. PM has received speaker’s honoraria from Alfa Wasserman and Chiesi. EJ is an investigator in clinical trials sponsored by Allergan, BMS, Celgene, CymaBay, Dr Falk, Gilead, GSK, Janssen, Pfizer, MSD, Novartis, and Roche; has received speaker’s honoraria from AbbVie, BMS, Gilead, Janssen, MSD, and Roche. ESM has no conflicts of interest to disclose. LBK was an employee of Novartis until study completion and is a stockholder of Novartis. DJ has received grant funding from Intercept and Pfizer; is a consultant for Abbott, Genkyotex, GSK and Intercept; and has received speaker fees from Falk, Abbott, and Intercept. JS is a contractor with Novartis. PK, SC, and JC were employees of Novartis until manuscript finalisation. MKB is an employee and stockholder of Novartis.

Please refer to the accompanying ICMJE disclosure forms for further details.
