# Supplementary material for: Farnesoid X receptor agonist tropifexor attenuates cholestasis in a randomised trial in patients with primary biliary cholangitis
Source: JHEP Rep. 2022 Jul 21;4(11):100544. doi: 10.1016/j.jhepr.2022.100544 (PMC9576902; doi:10.1016/j.jhepr.2022.100544)
Supplement: Multimedia component 1 [file mmc1.pdf]

Farnesoid X receptor agonist tropifexor attenuates cholestasis in a randomised trial in patients with primary biliary cholangitis

Christoph Schramm, Heiner Wedemeyer, Andrew Mason, Gideon M. Hirschfield, Cynthia Levy, Kris V. Kowdley, Piotr Milkiewicz, Ewa Janczewska, Elena Sergeevna Malova, Johanne Sanni, Phillip Koo, Jin Chen, Subhajit Choudhury, Lloyd B. Klickstein, Michael K. Badman, David Jones

Table of contents

List of investigators....................2

Table S1....................4

Table S2....................7

Table S3....................9

Table S4....................11

Fig. S1....................13

Fig. S2....................15

Fig. S3....................17

List of investigators

Investigator	Affiliation
**Cynthia Levy**	University of Miami, Schiff Center for Liver Diseases, 1500 NW 12th Avenue, Suite 1101 Miami, FL 33136, USA
**Zeid Kayali**	Inland Empire Liver Foundation, 2006 N Riverside Ave, Rialto, CA 92377, United States
**Kris V Kowdley**	Liver Institute Northwest, Washington State University, Seattle, WA 98104, USA
**David Bernstein**	Northwell Health – Sandra Atlas Bass Center for Liver Diseases, 400 Community Drive, 1st Floor Manhasset, NY 11030, United States
**Aasim Sheikh**	Gastrointestinal Specialists of Georgia, 711 Canton Rd NE Suite 300, Marietta, GA 30060, United States
**Norman Gitlin**	Atlanta Gastroenterology Associates LLC, 2520 Windy Hill Rd SE Ste 102, Marietta, GA, 30067, United States
**Nancy Reau**	Rush University Medical Center, 725 W Harrison St Suite 158, Chicago, IL 60612, United States
**David Jones**	The Newcastle Upon Tyne Hospitals, NHS Foundation Trust, Royal Victoria Infirmary, Queen Victoria Road, Newcastle, United Kingdom
**Gideon Hirschfield**	Toronto Centre for Liver Disease, Department of Medicine, University of Toronto, Toronto, ON, Canada
**George Mells**	Addenbrooke’s Hospital, Hills Rd, Cambridge CB2 0QQ, United Kingdom
**Douglas Thorburn**	Royal Free Hospital, Institute of Liver & Digestive Health, Rowland Hill Street, London NW3 2PF, United States
**Christian Rupp**	Medizinische Universitätsklinik, 79410, Badenweiler , Baden-Württemberg, Germany
**Christoph Schramm**	Medizinische Klinik und Poliklinik Universitätsklinikum Hamburg Eppendorf, Martinistraße 52, Hamburg 20246, Germany
**Hans Heinrich Wedemeyer**	Department of Gastroenterology and Hepatology, Essen University Hospital, Hufelandstraße 55, 45147 Essen, Germany
**Gerald Denk**	Medizinische Klinik II Klinikum der Universität München-Groβhadern, Marchioninistraße 15, 81377 München, Germany
**Andreas Geier**	Medizinische Klinik und Poliklinik II Zentrum Innere Medizin, 38120, Braunschweig , Niedersachsen Germany
**Andrew Mason**	Division of Gastroenterology, University of Alberta, Edmonton, AB, Canada

**Mark Swain**	Division of Gastroenterology, Immunology and Gastrointestinal Research Group, University of Calgary, 3350 Hospital Dr NW, Calgary, AB T2N 4Z5, Canada
**Piotr Milikiewicz**	Liver and Internal Medicine Unit, Medical University of Warsaw, Banacha1A, 02-097 Warsaw, Poland
**Ewa Janczewska**	ID Clinic, 19, Janowska Str 41-400, Myslowice Poland
**Jaroslaw Leszczyszyn**	Melita Medical, Generała Romualda Traugutta 1/7, 50-449 Wrocław, Poland
**Iurii Kravchuk**	Military Medical Academy, Akademika Lebedeva Street, 6, Saint-Petersburg 194044, Russian Federation
**Elena Sergeevna Malova**	LLC Medical Company Hepatolog, Serdobskaya street, 36A, Samara 443063, Russian Federation

Table S1. Eligibility criteria

Inclusion criteria	Exclusion criteria
• Written informed consent obtained before any assessment	• Presence of other concomitant liver diseases including:<br>o Active HBV or HCV infection<br>o PSC<br>o Alcoholic liver disease<br>o Definite autoimmune hepatitis<br>o NASH<br>o Gilbert's syndrome
• Age ≥ 18 years	• Women of child-bearing potential, defined as all women physiologically capable of becoming pregnant, unless they were using highly effective methods of contraception for 30 days before randomisation, and would continue to use it during dosing and for 30 days following the end of treatment
• Diagnosis of PBC as demonstrated by the presence of at least 2 of the following 3 diagnostic criteria:<br>o History of ALP elevated above ULN for ≥6 months<br>o Positive AMA titre or if AMA negative or in low titre (<1:80) PBC-specific antibodies (anti-GP210 and/or anti-SP100 and/or antibodies against the major M2 components [PDC-E2, 2-oxo-glutaric acid dehydrogenase complex])<br>o Previous liver biopsy findings consistent with PBC	• Cirrhosis with complications, including history or presence of:<br>o Variceal bleed<br>o Uncontrolled ascites<br>o Encephalopathy<br>o Spontaneous bacterial peritonitis
• At least 1 of the following markers of disease severity:<br>o ALP ≥1.67× ULN<br>o Total bilirubin >1× ULN but <1.5× ULN<br>In addition, patients must meet following biochemical criteria at enrolment:	• Significant hepatic impairment as defined by Child-Pugh classification of B or C, or history of liver transplantation, current placement on a liver transplant list or current MELD score ≥15

▪ ALT or AST ≤5× ULN<br>▪ Total bilirubin ≤1.5× ULN<br>▪ INR ≤ 1× ULN	
• UDCA use for ≥12 months, or for ≥6 months and attainment of maximal response to UDCA with a plateau in ALP, with no changes in dose for ≥3 months prior to Day 1	• History of medical conditions other than PBC that could cause increase in ALP (e.g., Paget's disease)
• Weight ≥40 kg with BMI range of 18-40 kg/m^2	• History of malignancy of any organ system (other than localized basal cell carcinoma of the skin or treated cervical intraepithelial neoplasia), treated or untreated, within the past 5 years, regardless of whether there is evidence of local recurrence or metastases
• Able to communicate well with the investigator to understand and comply with the requirements of the study	• Sexually active males were instructed to use a condom during intercourse while taking drug and for 5 days after stopping investigational medication and told not father a child in this period. A condom was required to be used also by vasectomised men in order to prevent delivery of the drug via seminal fluid
	• History of non-adherence to medical regimens, or patients who were considered to be unable to reliably comply with the requirements of the study
	• Donation or loss of ≥400 mL of blood within 8 weeks prior to initial dosing, or longer if required by local regulation
	• Use of other investigational drugs or immunosuppressive drugs at the time of enrolment, or within 5 half-lives/30 days of randomisation, whichever is longer; (or longer if required by local regulations). Use of high dose oral steroids to treat co-morbid conditions (e.g., airways disease) were allowed but was required to be properly documented as such in concomitant medications
	• Pregnant or nursing (lactating) women, where pregnancy is defined as the state of a female after conception and until the

	termination of gestation, confirmed by a positive hCG laboratory test
	• History of drug or alcohol abuse within the 12 months prior to dosing
	• Significant illness which has not resolved within 2 weeks prior to initial dosing
	• Acute or chronic renal disease with a screening serum creatinine >1× ULN
	• Elevated liver function tests at screening defined as follows:<br>○ Potential Hy's Law case (defined as ALT or AST >3× ULN and total bilirubin >2× ULN without notable increase in ALP to >2× ULN) or<br>○ ALT or AST > 3× ULN combined with INR >1.5 or<br>○ Total bilirubin >1× ULN combined with albumin outside of the normal range
	• Currently taking obeticholic acid or have taken obeticholic acid within 30 days of randomisation
	• Participated in the current study and received study medication within 3 months of randomisation (or longer if required by local regulations)
	• History of immunodeficiency diseases, including a positive HIV (ELISA and Western blot) test result

ALP, alkaline phosphatase; ALT, alanine aminotransferase; AMA, antimitochondrial antibodies; AST, aspartate aminotransferase; BMI, body mass index; ELISA, enzyme-linked immunosorbent assay; HBV, hepatitis B virus; hCG, human chorionic gonadotropin; HCV, hepatitis C virus; HIV, human immunodeficiency virus; INR, international normalised ratio; MELD, model for end-stage liver disease; NASH, non-alcoholic steatohepatitis; PBC, primary biliary cholangitis; PSC; primary sclerosing cholangitis; PDC-E2, pyruvate dehydrogenase complex-E2; UDCA, ursodeoxycholic acid; ULN, upper limit of normal

Table S2. Statistical analysis methods

Assessment	Methodology
Sample size calculation	With 12 patients per cohort randomised to tropifexor or placebo with a randomisation ratio of 2:1, there was approximately 85% probability that the upper limit of a 90% two-sided CI of treatment difference between tropifexor versus placebo would exclude 0, if the underlying treatment difference was about −50% in terms of percent change from baseline, assuming a standard deviation of 28%. A total of up to 5 cohorts of tropifexor at different dose levels could be studied. Assuming a dropout rate of approximately 20%, 15 patients per cohort were to be enrolled, with a total of at least 30 (up to 75) patients.
Primary endpoint	Serum GGT values at all time points were logarithmically transformed and change from baseline was calculated as the difference between each of the log-transformed post-dose serum GGT values and the log-transformed baseline serum GGT value and converted to percent change from baseline. Log-transformed ratio to baseline was analysed by repeated measures analysis of covariance. The model included treatment as a fixed effect, time as a repeated effect, the treatment by time interaction, the time by log-transformed baseline interaction and log-transformed baseline as a covariate. An unstructured covariance matrix was used. Missing values were not imputed. A 2-sided 90% CI for the difference between a treatment and placebo was reported for each visit. This was then "back-transformed" to the original scale to give a fold decrease from baseline.
Secondary efficacy	ALP, ALT and total bilirubin data were analysed using the same approach as described for GGT.<br><br>The difference between tropifexor and placebo at Day 28 was compared by Wilcoxon rank sum test for change from baseline in PBC-40 subdomain scores. A 2-sided P value and 90% CI for a treatment difference was reported with no adjustment for multiplicity.<br><br>The change from baseline in VAS score for itch was analysed using the same repeated measures analysis of covariance as described for GGT with baseline replacing log-transformed baseline in the model.

Pharmacokinetics	At Day 1 and Day 28, AUC and C_{max} were summarized for each dose along with 90% 2-sided CIs computed on a log-scale and back-transformed. Dose-proportionality was explored by fitting a power model (PK parameter = α dose β). A natural logarithmic transformation was applied for the power model.
Pharmacodynamics	For exploratory biomarker C4, changes from baseline (pre-dose on Day 1) were calculated. The minimum observed concentration (C_{min}), the minimum change from baseline and AUC were also calculated on Day 1 and Day 28. The Wilcoxon rank-sum test was used for FGF19 and C4 data at each day and time point, to compare the median difference of changes from baseline for tropifexor dose and pooled placebo. The Hodges-Lehmann estimate and 90% CI for the median difference were provided. Similarly, derived parameters AUC_{0-8h} and C_{max} for FGF19 were also analysed.

AUC_{0-8h}, area under the plasma concentration-time curve from time 0 to 8 h; CI, confidence interval; C_{max}, observed maximum plasma concentration following drug administration; C_{min}, trough concentration; FGF19, fibroblast growth factor 19; GGT, γ-glutamyl transferase

Table S3. Summary of plasma PK parameters of tropifexor following daily doses of tropifexor (PK analysis set)

Tropifexor qd dose (µg)		PK parameters									
		$AUC_{0\text{-}8h}$ (ng*h/mL)		C_{max} (ng/mL)		T_{max} (h)**		CL_{ss}/F (mL/h)		R_{acc}	
		n	Mean (SD)	n	Mean (SD)	n	Median [range]	n	Mean (SD)	n	Mean (SD)
30	Day 1	7	4.98 (2.870)	10	1.04 (0.484)	10	4.12 [2.00-8.00]	1	8000	7	1.0 (0)
	CV%		57.6		46.7				-		-
	Day 28	9	7.95 (4.210)	11	1.25 (0.559)	11	4.08 [2.00-8.00]	8	2710 (2210)	7	1.50 (0.575)
	CV%		52.9		44.7				81.7		38.3
60	Day 1	3	12.10 (2.680)	7	1.80 (0.585)	7	4.00 [3.70-6.00]	-	-	3	1.0 (0)
	CV%		22.3		32.5				-		-
	Day 28	7	17.60 (5.300)	9	2.55 (0.946)	9	4.00 [3.13-7.60]	3	1340 (186)	3	1.72 (0.230)
	CV%		30.1		37.1			-	13.9		13.3
90	Day 1	-	-	12	2.37 (1.560)	12	4.00 [0.00-7.83]	-	-	-	-
	CV%		-		65.5				-		-
	Day 28	3	23.40 (8.700)	12	4.30 (2.100)	12	4.00 [0.00-6.00]	3	1730 (810)	-	-
	CV%		37.2		48.8				46.8		-
150	Day 1	4	24.50 (15.900)	8	4.84 (2.590)	8	4.00 [4.00-4.18]	-	-	4	1 (0)
	CV%		64.9		53.5				-		-
	Day 28	3	44.20 (25.800)	4	6.37 (3.400)	4	5.0 [3.03-6.03]	1	2130	2	1.63 (0.144)
	CV%		58.3		53.3				-		8.8

**T_{max} is represented as median and range

$AUC_{0\text{-}8h}$, area under the plasma concentration-time curve from time 0 to 8 h; C_{max}, observed maximum plasma concentration following drug administration; CL_{ss}/F, apparent systemic clearance from plasma observed during a dosing interval at steady state following oral administration; CV%, % coefficient of variation; PK, pharmacokinetics; qd, once daily; R_{acc}, accumulation ratio; SD, standard deviation; T_{max}, time to reach C_{max}

Table S4. Non-parametric analysis of PD biomarkers following daily doses of tropifexor (PD analysis set)

FGF19		n	Median AUC_{0-8h} (pg*h/mL)	Median difference vs placebo (90% CI)
30 µg	Day 1	9	2145.6	1231.9 (544.98, 3202.68)
	Day 28	9	2575.5	975.0 (339.10, 2415.05)
60 µg	Day 1	6	3851.8	2575.9 (−181.95, 3815.75)
	Day 28	5	4365.9	3369.5 (1626.36, 4546.81)
90 µg	Day 1	2	2776.5	1483.5 (−2455.93, 3274.97)
	Day 28	2	2567.8	1333.0 (14.61, 2581.05)
150 µg	Day 1	4	5044.1	3495.9 (326.48, 29853.93)
	Day 28	2	32685.9	30912.6 (−1183.24, 64014.95)
			**C_{max} (pg/mL)**	**Median difference vs placebo (90% CI)**
30 µg	Day 1	10	550.5	310.3 (202.00, 524.70)
	Day 28	10	493.5	285.1 (160.00, 501.50)
60 µg	Day 1	9	726.0	461.2 (284.00, 877.00)
	Day 28	9	1110.0	797.0 (253.00, 1143.30)
90 µg	Day 1	12	569.5	270.1 (40.40, 827.00)
	Day 28	12	858.0	583.3 (402.00, 745.00)
150 µg	Day 1	8	518.5	266.5 (102.00, 654.00)
	Day 28	5	755.0	463.5 (−40.00, 777.20)
**C4**			**Median AUC_{0-8h} (ng*h/mL)**	**Median difference vs placebo (90% CI)**
30 µg	Day 1	9	103.7	−48.0 (−141.16, 52.37)
	Day 28	9	77.2	−72.8 (−148.89, 3.39)
60 µg	Day 1	6	79.3	−96.8 (−206.84, 21.72)
	Day 28	5	37.5	−109.9 (−202.35, 8.75)
90 µg	Day 1	3	86.0	−58.0 (−189.45, 14.94)
	Day 28	2	42.7	−130.3 (−360.76, −19.90)
150 µg	Day 1	4	80.4	−106.2 (−223.88, −2.51)
	Day 28	2	46.3	−125.6 (−325.98, 14.89)

$AUC_{0\text{-}8h}$, area under the concentration time curve from 0 to 8 hours; C4, 7-alpha-hydroxy-4-cholesten-3-one; CI, confidence interval; C_{max}, observed maximum plasma concentration; D, day; FGF19, fibroblast growth factor 19; PD, pharmacodynamics

Fig S1. Study design

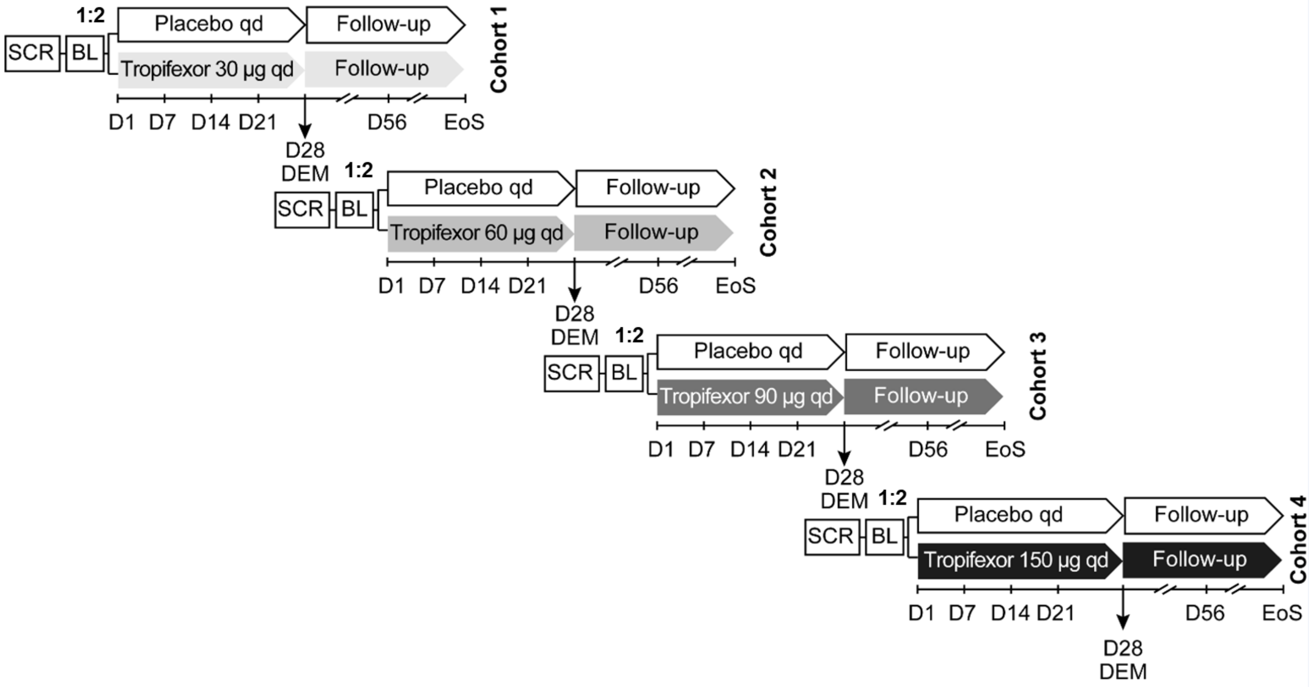

This study was originally designed in 2 parts. Part 1 comprised of an escalating multiple dose design in PBC patients with incomplete biochemical response to, but still taking, ursodeoxycholic acid (UDCA). A second part of the study (Part 2) was designed as a parallel-group, 12-week study to assess the safety, tolerability and efficacy of two doses of tropifexor compared to placebo in patients with PBC having an incomplete biochemical response to, but still taking, UDCA or those not currently taking UDCA. The two tropifexor doses for Part 2 were to be selected based on safety, tolerability and efficacy data from Part 1. However, the study was terminated after Part 1 because data revealed that Part 1 fulfilled the strategic purpose of the study. In Part 1, 4 cohorts of approximately 15 patients each were planned to be enrolled. Each patient was to undergo screening visit, baseline assessments, 4-week treatment period, follow-up period and EoS visit. Patients in Cohort 1 were randomised in a 2:1 ratio to receive once daily starting dose of 30 µg tropifexor or placebo. Safety assessments were

performed for 4 hours post dose and pharmacokinetic and biomarker assessments were performed for up to 8 hours post first dose. Prior to discharge, the patients were given a supply of tropifexor or placebo and instructed to take their dose first thing in the morning with water, prior to eating, every day. Patients were advised to return on Days 7, 14 and 21 for safety, pharmacokinetic and biomarker assessments and dosing and on Day 28, for the final dosing visit, when safety, pharmacokinetic and biomarker assessments were to be performed. A dose escalation review and an interim analysis were performed when at least 12 patients completed dosing and Day 28 assessments in Cohort 1. The main purpose of the interim analysis was to evaluate the observed variability in GGT levels and, if needed, consider a sample size re-estimation, together with reviewing the safety and tolerability from the cohort. Additional cohorts followed the same schedule as Cohort 1. Further cohorts were added depending on emerging safety and pharmacokinetic data. Additional interim analyses could be performed after a minimum of 12 patients in each cohort finished dosing and Day 28 assessment.

BL, baseline; D, day; DEM, dose escalation meeting; EoS, end of study; qd, once daily; SCR, screening

Fig. S2. Patient disposition

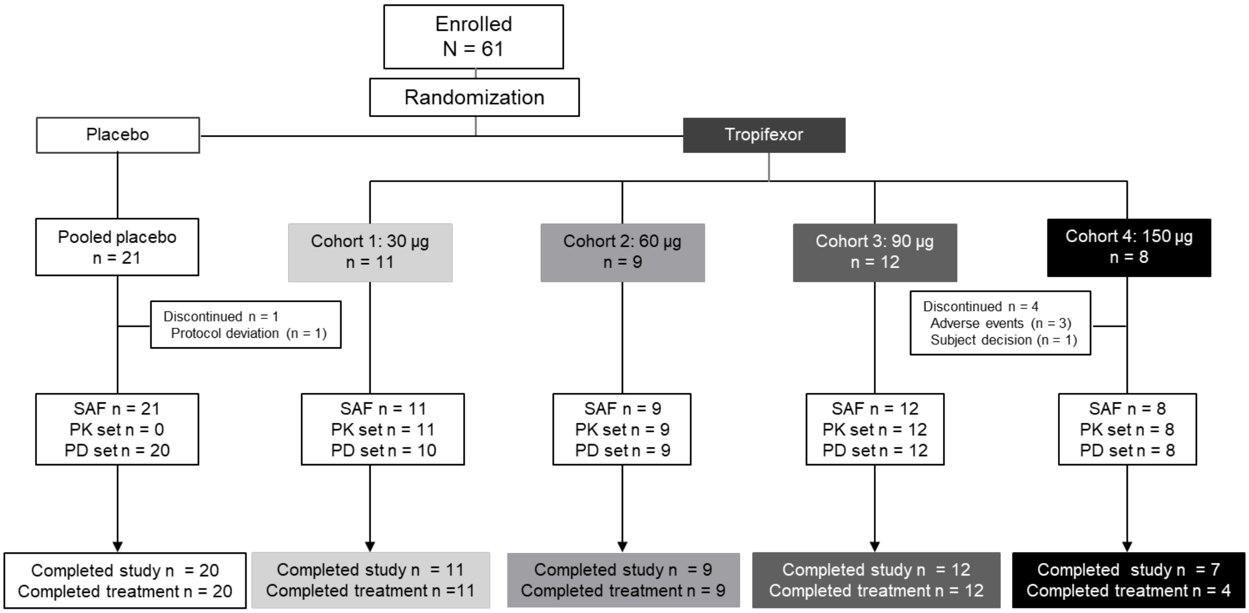

In cohort 1, 11 patients received 30 µg tropifexor and 5 patients received placebo; of these, 1 patient was incorrectly dosed with 100 µg tropifexor on Day 1 and was excluded from the PD analysis set. In cohort 2, 9 patients received 60 µg tropifexor, of which one patient was incorrectly dosed with 30 µg tropifexor on Day 1. Six patients received placebo with the exception of 1 patient who was discontinued from the study due to protocol deviation. In cohort 3, 12 patients received 90 µg tropifexor and 6 patients received placebo. Out of 8 patients randomised to receive 150 µg tropifexor in cohort 4, only 4 received all doses of study treatment. Out of the remaining 4, 1 patient received only 12 days of tropifexor treatment and was discontinued from treatment thereafter due to elevated AST; 1 received 11 days of tropifexor treatment and was discontinued from treatment thereafter due to AEs pruritus, insomnia, and proteinuria; 1 received 8 days of tropifexor treatment and was discontinued thereafter due to subject

decision; and 1 received only 8 days of tropifexor treatment and was discontinued thereafter due to the AE pruritus. Four patients received placebo in this cohort.

AEs, adverse events; PD, pharmacodynamics; PK, pharmacokinetics; SAF, safety analysis set

Fig. S3. Changes in biomarker levels

A)

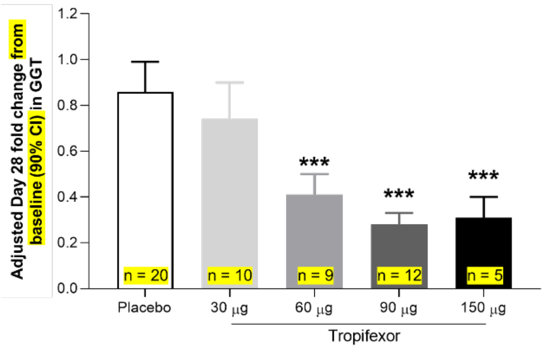

B)

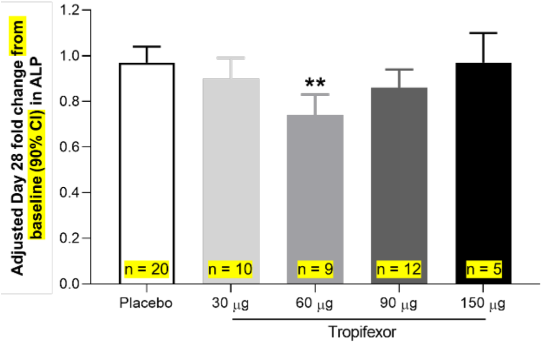

C)

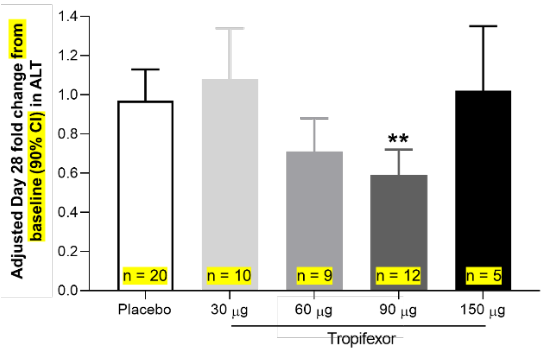

D)

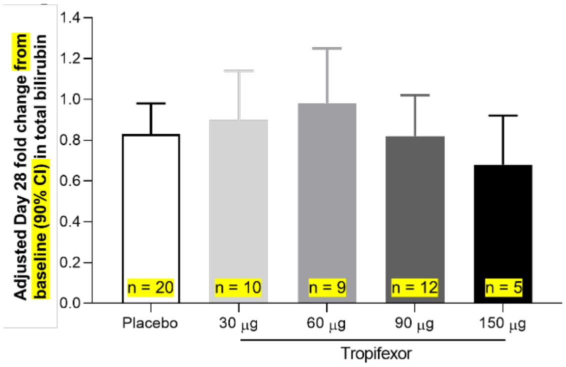

Adjusted Day 28 fold change from baseline in (A) GGT, (B) ALP, (C) ALT, and (D) total bilirubin. **P*<0.05, ***P*<0.01, and ****P*<0.001 compared with placebo

ALP; alkaline phosphatase; ALT, alanine aminotransferase; CI, confidence interval; GGT, γ-glutamyl transferase.
