# Supplementary material for: Farnesoid X receptor agonist tropifexor attenuates cholestasis in a randomised trial in patients with primary biliary cholangitis
Source: JHEP Rep. 2022 Jul 21;4(11):100544. doi: 10.1016/j.jhepr.2022.100544 (PMC9576902; doi:10.1016/j.jhepr.2022.100544)
Supplement: Multimedia component 2 [file mmc2.pdf]

NOVARTIS

Biostatistics & Statistical Programming /
Novartis Institutes for BioMedical Research

Tropifexor (LJN452)

Study CLJN452X2201 / NCT02516605

A multi-part, randomized, double-blind, placebo-controlled study to assess the safety, tolerability and efficacy of tropifexor (LJN452) in patients with Primary Biliary Cholangitis

RAP Module 3: Detailed Statistical Methodology

Author(s): [redacted], Trial Statistician ([redacted])
[redacted], Trial Statistician (Novartis)

Document type: RAP Module 3: Detailed Statistical Methodology

Document status: Final – Amendment 1

Property of Novartis
Confidential
May not be used, divulged, published or otherwise disclosed
without the consent of Novartis

Table of contents

Table of contents 2
List of tables 3
1 Introduction to RAP documentation 4
1.1 Scope 4
1.2 Changes to RAP documentation (M3) 4
2 Study objectives and design 5
2.1 Study objectives 5
2.1.1 Primary Objectives 5
2.1.2 Secondary Objectives 5
[redacted] 5
2.2 Study design and treatment 5
3 First interpretable results (FIR) 8
4 Interim analyses 8
5 Statistical methods: Analysis sets 10
6 Statistical methods for Pharmacokinetic (PK) parameters 11
6.1.1 Variables 11
6.1.2 Descriptive analyses 11
6.1.3 Statistical model, assumptions and hypotheses 12
6.1.4 Model checking procedures 12
6.1.5 Graphical presentation of results 12
6.1.6 Pharmacokinetic / pharmacodynamic interactions 13
7 Statistical methods for Pharmacodynamic (PD) parameters 13
7.1 Primary objective 13
7.1.1 Variables 13
7.1.2 Descriptive analyses 13
7.1.3 Statistical model, assumptions and hypotheses 13
7.1.4 Handling of missing values/censoring/discontinuations 14
7.1.5 Model checking procedures 14
7.1.6 Graphical presentation of results 14
7.1.7 Supportive analyses 14
7.2 Secondary objectives 15
7.2.1 Variables 15
7.2.2 Descriptive analyses 15
7.2.3 Statistical model, assumptions and hypotheses 15
7.2.4 Model checking procedures 15
7.2.5 Graphical presentation of results 15

[redacted] 16
8 Statistical methods for safety and tolerability data 16
8.1.1 Variables 16
8.1.2 Descriptive analyses 16
8.1.3 Graphical presentation of results 17
[redacted] 17

List of tables

Figure 2-1 Study design 6
Figure 2-2 Study design Part 1 7
Figure 2-3 Study design Part 2 8
Table 5-1 Protocol deviation codes and analysis sets 10

1 Introduction to RAP documentation

1.1 Scope

The RAP documents contain detailed information to aid the production of Statistics & Programming input into the Clinical Study Report (CSR) for trial **"CLJN452X2201"**.

Module 3 (M3) provides the description of the statistical methodology used to analyze the data, **TFL shells** details the presentation of the data, including shells of summary tables, figures and listings, and **Module 8** (M8) contains programming specifications e.g. for derived variables and derived datasets, to support the creation of CSR outputs.

1.2 Changes to RAP documentation (M3)

Refer to corresponding guidances and NIBR RAP Addendum template for detailed information on the requirements of documenting changes to RAP documentation.

For the statistical methodology (M3), any major changes to the statistical methodology should be reflected in the RAP M3 documentation via version control (M3 amendment) (new document version to be approved by the trial team as the original module).

Such major changes could include (but are not limited to):

- change in statistical methodology
- substantial change in (derivation of) main endpoint
- substantial change in study design (e.g. protocol amendment introducing new multiple-dose cohorts in a so far single-dose trial)

Such changes may also require a protocol amendment to ensure consistency. In addition they need to be mentioned (high-level) in the CSR (section for changes to planned analysis).

Minor changes to the RAP M3 documentation can be captured e.g. by a study note to file / note in RAP Addendum or within the CSR itself. [redacted] Corrections of typographical errors or modification of spelling (from English to American, for example) do not need to be incorporated into the RAP M3 documentation.

[redacted]

This RAP M3 Amendment 1 is to reflect the following changes:

- The addition of a deviation code leading to exclusion from the PD analysis set in Table 5-1.
- The addition of the time by log-transformed baseline term in the repeated measures analysis model in Section 7.1.3.
- The changes to the study design in protocol amendment 5.

2 Study objectives and design

2.1 Study objectives

2.1.1 Primary Objectives

- To determine the effect of LJN452 on cholestatic markers (Liver function tests, specifically GGT) in patients with Primary Biliary Cholangitis (PBC). More specifically, in Part 2 to determine the dose-response relationship of LJN452 on GGT following 12 weeks of treatment
- To determine the safety and tolerability of daily dosing of LJN452 in patients with PBC

2.1.2 Secondary Objectives

- To evaluate the pharmacokinetics (PK) of LJN452 in patients with PBC
- To evaluate the change in overall disease specific quality of life from PBC40
- To determine the change in the itch domain of PBC40 questionnaire
- To evaluate the change in itch based on 100 mm visual analog score (VAS)
- Part 2: To determine the dose-response relationship of LJN452 on ALP in patients with PBC following 12 weeks of treatment

[redacted]

2.2 Study design and treatment

This is a randomized, double-blind, placebo-controlled, multi-part study to assess safety, tolerability and efficacy of LJN452 in patients with Primary Biliary Cholangitis (PBC).

Part 1 uses an escalating multiple dose design in PBC patients with incomplete biochemical response to, but still taking, ursodeoxycholic acid (UDCA).

Part 2 is a parallel group, 12-week study to assess the safety, tolerability and efficacy of two doses of LJN452 compared to placebo in PBC patients with an incomplete biochemical response to, but still taking, UDCA or those not taking UDCA. The two LJN452 doses for Part 2 will be selected based on safety, tolerability and efficacy data from Part 1.

Once approximately 40 patients from Part 2 have completed 8 weeks of treatment, an interim analysis will be conducted to re-assess the sample size for Part 2. Figure 2-1 shows how Parts 1 and 2 are connected.

Figure 2-1 Study design

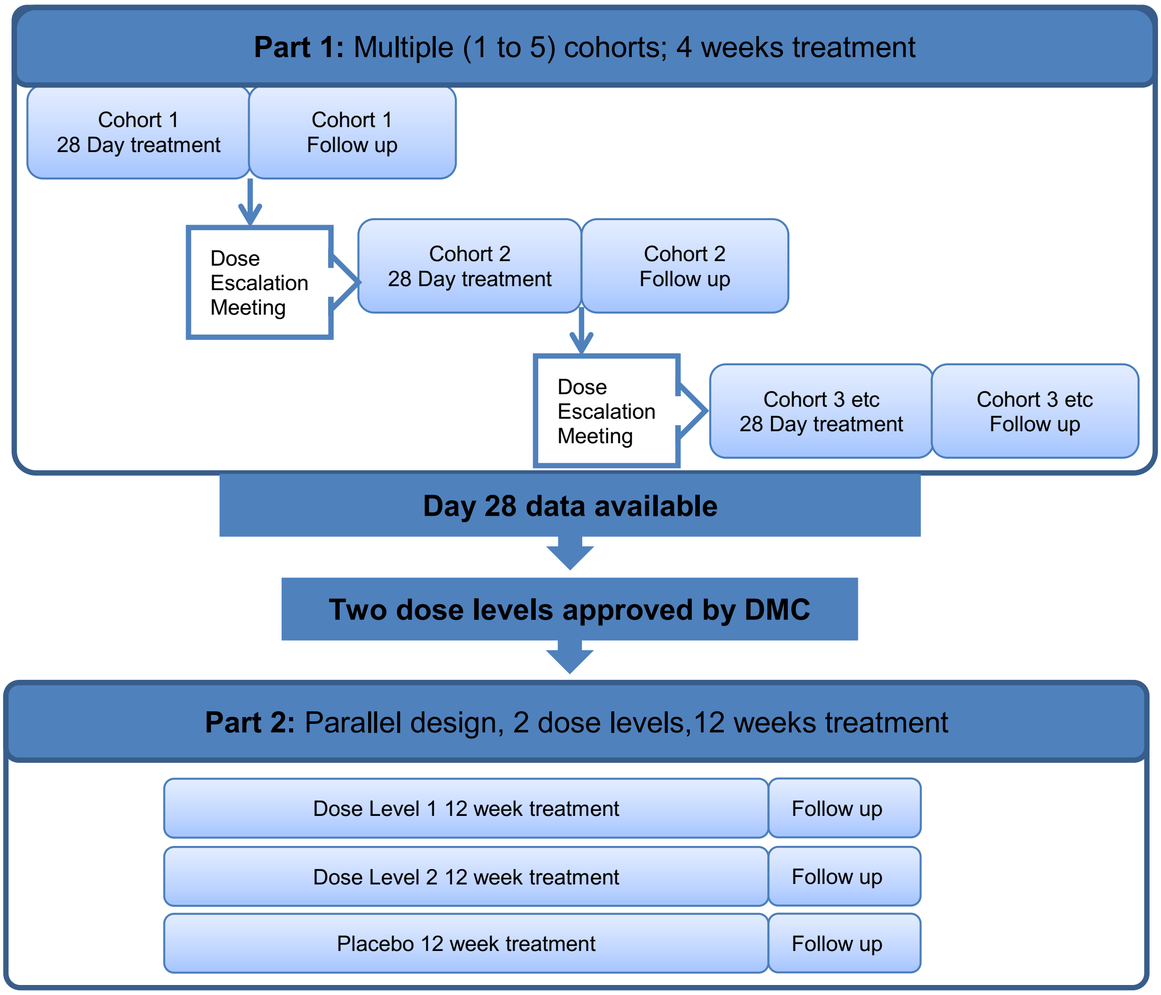

Part 1

A minimum of 2 and maximum of 5 cohorts of approximately 15 patients with PBC with incomplete biochemical response, but still taking UDCA treatment will be enrolled into Part 1, with the aim of a minimum of 12 patients per cohort completing. Patients withdrawing for reasons other than AEs may be replaced throughout the study. Patients withdrawing from a cohort may not be enrolled in a subsequent cohort.

Figure 2-2 shows the study design of Part 1. At the beginning of Part 1, a screening visit will take place, where a patient's eligibility to enter the study will be assessed. Eligible patients will be admitted to the study site and re-evaluated for inclusion / exclusion criteria during the baseline visit. All baseline safety evaluation results must be available and reviewed prior to first dosing.

Figure 2-2 Study design Part 1

Study Day		1	7	14	21	28	29	56	84
Study visit ↑	↑	↑	↑	↑	↑	↑	↑	↑	↑
Screening	BL	<- 4 week treatment period ->					Follow up		EOS

BL = Baseline assessments
EOS = End of Study visit
↑ = Study visit ↑ = Optional study visit

Patients will be asked to arrive at the study site approximately 2 hours prior to dosing on Day 1. Patients in Cohort 1 will be randomized in a 2:1 ratio to receive the starting dose of 0.03 mg LJN452 or placebo. Safety assessments will be performed for 4 hours post dose and pharmacokinetic [redacted] assessments will be performed for up to 8 hours post first dose. Prior to discharge, the patients will be given a supply of LJN452 or placebo and instructed to take first thing in the morning with water, prior to eating every day.

Patients will then return on Days 7, 14 and 21 for safety, pharmacokinetic [redacted] assessments and dosing. Patients will be asked to return to the study site on Day 28, for the final dosing visit, when safety, pharmacokinetic [redacted] assessments will be performed. Patients will be asked to return to the study site for an optional ambulatory visit on Day 29, where 24 hour post dose pharmacokinetic [redacted] assessments will be performed.

Patients are asked to return to the study site on approximately Day 56 for follow up assessments. An end of study visit will take place at approximately Day 84.

A dose escalation review and an interim analysis are planned when at least 12 patients have completed dosing and Day 28 assessments in Cohort 1. The main purpose of the interim analysis will be to evaluate the observed variability in GGT levels and, if needed, consider a sample size re-estimation.

Additional cohorts will follow the same schedule as Cohort 1. Further cohorts may be added depending on emerging safety and PK/PD data. Additional interim analyses may be performed after a minimum of 12 patients in each cohort finish dosing and Day 28 assessment. The sponsor will be unblinded and the Clinical Trial Team may be unblinded when all subjects in a cohort have completed 28 day of study hence at the time of the planned IAs.

Part 2

Approximately 88 patients with PBC, with an incomplete biochemical response to, but still taking, UDCA or those not currently taking UDCA are planned to be recruited into Part 2, with the aim that 80 will complete the study. Patients who participated in Part 1 may be recruited to Part 2, provided they meet all required eligibility criteria and have not received study medication within 3 months of Randomization (or longer if required by local regulations).

Figure 2-3 Study design Part 2

Study visit ↑	↑	TC	↑		↑				↑				↑	↑
Screening	<----- BL	---------- 12 week treatment period ---------------->												Follow up EOS

BL = Baseline assessments (performed pre-dose Day 1)
↑ = Study visit
TC = Telephone Contact
EOS = End of Study visit

A screening visit will take place, where patient's eligibility to enter the study will be assessed.

Eligible patients will be asked to arrive at the study site approximately 2 hours prior to dosing on Day 1 to perform Baseline assessments.

Patients will be randomized in a 3:3:2 ratio to receive either LJN452 Dose level 1, LJN452 Dose level 2 or placebo. Following drug administration, safety, pharmacokinetic [redacted] assessments will be performed at 2 hours post dose.

Prior to discharge, the patients will be given a supply of LJN452 or placebo and instructed to take their dose first thing in the morning with water, prior to eating every day.

Patients will then return at weeks 3, 5, 9 and 13 for safety, tolerability, pharmacokinetic [redacted] assessments. A telephone contact will be made one week after start of dosing to check their progress and check they have no questions.

Patients will be asked to return to the study site in week 17 for follow up assessments and to complete their end of study assessments.

3 First interpretable results (FIR)

First interpretable results (FIR) will be provided for this trial.

The study FIR template (mock slides) can be found in CREDI in the study RAP folder.

The template shows the analysis / results to be presented in the FIR. Study outputs required to be created at the time of the FIR will be highlighted in TFL shells document and marked as “Key” in the Programming Deliverables Tracker (PDT) output list.

4 Interim analyses

The first interim analysis will be performed when at least 12 patients in Cohort 1 have completed 4 weeks of dosing at 0.03 mg and Day 28 post dose assessments. The purpose of this interim analysis is to assess patient safety, to evaluate the assumption on intra-subject variability of GGT, which was used for sample size calculations, and to select a dose for the next cohort. An ANCOVA model will be fitted to estimate the change from baseline in GGT and its variability, and an unblinded sample size re-estimation conducted. The results may be used to adjust the size of the next cohort.

Doses in subsequent cohorts of Part 1 will be decided based on accumulated information collected from all previous cohorts including safety, PK and PD data.

Additional interim analyses will be performed when at least 12 patients in subsequent cohorts of Part 1 have completed 4 weeks of dosing and Day 28 assessments. Placebo data from all cohorts will be pooled in the ANCOVA model to evaluate the treatment difference between each dose level of LJN452 versus placebo. The model described in the primary analysis section will be fitted, as it is indeed the same analysis which will be included in the final study report. [redacted]

Results from Part 1 will be used to select the doses for Part 2, which may be initiated prior to the completion of Part 1. Part 2 will not be initiated if the estimated change from baseline in GGT at Day 28 is smaller than 20% at the highest dose level of LJN452 in Part 1.

Unblinded interim analysis results from Part 1 will be reviewed by an internal Novartis Interim Analysis Team which may include some CTT members. The Interim Analysis Team may communicate interim results to relevant Novartis teams for information, consulting and/or decision purposes. The composition of the interim analysis team will be decided before the FPFV and a roster of membership maintained in the trial master file.

The Interim Analysis Team may communicate interim results (e.g., evaluation of PoC criteria or information needed for planning/modifying another study) to relevant Novartis teams for information, consulting and/or decision purposes.

No further dissemination of interim results based on un-locked data should occur, in particular not with individuals involved in treating the study's subjects or assessing clinical data (e.g., ECGs, images, symptoms) obtained in the study.

In Part 2 of the study, an interim analysis is planned when approximately 40 patients have completed their Week 8 visit. The purpose of this interim analysis is to review the sample size (calculated based on GGT and ALP changes) to assess if it is also adequate to provide precise enough treatment difference estimates in itch parameters, a key tolerability endpoint. A blinded sample size re-estimation will be performed and a proposal to increase the sample size submitted to the DMC as needed. The DMC will decide whether it is appropriate to increase the sample size based on an unblinded interim analysis, which will be performed by a study independent statistician and programmer with outputs provided to the DMC only. Recruitment will continue while the IA is being performed.

This study will utilize a staggered lock of the clinical database, so that the data can be locked after completion of Part 1, or a set of Cohorts in Part 1.

Once a Cohort's data has been locked, treatment codes for the relevant cohort(s) will be released and made available for data analysis.

The results from the locked Part 1 interim analysis can be communicated beyond Novartis to groups including, but not limited to individuals treating the study's subjects, health Authorities and reported on clinical registries.

Additional interim analyses [redacted] may be conducted to support decision making concerning the current clinical study, the sponsor's clinical development projects in general or in case of any safety concerns.

No dose escalation is planned in Part 2.

DMC

In Part 2, in addition to frequent review of the emerging study data by the study team, the DMC will review safety, including AEs and laboratory parameters, on a regular basis. In addition, in the event that more than 3 patients develop an AE of CTCAE Grade 3 or higher in the same system organ class, the DMC Chair will be alerted and may call an *ad hoc* DMC meeting. Further details regarding relevant data and actions will be specified in the separate DMC charter. The DMC may call for the study to be placed on hold to further enrollment, and the study may be stopped based on a full review of all available clinical safety data.

The DMC charter contains specific details and outputs for the DMC are contained in a separate document.

5 Statistical methods: Analysis sets

For all analysis sets, subjects will be analyzed according to the study treatment(s) received. Subjects with dose reduction due to ALT/AST will be analyzed according to the treatment they received up to the dose reduction in Part 2.

The safety analysis set will include all subjects that received any study drug and experienced no protocol deviations with relevant impact on safety data.

The PK analysis set will include all subjects with at least one available valid (i.e., not flagged for exclusion) PK concentration measurement, who received any study drug and experienced no protocol deviations with relevant impact on PK data.

The PD analysis set will include all subjects with available PD data and no protocol deviations with relevant impact on PD data.

The analysis sets and protocol deviation codes are related as follows:

Table 5-1 Protocol deviation codes and analysis sets

Category<br>Deviation code	Text description of deviation	Data exclusion
**Subjects are excluded from all *(safety)* analysis in case of these PDs:**		Exclude subject completely from all ***(safety)*** analysis sets
I01	*ICF not obtained*	*Yes*
S02	*Subject did not receive any study drug*	*Yes*
**Subjects are excluded from PK analysis in case of these PDs:**		Exclude subject from PK analysis set
I01	*ICF not obtained*	*Yes*
S02	*Subject did not receive any study drug*	*Yes*
**Subjects are excluded from PD analysis in case of these PDs:**		Exclude subject from PD analysis set
I01	*ICF not obtained*	*Yes*
S02	*Subject did not receive any study drug*	*Yes*

Category<br>Deviation code	Text description of deviation	Data exclusion
I03	*Diagnosis of PBC not demonstrated*	*Yes*
I07 (Part 1)	*Additional inclusion criterion for Part 1 not met*	*Yes*
I08 (Part 2)	*Additional inclusion criterion for Part 2 not met*	*Yes*
D01	*Withdrawal criteria met but subject not discontinued*	*Yes*
**Subjects are excluded from PK and PD analysis in case of these PDs:**		Exclude subject from PK and PD analysis sets
I01	*ICF not obtained*	*Yes*
S02	*Subject did not receive any study drug*	*Yes*
I03	*Diagnosis of PBC not demonstrated*	*Yes*
I07 (Part 1)	*Additional inclusion criterion for Part 1 not met*	*Yes*
I08 (Part 2)	*Additional inclusion criterion for Part 2 not met*	*Yes*
D01	*Withdrawal criteria met but subject not discontinued*	*Yes*

If updates to this table are needed, an amendment to RAP M3 needs to be implemented prior to DBL.

6 Statistical methods for Pharmacokinetic (PK) parameters

All subjects with at least one period of evaluable pharmacokinetic (PK) parameter data will be included in the pharmacokinetic data analysis.

6.1.1 Variables

In Part 1, the pharmacokinetic parameters include (but are not limited to) AUC, Cmax, Tmax for LJN452.

Plasma concentrations will be expressed in ng/mL. All concentrations below the limit of quantification or missing data will be labeled as such in the concentration data listings. Concentrations below the Limit of Quantification will be treated as zero in summary statistics for concentration data only. They will not be considered for calculation of PK parameters (with the exception of the pre-dose samples).

6.1.2 Descriptive analyses

Descriptive statistics of pharmacokinetic concentration and parameters will include mean (arithmetic and geometric), SD, CV (arithmetic and geometric), median, minimum and maximum. An exception to this is Tmax where median, minimum and maximum will be presented. Concentrations below LLOQ will be treated as zero in summary statistics and for PK parameter calculations. A geometric mean will not be reported if the dataset includes zero values. Pharmacokinetic parameters will be listed by treatment and subject.

At Day 1 and Day 28, AUC and Cmax will be summarized for each cohort along with 90% two-sided confidence intervals computed on the log-scale and back-transformed. All subjects with available PK parameters and no protocol deviation excluding them from the PK analysis set will be part of this computation.

LJN452 plasma concentration data will be listed by treatment, subject, and visit/sampling time point. Descriptive summary statistics will be provided by treatment and visit/sampling time point, including the frequency (n, %) of concentrations below the LLOQ and reported as zero. Additionally summary statistics may also be provided for the LJN452 plasma concentration data with a breakdown by UDCA stratum as appropriate.

6.1.3 Statistical model, assumptions and hypotheses

If 3 or more cohorts are included in Part 1 of the study, then dose-proportionality will be explored by fitting a power model to log(AUCtau) and log(Cmax). The model will specify a linear regression in log AUC (resp. log Cmax) regressed onto log dose level. This analysis will be done at Day 28 only.

Dose proportionality will be assessed on pharmacokinetic parameters AUCtau and Cmax on Day 28.

The pharmacokinetic parameters will be used to explore the relationship between dose and pharmacokinetic parameters using the power model on the log scale:

$$\ln(PKij) = \mu + \beta \ln(Dosej)$$

with β denoting the slope and Dosej denoting the total dose.

An estimator for the slope including the 90% confidence interval will be obtained based upon the log-transformed observations. This estimator and confidence interval will then be "back-transformed" to the original scale.

For exploratory information, PK dose proportionality will be considered across the whole dose range if the 90% confidence interval (β_L, β_U) for the slope βj is completely contained within a pre-specified critical region (b_L, b_U), where the two limits b_L, b_U are derived as follows:

$$b_L = 1 + \ln(\theta_L)/\ln(r) \quad \text{and} \quad b_U = 1 + \ln(\theta_U)/\ln(r)$$

with r=ratio of doses (highest dose/lowest dose), θ_L=0.8, θ_U=1.25 being the standard bioequivalence limits.

Graphical representation of dose proportionality will be explored.

6.1.4 Model checking procedures

The quality of the fit of the power model to assess dose-proportionality will be assessed by comparing the linear sub-model against a saturated model with a different mean level at each concentration. In case of evidence of lack-of-fit, alternative models will be explored.

6.1.5 Graphical presentation of results

Arithmetic mean (SD) plasma concentration data will be plotted across time, with separate line types for each population.

Overlaying individual plasma concentration-time profiles will be generated.

6.1.6 Pharmacokinetic / pharmacodynamic interactions

The relationship between PK (dose, concentration or PK parameter such as Cmax and AUC on Day 28) and key PD parameters (absolute and change from baseline to Day 28) including the primary endpoint GGT, the secondary efficacy/PD variables (PBC-40 total score, PBC-40 itch domain subscore, and the VAS scale PRO), [redacted] will be explored by graphic exhibition.

7 Statistical methods for Pharmacodynamic (PD) parameters

Analysis will be done for Part 1 and Part 2 separately unless specifically mentioned.

7.1 Primary objective

The primary efficacy objective is to evaluate the treatment effect of LJN452 over placebo in terms of reduction in cholestasis. The primary efficacy endpoint is the fold change in serum gamma-glutamyl transferase (GGT) from baseline to Day 28 and Day 85 for Part 1 and Part 2, respectively.

7.1.1 Variables

The primary efficacy endpoint is the fold change in serum GGT from baseline.

The serum GGT values at all time points will be logarithmically transformed prior to analysis. The rationale for the log transformation is to achieve more approximate normality and to be able to estimate a treatment effect on a ratio scale. Y=log(GGT+0.0001) will be considered only if there are many observations with GGT result as zero.

The change from baseline will be calculated as the difference between each of the log-transformed post-dose serum GGT values and the log-transformed baseline serum GGT value. This change from baseline will then be “back-transformed” to give a fold increase from baseline.

Baseline will be latest available pre-dose measurement.

7.1.2 Descriptive analyses

Unless stated otherwise, summary statistics for PD variables will include sample size (n), mean (arithmetic and geometric), SD, CV (arithmetic and geometric), median, minimum and maximum. A geometric mean will not be reported if the dataset includes zero values.

Summary statistics will be reported by treatment at each applicable time point for the original data and also the change from baseline or fold increases from baseline or percent change from baseline, as appropriate.

7.1.3 Statistical model, assumptions and hypotheses

Log-transformed changes from baseline for serum GGT will be analyzed by repeated measures analysis of covariance (ANCOVA) for each Part 1 and Part 2 separately. The model will include

treatment as a fixed effect, time as a repeated effect, the treatment by time interaction, the time by log-transformed baseline interaction and log-transformed baseline as a continuous covariate. The UDCA and pruritus stratification factor will also be included as a fixed effect in the ANCOVA model for the Part 2 analysis. A saturated covariance structure will be used for observations within the same subject. The model will be fitted using SAS Proc Mixed and the restricted maximum likelihood scoring method (reml). Two-sided 90% confidence intervals for the difference between each LJN452 dose and placebo will be evaluated for each visit. This will then be “back-transformed” to the original scale to give a fold change from baseline. Dose response may be assessed using appropriate contrasts. The analysis at the Day 28 (Visit 7) and day 85 (Visit 7) will constitute the primary analysis for Part 1 and Part 2, respectively.

The analysis will be done for each study cohort using pooled placebo data within Parts 1 and 2, respectively. No adjustment for multiplicity will be done.

Additionally an exploratory analysis on the combined data from Part 1 and Part 2 may be performed using a similar approach and the correlation between measurements taken in Part 1 and Part 2 from the same subject will be accounted for as appropriate.

Similar analysis as mentioned above can be performed for key LFT parameters.

In Part 2, there is the option of reducing the dose level for the individual subject. If for any subject the dose is reduced during the study, any data after the dose reduction will be set to missing. A repeated measures ANCOVA treating all measurements obtained post dose reduction as missing will be performed as well for Part 2 only.

7.1.4 Handling of missing values/censoring/discontinuations

All missing GGT values will be considered missing at random. No imputation or adjustment to the primary analysis will be done.

7.1.5 Model checking procedures

Residual plots will be produced and presented within the SAS OUTPUT listing. Residual plots will be checked and data transformations will be applied if necessary.

In the case of convergence issues of the primary model, alternative covariance structures will be considered and compared using standard fit statistics (AIC).

7.1.6 Graphical presentation of results

Arithmetic mean (±SD) of serum GGT levels and key LFT parameters, and fold change from baseline in serum GGT levels and key LFT parameters will be plotted across time.

Adjusted means and 90% CI from the repeated measures analysis of fold change from baseline in serum GGT levels and key LFT parameters will be plotted across time.

Placebo adjusted fold change from baseline and 90% CI will be plotted across time.

7.1.7 Supportive analyses

If it turns out that the primary analysis cannot be fitted because data cannot support a saturated model, a simpler covariance matrix such as the compound symmetric covariance structure may be fitted.

7.2 Secondary objectives

7.2.1 Variables

The secondary efficacy/PD variables are the PBC-40 total score, the PBC-40 itch domain subscore, and the VAS scale PRO as well as ALP (alkaline phosphatase).

The change from baseline will be calculated as the difference between each of the post-dose values and the baseline value.

Baseline will be latest available pre-dose measurement.

7.2.2 Descriptive analyses

See section 7.1.2.

7.2.3 Statistical model, assumptions and hypotheses

The difference between each LJN452 dose and placebo will be assessed by Wilcoxon rank sum test for change from baseline in total PBC score and itch subdomain subscore at each visit. A two-sided p-value and 90% CI for each treatment difference will be reported with no adjustment for multiplicity. In addition, summary statistics (mean, median, SD, minimum, maximum) will be reported at each applicable time point.

As an additional secondary end point, a 100mm visual analogue scale (VAS) will be used to assess the severity of patients' itch as well as loss of sleep. The score (distance from left) on the VAS will be recorded for both parameters by the patient marking with a line. The distance marked will be converted to a score between 0 and 10.

The change from baseline in VAS scores for itch and sleep loss will be analyzed by repeated measures analysis of covariance. The model will include treatment as a fixed effect, time as a repeated effect, the treatment by time interaction, the visit by baseline interaction and baseline as a continuous covariate. An unstructured covariance matrix will be used. Two-sided 90% confidence intervals for the difference between each LJN452 dose and placebo will be presented for each visit. The UDCA and pruritus stratification factor will also be included as a fixed effect in the model for the Part 2 analysis. Missing values will not be imputed.

ALP data will be analyzed using the same approach as described in Section 7.1.3.

Additionally, the number and percentage of subjects who have ALP<1.67xULN with >15% reduction from baseline and a normal total bilirubin level will be tabulated by part, treatment and visit.

7.2.4 Model checking procedures

See section 7.1.5.

7.2.5 Graphical presentation of results

Arithmetic mean (±SD) of the absolute levels and change from baseline in secondary variables will be plotted across time.

Box plots will be provided for change from baseline VAS and PBC scores.

Adjusted means and 90% CI from the repeated measures analysis of change from baseline in VAS scores for itch and sleep will be plotted across time.

Placebo adjusted change from baseline and 90% CI will be plotted across time.

[redacted]

8 Statistical methods for safety and tolerability data

8.1.1 Variables

Adverse events, vital signs (blood pressure, pulse rate, body temperature), ECG intervals, laboratory measurements, as well as subject demographics, baseline characteristics, and treatment information.

8.1.2 Descriptive analyses

Subject demographics and other baseline characteristics

All data for background and demographic variables will be listed by treatment group and subject. Summary statistics will be provided by treatment group.

Relevant medical history, current medical conditions, results of laboratory screens, drug tests and any other relevant information will be listed by treatment group and subject.

Treatments (study drug, rescue medication, other concomitant therapies, compliance)

Data for study drug administration (rescue medication) and concomitant therapies will be listed by treatment group and subject.

Vital signs

All vital signs data will be listed by part, cohort, treatment, subject, and visit/time and if ranges are available abnormalities (and relevant orthostatic changes) will be flagged. Summary statistics will be provided by part, cohort, treatment and visit/time.

ECG evaluations

All ECG data will be listed by part, cohort, treatment, subject and visit/time, abnormalities will be flagged. Summary statistics will be provided by part, cohort, treatment and visit/time.

Clinical laboratory evaluations

All laboratory data will be listed by part, cohort, treatment, subject, and visit/time and if normal ranges are available abnormalities will be flagged. A separate listing is provided presenting all parameters in a subject with any abnormal values. Summary statistics will be provided by part, cohort, treatment and visit/time.

The following special clinical laboratory evaluations will be listed and summarized by part, cohort, treatment and visit/time:

- Fasting lipid panel
- Alpha-fetoprotein (Listed only)
- Blood type (Listed only)

[redacted]

Adverse events

All information obtained on adverse events will be displayed by part, cohort, treatment and subject.

The number and percentage of subjects with adverse events will be tabulated by body system and preferred term with a breakdown by treatment. A subject with multiple adverse events within a body system is only counted once towards the total of this body system and treatment.

Pregnancy test

All pregnancy test results for women will be listed by part, cohort, subject and visit

Immunogenicity

Not applicable.

8.1.3 Graphical presentation of results

Boxplots to visualize trends in longitudinal safety data (vitals, ECG, lab parameter) will be created.

[redacted]

[redacted]

[redacted]
