# Supplementary material for: Farnesoid X receptor agonist tropifexor attenuates cholestasis in a randomised trial in patients with primary biliary cholangitis
Source: JHEP Rep. 2022 Jul 21;4(11):100544. doi: 10.1016/j.jhepr.2022.100544 (PMC9576902; doi:10.1016/j.jhepr.2022.100544)
Supplement: Multimedia component 3 [file mmc3.pdf]

NOVARTIS

Novartis Institutes for BioMedical Research

Tropifexor (LJN452)

Clinical Trial Protocol CLJN452X2201 / NCT02516605

A multi-part, randomized, double-blind, placebo-controlled study to assess the safety, tolerability and efficacy of tropifexor (LJN452) in patients with Primary Biliary Cholangitis

Document type:	Amended Protocol Version
EUDRACT number:	2015-001590-41
Version number:	v06 (Clean)
Development phase:	II
Release date:	27-Nov-2017

Property of Novartis
Confidential
May not be used, divulged, published or otherwise disclosed
without the consent of Novartis

[redacted]

Site Operations Manual (SOM)

A Site Operations Manual (SOM) accompanies this protocol, providing the operational details for study conduct.

Notification of serious adverse events

Refer to Section 9.2 of the protocol for definitions and reporting requirements for Serious Adverse Events (within 24 hours after awareness of the SAE to the local Novartis Chief Medical Office and Patient Safety Department and notify the Clinical Trial Leader).

Contact information is listed in the Site Operations Manual.

[redacted]

Table of contents

Site Operations Manual (SOM) ... 2
Notification of serious adverse events ... 2
Table of contents ... 3
List of tables ... 6
List of figures ... 6
List of abbreviations ... 7
Pharmacokinetic definitions and symbols ... 10
Amendment 6 (November 2017) ... 11
Amendment 5 (June 2017) ... 12
Amendment 4 (July 2016) ... 16
Amendment 3 (March 2016) ... 18
Amendment 2 (November 2015) ... 19
Amendment 1 (September 2015) ... 20
Protocol synopsis ... 21
1 Introduction ... 25
1.1 Background ... 25
1.1.1 Relevant data summary ... 25
1.2 Study purpose ... 30
2 Study objectives ... 31
2.1 Primary objectives ... 31
2.2 Secondary objectives ... 31
[redacted] 32
3 Investigational plan ... 32
3.1 Study design ... 32
3.2 Rationale of study design ... 36
[redacted] 37
3.4 Rationale for choice of comparator ... 38
3.5 Purpose and timing of interim analyses/design adaptations ... 39
3.6 Risks and benefits ... 40
4 Population ... 40
4.1 Inclusion criteria for Parts 1 and 2 ... 40
4.1.1 Additional Inclusion Criteria for Part 1 ... 41
4.1.2 Additional Inclusion Criteria for Part 2 ... 41
4.2 Exclusion criteria for Parts 1 and 2 ... 41
5 Restrictions for Study Subjects ... 43

[redacted]

5.1 Contraception requirements ... 43
5.2 Prohibited treatment ... 44
5.3 Dietary restrictions and smoking ... 44
5.4 General restrictions ... 44
6 Treatment ... 45
6.1 Study treatment ... 45
6.1.1 Investigational treatment ... 45
6.2 Treatment arms ... 45
6.2.1 Permitted dose adjustments and interruptions of study treatment ... 46
6.3 Treatment assignment ... 47
6.4 Treatment blinding ... 48
6.5 Emergency breaking of assigned treatment code ... 49
6.6 Treatment exposure and compliance ... 50
6.7 Recommended treatment of adverse events ... 50
6.8 Rescue medication ... 50
6.9 Concomitant treatment ... 50
7 Discontinuation and study completion ... 50
7.1 Discontinuation of study treatment ... 50
7.2 Study completion and post-study treatment ... 51
7.2.1 Lost to follow-up ... 52
7.3 Withdrawal of informed consent ... 52
7.4 Study Stopping rules ... 52
7.5 Early study termination ... 54
8 Procedures and assessments ... 54
8.1 Informed consent procedures ... 57
8.2 Subject demographics/other baseline characteristics ... 58
8.3 Efficacy / Pharmacodynamics ... 58
8.3.1 Liver Function Tests ... 59
8.3.2 Patient-Reported Outcome ... 59
8.4 Safety ... 59
8.4.1 Physical examination ... 59
8.4.2 Vital signs ... 59
8.4.3 Height and weight ... 60
8.4.4 Laboratory evaluations ... 60
8.4.5 Electrocardiogram (ECG) ... 60
8.4.6 Pregnancy ... 61

[redacted]

8.5 Pharmacokinetics 61
8.5.1 PK Blood collection and processing 61
8.5.2 Pharmacokinetic analytical method(s) 61
[redacted] 61
[redacted] 61
9 Safety monitoring 64
9.1 Adverse events 64
9.2 Serious adverse event reporting 66
9.2.1 Definition of SAE 66
9.2.2 SAE reporting 67
9.3 Liver safety monitoring 68
9.4 Renal safety monitoring 68
9.5 Pregnancy reporting 68
9.6 Early phase safety monitoring 69
10 Data review and database management 69
10.1 Site monitoring 69
10.2 Data collection 70
10.3 Data Monitoring Committee 71
10.4 Adjudication Committee 71
11 Data analysis 72
11.1 Analysis sets 72
11.2 Subject demographics and other baseline characteristics 72
11.3 Treatments (study drug, rescue medication, other concomitant therapies, compliance) 72
11.4 Analysis of the primary variable(s) 72
11.4.1 Variable(s) 72
11.4.2 Statistical model, hypothesis, and method of analysis 73
11.4.3 Handling of missing values/censoring/discontinuations 73
11.4.4 Supportive analyses 73
11.5 Analysis of secondary [redacted] variables 73
11.5.1 Efficacy / Pharmacodynamics 73
11.5.2 Safety 74
11.5.3 Pharmacokinetics 75
11.5.4 Pharmacokinetic / pharmacodynamic interactions 76
[redacted] 76
11.6 Sample size calculation 77
11.7 Power for analysis of key secondary variables 77

[redacted]

11.8 Interim analyses 77
11.9 Interim analyses on locked data 78
12 Ethical considerations 79
12.1 Regulatory and ethical compliance 79
12.2 Responsibilities of the investigator and IRB/IEC 79
12.3 Publication of study protocol and results 79
13 Protocol adherence 80
13.1 Protocol Amendments 80
14 References 81
15 Appendix 1: Liver and Renal events definitions and required actions 83
16 Appendix 2: Pruritus event definitions and required actions 90

List of tables

[redacted] [redacted] 29
Table 6-1 Part 2 Permitted dose reductions 47
Table 8-1 Part 1 Assessment schedule 55
Table 8-2 Part 2 Assessment schedule 57
Table 15-1 Liver Triggers and Events: definition and Follow-up Requirements 84
Table 15-2 Renal events: definition and follow up requirements 89
Table 16-1 Pruritus event definitions and required actions 90

List of figures

[redacted] [redacted] 26
Figure 3-1 Study design 33
Figure 3-2 Study design Part 1 34
Figure 3-3 Study design Part 2 35

[redacted]

List of abbreviations

AE	adverse event
ALT	alanine aminotransferase (SGPT)
ALP	alkaline phosphatase
ANCOVA	analysis of covariance
ANIT	alpha-naphthyl -isothiocyanate
(A)PPT	(activated) partial thromboplastin time
AST	aspartate aminotransferase (SGOT)
BMI	Body Mass Index
BSEP	bile salt export pump
BUN	blood urea nitrogen
CD-ROM	compact disc – read only memory
CFR	Code of Federal Regulation
CK	creatinine kinase
CRF	Case Report/Record Form (paper or electronic)
CRO	Contract Research Organization
CSR	Clinical Study Report
CTC	Common Toxicity Criteria
CTCAE	Common Terminology Criteria for Adverse Events
CTL	Clinical Trial Leader
CTT	Clinical Trial Team
CV	coefficient of variation
DMC	Data Monitoring Committee
EC	Ethics committee
ECG	Electrocardiogram
EDC	Electronic Data Capture
[redacted]	[redacted]
FGF15	Fibroblast growth factor 15
FPFV	First patient first visit
FXR	Farnesyl X receptor
GCP	Good Clinical Practice

[redacted]

GLP	Good laboratory practice
GGT	Gamma-glutamyl transferase
h	hour
HDL	high-density lipoprotein
ICH	International Conference on Harmonization of Technical Requirements for Registration of Pharmaceuticals for Human Use
IEC	Independent Ethics Committee
INR	International Normalized Ratio
IN	Investigator Notification
IRB	Institutional Review Board
IRT	Interactive Response Technology
LFT	Liver function test
LDH	lactate dehydrogenase
LDL	low-density lipoprotein
LLOQ	lower limit of quantification
MedDRA	Medical dictionary for regulatory activities
MELD	Model for End Stage Liver Disease
mg	milligram(s)
ml	milliliter(s)
[redacted]	[redacted]
NASH	Non alcoholic steatohepatitis
NOAEL	no-observed-adverse-effect-level
NSAID	Nonsteroidal Antiinflammatory drugs
PBC	Primary Biliary Cholangitis (formerly Primary Biliary Cirrhosis)
PBC-40	a disease specific quality of life measure for PBC
PD	pharmacodynamic(s)
PK	pharmacokinetic(s)
PoC	Proof of Concept
PRO	Patient reported outcomes
PT	prothrombin time
RBC	red blood cell(s)
SAE	serious adverse event

[redacted]

sCR	serum creatinine
SD	standard deviation
SGOT	serum glutamic oxaloacetic transaminase (same as AST)
SGPT	serum glutamic pyruvic transaminase (same as ALT)
SHP	small heterodimer partner
SOM	site operations manual
SUSAR	Suspected Unexpected Serious Adverse Reactions
TBL	total bilirubin
TIA	transient ischemic attack
UDCA	ursodeoxycholic acid
ULN	upper limit of normal
ULOQ	upper limit of quantification
VAS	Visual Analog Score
WBC	white blood cell(s)

[redacted]

Pharmacokinetic definitions and symbols

AUC0-t	The area under the plasma concentration-time curve from time zero to time ‘t’ where t is a defined time point after administration [mass x time / volume]
AUCtau	The area under the plasma concentration-time curve from time zero to the end of the dosing interval tau [mass x time / volume]
[redacted]	[redacted]
Cav,ss	The average steady state plasma concentration during multiple dosing
Cmax	The observed maximum plasma concentration following drug administration [mass / volume]
Cmax,ss	The observed maximum plasma (or serum or blood) concentration following drug administration at steady state [mass / volume]
Cmin,ss	The lowest plasma concentration observed during a dosing interval at steady state [mass / volume]
CL/F	The apparent systemic (or total body) clearance from plasma following extravascular administration [volume / time]
Tmax	The time to reach the maximum concentration after drug administration [time]
Vz/F	The apparent volume of distribution during the terminal elimination phase following extravascular administration [volume]

[redacted]

Amendment 6 (November 2017)

Amendment rationale

The purpose of this amendment is to address comments [redacted] in response to protocol amendment 5. In addition, the blood volume was updated to enable the collection of additional samples for Vitamin D, [redacted] and PK back-up samples.

Changes to the protocol

[redacted]

- The blood volume for Parts 1 and 2 were updated to 490 mL and 450 mL, respectively, in Section 3.6 Risks and Benefits. This enables sample collection for Vitamin D, [redacted] and back up sample collection for PK samples.
- The inclusion and exclusion criteria in Section 4 were updated to exclude patients with:
    - significant hepatic impairment (Child-Pugh B and C),
    - serum creatinine above the normal range,
    - history of cirrhosis with complications
    - limit the population to PBC patients with mild/moderate disease by adapting the inclusion/exclusion criteria for ALT/AST, INR, and total bilirubin.
- Section 7.1 Discontinuation of study treatment was updated to state that if a patient has a hepatic decompensation event, study treatment must be stopped.
- Table 8-1 Part 1 Assessment schedule was updated to remove '-30m' from the pre-dose assessment time for Day 28, which was erroneously left in when protocol amendment 5 was made.
- Table 15-1 Liver Triggers and Events: definition and Follow-up Requirements has been updated to increase the frequency of repeat tests following re-starting study drug and to clarify that if there is an elevation of liver biochemistries after re-starting the study treatment should be discontinued and the patient followed-up.
- Table 15-2 Renal events: definition and follow up requirements has been updated to require discontinuation of study drug if there is a confirmed doubling of serum creatinine or serum creatinine ≥1.5 mg/dL
- Editorial updates and corrections to typographical errors have also been made.

[redacted]

Amendment 5 (June 2017)

Amendment rationale

The purpose of this amendment is primarily to enable higher dose(s) in Parts 1 and 2 of the study, [redacted] and to update the Part 2 study design to allow 12 weeks of treatment in patients with Primary Biliary Cholangitis (PBC), to provide longer-term safety, tolerability and efficacy data.

Specific changes to Part 2 include:

- increase the treatment duration from 4 weeks to 12 weeks,
- broaden the population to include patients both taking and not taking UDCA (ursodeoxycholic acid), to be more representative of the PBC patient population,
- assess two dose levels (plus placebo), to gain an insight into the dose-effect relationship,
- increase patient numbers and alter randomization ratio to randomize more patients to receive active treatment compared to placebo, for collection of more safety data in this population,
- reduce the burden for the patient by only collecting sparse PK samples.

[redacted]

Changes also have been made to enable the staggered database lock of the study, so the database can be cleaned and locked in stages such as at completion of Part 1, or completion of a set of cohorts in Part 1 as well as the final database lock at the end of the study. Once the database has been locked, the locked data may be communicated beyond Novartis to groups including, but not limited to individuals treating the study's subjects, Health Authorities and shared on clinical registries, as a means of greater transparency.

Changes to the protocol

- Title updated with the International Nonproprietary Name (INN) of tropifexor.
- Objectives (Protocol synopsis and Section 2 Study objectives) updated to reflect the objectives relating to the amended Part 2.

[redacted]

- Study Design (Protocol synopsis and Section 3 Investigational Plan) updated to reflect the changes relating to the amended Part 2, including updating the design, the rationale for the study design and dose/regimen and associated interim analysis (Section 3.5 and Section 11.8). Section 1.2 Study purpose was also updated accordingly.
- The amendment includes changes to enable the staggered locking of the database following completion of Part 1, or completion of Cohorts in Part 1 and the sharing of the locked data. Various parts of the protocol have been updated as a result of this, including: Section 3.2 Rationale of study design, Section 3.5 Purpose and timing of interim analyses/design adaptations, Section 6.4 Treatment blinding, Section 10.2 Data collection, Section 11.8 Interim analysis and Section 11.9 Interim analyses on locked data.
- The total blood volume collected during the study was updated in Section 3.6 to reflect the recalculated and reduced volume for Part 1 and the new Part 2 volume.
- Section 4 Population: has been updated to increase the sample size in Part 2, mentioning that the final sample size will be dependent on the interim analysis. The same section in the Protocol synopsis has also been updated with this information and to reflect the changes made to the eligibility criteria (see details below), together with the use of stratification in Part 2.
- Section 4.1.2 Additional Inclusion Criteria for Part 2: has been updated to reflect that the population for Part 2 will now include patients taking a stable dose of UDCA as well as those who are not taking UDCA.
- Several updates have been made to Section 4.2 Exclusion criteria to enhance patient safety, including to increase the time women of child-bearing potential need to use highly effective methods of contraception before and after treatment; to clarify the eligibility of those with previous viral hepatitis; to simplify the exclusion criteria related to hepatic decompensation to focus on cirrhosis with complications; to exclude patients that have laboratory results at screening that, if they occurred post-dose, would require them to discontinue study drug, to exclude the use of immunosuppressive drugs and FXR agonist obeticholic acid, and to exclude patients with a history of immunodeficiency diseases, including those with a positive HIV test result. Patients who have previously participated in Part 1 of the study are excluded from re-enrolling in Part 2 if they have received study medication within three months of randomization.
- Section 5.2 Prohibited treatment: was updated with the addition of obeticholic acid, immunosuppressive drugs and investigational drugs as a prohibited treatment.
- Section 5.3 was updated to lessen the restrictions on patients. [redacted] Fasting requirements in Part 2 have been changed to be no fluid for 30 min post dose and no food until 60 min post dose. The restriction on alcohol intake has been reduced from 12 hours to 8 hours prior to dosing or study visits.

[redacted]

- Section 6.1.1 Investigational treatment: An additional dose strength of 0.1 mg has been added In Part 1, a manual randomization process is used to provide randomization numbers upon confirmation of eligibility at the Baseline visit. In Part 2, an Interactive Response Technology (IRT) system will be used for management of randomization. Section 6.1.1 Investigational treatment, Section 6.3 Treatment assignment, Section 6.5 Emergency breaking of assigned treatment code and Section 10.2 Data collection have been updated to reflect these differences.
- Updates have been made to reflect the doses administered in Cohorts 1-3 in the Protocol synopsis and Section 6.2 Treatment arms.
- Treatment interruptions are permitted in Part 1 in response to elevations in AST, ALT or pruritus. Dose adjustments and treatment interruptions are permitted in Part 2 in response to elevations in AST, ALT or pruritus (interruption only). Section 6.2.1 Permitted dose adjustments and interruptions of study treatment and Section 7.1 Discontinuation of study treatment have been updated to reflect this. Table 15-1 and Table 6-1 provide the details for the follow up actions and when dose interruption or dose reductions are appropriate.
- Section 6.4 Treatment blinding has been updated to reflect the use of a double dummy design in Part 2 to maintain the blind across the two dose arms of LJN452.
- The use of paper diaries has been noted as an aide to treatment exposure and compliance in Section 6.6. The diaries will also be used to collect details of any concomitant medication the patient takes in response to an adverse event of itch. It has been noted the Investigator should review the use of the diary with the patient.
- Section 6.8 Rescue medication has been updated to flag that patients with a high pruritus score at baseline or a history of pruritus should have a suitable supply of anti-pruritic therapy available at home.
- Section 7.1 Discontinuation of study treatment has been revised to simplify the wording. Liver and renal related stopping criteria have been clarified and moved from Section 7.1 to Table 15-1 and Table 15-2 in Appendix 1 (adapted from previous Table 9-2 and Table 9-3 in Liver and Renal safety monitoring sections). The rationale for this change is to have all actions required following elevation of liver or renal markers in one place, as opposed to in multiple locations (e.g., stopping criteria, dose adjustment criteria and liver/renal safety monitoring) and to move to the back of the protocol for ease of reference by site staff.
- Stopping criteria have been revised so that a CTCAE Grade 3 that is suspected to be related or a CTCAE Grade 4 (regardless of the relationship to study drug) have been identified as requirements to withdraw study medication, with the exception of pruritus, liver or renal parameters, which have their own stopping criteria outlined in Appendix 1 and Appendix 2 .

[redacted]

- Section 7.2 and Section 9.2.2 have been updated so that SAEs are collected from signing the informed consent until 30 days after the end of study treatment. Hence, the trigger for the safety follow up call is now 30 days after last treatment, rather than last visit, in line with revised Novartis requirements. Most patients will have a visit in this timeframe, so will not require a follow up safety call.
- Section 7.4 Study stopping rules has been updated to reflect updates made to individual stopping criteria. It now specifies that the cohort stopping rules only apply to Part 1 (as dose escalation section of the study), [redacted] Details have been added to specify that for Part 2 the DMC will review safety data on a regular basis.
- Section 8 has been updated with a Part 2 specific assessment schedule. In Part 1, the 30 min pre-dose window was removed to make scheduling more practical. Medical history was added to the table and clarity was added to define which results remained as source data, as these were previously missing in error.
- In Protocol Amendment 4, the option for the Investigator to delegate certain visit assessments to be performed at an alternative location was introduced. This has not been used and will not be available for Part 2, so references to this have been removed from Section 8 and throughout the protocol.
- Section 8.4.4 Laboratory evaluations have been updated to add magnesium, phosphate and vitamin D as well as to specify all assessments performed as part of the clinical chemistry assessment (including fasting lipid panel).
- Details of the pregnancy tests have been outlined in Section 8.4.6 Pregnancy.

[redacted]

- Section 9.1 has been updated to remove collection of 'outcome' in the CRF as this is not collected in the CRF and clarification has been made as to when follow up of AEs continues until (end of study).
- Section 9.3 Liver safety monitoring and Section 9.4 Renal safety monitoring have been streamlined and the information in Table 9-1, Table 9-2 and Table 9-3 moved and refined to be presented in Table 15-1 and Table 15-2 in Appendix 1, so easier to find. Table 15-1 includes study drug discontinuation (stopping criteria), liver safety monitoring (from Section 9) and new down titration options. Table 15-2 includes study drug discontinuation (stopping criteria) and renal safety monitoring (from Section 9). Since urinary protein-creatinine ratio varies by gender and age, one criteria has changed from 'Protein-creatinine ratio (PCR) ≥ 150 mg/g or ≥ 15 mg/mmol' to Protein-creatinine ratio (PCR) ≥ ULN'.
- Section 10.3 Data Monitoring Committee has been updated to define the role the DMC will play in oversight of Part 2 of the study.
- Data analysis has been updated to reflect the study design changes to Part 2 in Section 11 and in the Protocol synopsis.

[redacted]

- Creation of a table to outline the definitions of CTCAE Grades for Pruritus and the required actions, which include an option for study drug interruption of up to a week with CTCAE Grade 3 pruritus.
- Section 14 References were updated with changes to references made in the text.
- Editorial updates were made.

Amendment 4 (July 2016)

Amendment rationale

The purpose of this amendment is to address feedback [redacted] on Amendment 3. Changes have also been made to the protocol to reduce the burden on the patients participating in the study.

Additional changes have been made including clarification and slight modification of eligibility criteria, updated text describing the statistical analysis for clarification, addition of a higher dosage form, and minor editorial updates.

The study is currently ongoing, with three patients dosed. It is not anticipated that the changes in the amendment will influence the study population or results of the study.

Changes to the protocol

- Section 3.1 'Study design' has been updated to reflect the changes made to the timing of assessments on Days 1 and 28, that study medication for the full 28 days will be dispensed on Day 1 and that there is an option for Investigators to delegate responsibility for certain visits to be performed at an alternative location.
- Figure 3-1 updated to remove day details for screening and baseline visits, as are a duplicate of the assessment schedule.
- Updates have been made to [redacted] Section 3.4 'Rationale for choice of comparator' to reflect the recent approval of FXR agonist obeticholic acid.
- Inclusion criteria 4 (Protocol synopsis and Section 4.1) has been updated to increase the BMI range from 18 – 35 kg/m^2 to 18 – 40 kg/m^2.
- Additional inclusion criteria for Part 1 (Section 4.1.1) has been expanded to include patients who have been taking UCDA for at least 6 months (with no change in dose for ≥ 3 months), provided they have reached maximal response to UCDA with a plateau in alkaline phosphatase levels. Whilst the initial requirement for 1 year of treatment is based on the widely held criteria to define UDCA response, it is clear that those that are non-responsive can be identified sooner (Carbone et al 2013). The minor change to the inclusion criteria will ensure that UDCA non-responder population will have early access to LJN452. Phenotypically these patients will be virtually identical to those recruited under previous inclusion criteria.
- Exclusion criteria 3 and 4 have been clarified in Section 4.2.
- Section 5.2 'Prohibited treatment' has been updated to align with other studies with LJN452.

[redacted]

- Section 5.3 'On Day 1, no breakfast will be provided' has been removed as is an unnecessary restriction.
- The additional dose form of 0.03 mg has been added in Section 6.1.1 'Investigational treatment'. The higher strength dose form will simplify dosing if higher dose levels are reached through planned dose escalation.
- 'Liver safety monitoring' (Section 9.3) and 'Renal safety monitoring' (Section 9.4) sections have been referred to in Section 7.1 'Discontinuation of study treatment'.
- Addition of stopping rule for total bilirubin when elevated at baseline has been added to Section 7.1. 'Discontinuation of study treatment'.
- Section 7.4 'Study stopping rules' has two updates:
  [redacted]
  - Clarified stopping rule for when bilirubin is elevated at baseline.
- Section 8 'Procedures and assessments' and Table 8-1 'Assessment schedule' have been updated as follows:
  - Visit windows for Screening and Baseline visits have been adjusted to avoid patients having to attend an additional visit to be re-screened to take account of when recruitment of patients 'pauses' for data review and dose escalation evaluation in-between cohorts. All subjects will have their eligibility reconfirmed at the baseline visit which will occur within 14 days prior to dosing.
  - Moved Day 1 pulse and blood pressure assessment timepoints from 6 hour to 4 hour post dose
  - Make later PK [redacted] sample collection timepoints optional on Days 1 and 28
  - [redacted] Additional pregnancy tests have been added to adhere to Novartis guidance on monthly pregnancy testing for women of childbearing potential that are able to be included in the study if they are taking highly effective contraception. Pregnancy testing has been added at Day 1, Day 28 and Day 56.
  - Provide the option for the Investigator to delegate certain visit assessments to be performed at an alternative location, which will have the required ethical approvals in place and be documented on the delegation of duties log
  - Add 'Drug dispensing' activity, to be performed on Day 1 (rather than at each visit)

[redacted]

- Section 9.3 'Liver safety monitoring' has been aligned with individual stopping criteria in Section 7.1, with guidance added for action in the event of initially elevated baseline values of ALT, AST, ALP or Bilirubin, plus provided more guidance on timeframes required for repeat assessments

[redacted]

- Table 9-3 'Specific Renal Alert Criteria and Actions' has been updated to provide greater guidance on repeat test timeframe and requirement to withhold study drug until further evaluation if cannot be performed in this time-frame.
- Updates have been made to provide greater clarity on the planned data analysis. Changes have been made in the synopsis and in Section 11 'Data analysis'.
- Reference added to Section 14 'References'.

Changes to specific sections of the protocol are shown in the track changes version of the protocol using strike through red font for deletions and red underlined for insertions.

A copy of this amended protocol will be sent to the Institutional Review Board (IRBs)/Independent Ethics Committee (IECs) and Health Authorities.

The changes described in this amended protocol require IRB/IEC approval prior to implementation.

Amendment 3 (March 2016)

Amendment rationale

The purpose of this amendment is to include emerging data [redacted] As a result of the new data, a separate renal function exclusion criteria were added. In addition other minor changes, including the inclusion of ALP isozyme analysis, a patient dosing diary to monitor compliance and typographical corrections have been made to the protocol.

Changes to the protocol

- Section 1.1.1 has been updated to include emerging data from [redacted]

[redacted]

- The total blood volume collected during the study was updated in Section 3.6
- The description of highly effective contraception in the Exclusion Criteria was updated
- The Exclusion Criteria have been updated to include a separate criteria related to renal function
- Individual treatment discontinuation rules (Section 7.1) and study stopping rules (Section 7.4) have been updated
- Section 8.1 was modified to remove the use of a legal representative giving consent on behalf of a patient unable to give consent themselves
- A patient diary for compliance monitoring has been added to Section 8.3.2

[redacted]

- Clarifying text was added in Section 11.4.1, Section 11.4.2 and Section 11.5.1
- Primary Biliary Cirrhosis has been changed to Primary Biliary Cholangitis throughout the protocol

[redacted]

Changes to specific sections of the protocol are shown in the track changes version of the protocol using strike through red font for deletions and red underlined for insertions.

A copy of this amended protocol will be sent to the Institutional Review Board (IRBs)/Independent Ethics Committee (IECs) and Health Authorities.

The changes described in this amended protocol require IRB/IEC approval prior to implementation.

The changes herein affect the Informed Consent. Sites are required to update and submit for approval a revised Informed Consent that takes into account the changes described in this protocol amendment.

Amendment 2 (November 2015)

Amendment rationale

During the creating of Version No. 01 of the protocol, pregnancy testing was inadvertently removed from the Assessment Schedule. The purpose of this amendment is to re-add pregnancy testing into the protocol.

Changes to the protocol

- Pregnancy testing was re-added to the Assessment Schedule

[redacted]

- Text was added to Section 8.4.1

Changes to specific sections of the protocol are shown in the track changes version of the protocol using strike through red font for deletions and red underlined for insertions.

A copy of this amended protocol will be sent to the Institutional Review Board (IRBs)/Independent Ethics Committee (IECs) and Health Authorities.

This amendment is required for patient safety (i.e., necessary to eliminate immediate hazards to the trial subjects ICH GCP 3.3.8). Therefore it will be implemented prior to IRB/IEC approval, but will be sent for approval as well.

[redacted]

Amendment 1 (September 2015)

Amendment rationale

The purpose of this amendment is to address comments from the MHRA.

Changes to the protocol

- Text has been removed from Section 3.1 to remove the option of placebo patients being re-enrolled in future Cohorts in Part 1.
- [redacted] Section 6.2 have been updated, to include text clarifying the maximum dose to be administered in Part 2.
- Information has been added to the Additional Inclusion Criteria for Part 2 in Section 4.1.2, to provide specific information on patients not currently taking UDCA.

[redacted]

Changes to specific sections of the protocol are shown in the track changes version of the protocol using strike through red font for deletions and red underlined for insertions.

A copy of this amended protocol will be sent to the Institutional Review Board (IRBs)/Independent Ethics Committee (IECs) and Health Authorities.

The changes described in this amended protocol are non-substantial and do not require IRB/IEC approval prior to implementation.

[redacted]

Protocol synopsis

**Protocol number**	CLJN452X2201
**Title**	A multi-part, randomized, double-blind, placebo-controlled study to assess the safety, tolerability and efficacy of tropifexor (LJN452) in patients with Primary Biliary Cholangitis
**Brief title**	Study of safety, tolerability and efficacy of LJN452 in patients with Primary Biliary Cholangitis
**Sponsor and Clinical Phase**	Novartis<br>Phase 2
**Intervention type**	Drug
**Study type**	Interventional
**Purpose and rationale**	The purpose of this study to assess the safety, tolerability and efficacy of LJN452 in patients with Primary Biliary Cholangitis
**Primary Objective(s)**	• To determine the safety and tolerability of daily dosing of LJN452 in patients with Primary Biliary Cholangitis<br>• To determine the effect of LJN452 on cholestatic markers in patients with Primary Biliary Cholangitis. More specifically, in Part 2 to determine the dose-response relationship of LJN452 on GGT following 12 weeks of treatment
**Secondary Objectives**	• To determine the change in the itch domain of PBC40 questionnaire<br>• To evaluate the change in overall disease specific quality of life<br>• To evaluate the change in itch based on 100 mm visual analog score (VAS)<br>• To evaluate the pharmacokinetics (PK) of LJN452 in patients with PBC<br>• Part 2: To determine the dose-response relationship of LJN452 on ALP in patients with PBC following 12 weeks of treatment
**Study design**	This is a randomized, double-blind, placebo controlled, multi-part study to assess safety, tolerability and efficacy of LJN452 in patients with Primary Biliary Cholangitis (PBC).<br>Part 1 uses an escalating multiple dose design in PBC patients with incomplete biochemical response to, but still taking, ursodeoxycholic acid (UDCA).<br>Part 2 is a parallel-group, 12-week study to assess the safety, tolerability and efficacy of two doses of LJN452 as compared to placebo in PBC patients with an incomplete biochemical response to, but still taking UDCA or in those not currently taking UDCA.<br>The two LJN452 doses for Part 2 will be selected based on safety, tolerability and efficacy data from Part 1.<br>Once approximately 40 patients from Part 2 have completed 8 weeks of treatment, an interim analysis will be conducted to re-assess the sample size. Recruitment will continue while the interim analysis is performed.

[redacted]

**Population**	The study population will comprise male or female patients with PBC aged 18 and above.<br>In Part 1 of the study a minimum of 30 and maximum of approximately 75 PBC patients with an incomplete biochemical response, but still currently taking UDCA will be randomized to LJN452 or placebo.<br>In Part 2 approximately 88 PBC patients will be randomized to either Dose level 1 or Dose level 2 of LJN452, or matching placebo in a 3:3:2 ratio.<br>The maximum dose to be used in the study is 0.18 mg.<br>Patients with an incomplete biochemical response to, but still taking, UDCA and those not currently taking UDCA will be stratified across treatment arms.<br>Severity of pruritus will also be used to stratify patient across treatment arms.<br>Final sample size for Part 2 will be informed by the interim analysis.
**Inclusion criteria**	• Age ≥ 18 years<br>• Diagnosis of PBC as demonstrated by the presence of ≥ 2 of the following 3 diagnostic criteria:<br>◦ History of Alkaline Phosphatase elevated above upper limit of normal for at least 6 months<br>◦ Positive antimitochondrial antibodies (AMA) titer or if AMA negative or in low titer (<1:80) PBC specific antibodies (anti-GP210 and/or anti-SP100 and/or antibodies against the major M2 components (PDC-E2, 2-oxo-glutaric acid dehydrogenase complex))<br>◦ Previous liver biopsy consistent with PBC<br>• At least 1 of the following markers of disease severity:<br>◦ ALP ≥ 1.67× upper limit of normal (ULN)<br>◦ Total bilirubin > ULN but < 1.5 × ULN<br>In addition, patients must meet the following biochemical criteria at enrollment<br>◦ ALT/AST ≤5 × ULN<br>◦ Total bilirubin ≤1.5 × ULN<br>◦ INR ≤ ULN<br>• Subjects must weigh at least 40 kg to participate in the study, and must have a body mass index (BMI) within the range of 18 - 40 kg/m^2. BMI = Body weight (kg) / [Height (m)]^2.<br>• Able to communicate well with the investigator, to understand and comply with the requirements of the study.
**Exclusion criteria**	• Women of child-bearing potential, defined as all women physiologically capable of becoming pregnant, unless they are using highly effective methods of contraception for 30 days before randomization, during dosing and for 30 days following the end of treatment.<br>• Presence of other concomitant liver diseases.<br>• Cirrhosis with complications, including history or presence of:<br>• Variceal bleed<br>• Uncontrolled ascites<br>• Encephalopathy<br>• Spontaneous bacterial peritonitis

[redacted]

	• Significant hepatic impairment as defined by Child-Pugh classification of B or C, history of liver transplantation, current placement on a liver transplant list or current Model for End Stage Liver Disease (MELD) score ≥15.<br>• History of conditions that may cause increases in ALP (e.g., Paget's disease).<br>• Use of investigational drugs, or immunosuppressive drugs at the time of enrollment, or within 5 half-lives, or 30 days of randomization, whichever is longer; or longer if required by local regulations. Use of high dose oral steroids to treat co-morbid conditions (e.g., airways disease) will be allowed but must be properly documented as such in concomitant medications.<br>• Currently taking obeticholic acid or have taken obeticholic acid within 30 days of randomization<br>• Previous participation in CLJN452X2201 and received study medication within three months of randomization (or longer if required by local regulations).
**Investigational and reference therapy**	• Part 1:<br>• Cohort 1 LJN452 0.03 mg or placebo once daily for 28 days,<br>• Cohort 2 LJN452 0.06 mg or placebo once daily for 28 days,<br>• Cohort 3 LJN452 0.09 mg or placebo once daily for 28 days<br>• Cohorts 4-5 LJN452 TBD mg or placebo once daily for 28 days<br>• Part 2: Two dose levels of LJN452 (doses TBD from Part 1 data), or placebo once daily for 12 weeks. Dose adjustments will be permitted.
**Efficacy/PD assessments**	• Liver function tests, specifically GGT, ALP and bilirubin<br>• PBC40 for quality of life and itch<br>• VAS for itch and sleep
**Safety assessments**	• Physical exam<br>• Vital signs<br>• Laboratory evaluations<br>• ECG
[redacted]	

[redacted]

**Data analysis**	Part 1: The primary efficacy endpoint (Serum GGT measured at Day 28) will be analyzed by repeated measures analysis of covariance. The analysis will take place on the log-transformed scale and encompass all available GGT measurements over time. The model will use a saturated within-subject covariance structure and a separate fixed effect for treatment, visit, treatment*visit interaction and log baseline as well as log(baseline)* visit interaction. The treatment difference between each LJN452 dose and placebo will be estimated and summarized at each visit by a 90% two-sided confidence interval. Dose response may be assessed using appropriate contrasts.<br>Part 2: The same repeated measures ANCOVA described above for Part 1 with the UDCA and pruritus stratification factor included as an additional fixed effect will be employed in Part 2.<br>Whole study: Summary statistics will be provided for Safety endpoints, PK parameters [redacted].
**Key words**	Primary Biliary Cirrhosis, Primary Biliary Cholangitis, PBC

[redacted]

1 Introduction

LJN452 is a highly potent agonist of the farnesyl X receptor (FXR) which modulates bile acid synthesis and detoxifying metabolism in the liver. Cholestasis is a pathological condition in which bile flow from the liver to the intestine is reduced or blocked. Causes of cholestasis include developmental abnormalities, trauma, chemical or immunological damage. Chronic cholestasis leads to fibrosis, cirrhosis and potentially the need for liver transplantation. LJN452 is expected to provide significant therapeutic benefit in patients with cholestatic conditions such as Primary Biliary Cholangitis (PBC).

1.1 Background

Cholestasis is a pathological condition in which bile flow from the liver to the gall bladder or intestine is reduced or blocked, leading to accumulation of potentially toxic biliary constituents. PBC is an autoimmune disease of unknown etiology characterized by slowly progressive cholestasis that may lead to cirrhosis, liver transplantation or death. The only approved therapy for PBC is the bile acid ursodeoxycholic acid (UDCA), however this is ineffective in approximately 40% patients, which highlights the need for new drugs.

FXR is a nuclear receptor activated by bile acids (Chen et al 2004; Makishima et al 1999; Parks et al 1999; Wang et al 1999). When activated, FXR initiates a sensitive, negative feedback loop affecting various aspects of bile acid metabolism resulting in reduced bile acid levels (Zollner et al 2006). Pharmacological activation of FXR is proposed to be an effective treatment for intrahepatic cholestasis by increasing bile flow from the liver to the intestine, reducing hepatic bile acid uptake and bile acid synthesis, increasing renal bile acid excretion, decreasing circulating bile acid levels and reducing hepatic fibrosis. The therapeutic potential of FXR agonism has been studied in Phase 2 clinical trials and a reduction in biomarkers of cholestatic disease, such as alkaline phosphatase (ALP) and γ-glutamyl transpeptidase (GGT), has been demonstrated (Mason et al 2010a; Kowdley et al 2011; Hirschfield et al 2015).

LJN452 is a highly potent, specific and orally available FXR agonist. This is a study to determine the safety, tolerability, pharmacokinetics efficacy of LJN452 in patients with PBC.

1.1.1 Relevant data summary

Detailed background information on the chemistry, pharmacology, toxicology and pharmacokinetics of LJN452 are given in the Investigator's Brochure. The most relevant data for the present study are summarized in the sections below.

1.1.1.1 Preclinical data

Pharmacology

In vitro pharmacology studies demonstrate that LJN452 is a potent human FXR agonist with an EC_{50} of 0.2 nM and 0.3 nM in a cell-based FXR reporter gene assay and in a biochemical co-activator interaction assay, respectively, with >30,000 fold selectivity over other nuclear receptors. Furthermore, LJN452 demonstrated no off-target binding/activity (up to 30 µM) in a broad specificity-screening panel of receptors, transporters, ion channels, nuclear receptors or enzymes.

[redacted]

FXR agonism has been shown to modify gene expression of a number of enzymes and transporters responsible for drug metabolism and secretion (Calkin and Tontonoz 2012). The time course of effects of LJN452 on the bile salt export pump (BSEP) in preclinical species is detailed in the Investigator's Brochure in addition to potential induction of drug metabolizing enzymes. Drug secretion into bile is typically considered a safe route of clearance. However, biliary delivery of some drugs or their reactive metabolites to the intestinal tract may evoke adverse consequences due to direct toxic actions or indirect disruption of intestinal homeostasis. For example, delivery of reactive metabolites of diclofenac via MRP2 into bile has been linked with NSAID enteropathy (Treinen-Moslen and Kanz 2006). [redacted]

In vivo a single oral dose of LJN452 in naïve rats ranging from 0.03-10 mg/kg demonstrated a dose-dependent increase in expression of FXR target genes (liver BSEP and small heterodimer partner (SHP), ileum SHP and FGF15). Dosing of naïve rats with LJN452 (p.o., q.d. for 2 weeks) resulted in modulation of gene expression and clinically relevant pharmacodynamic and biomarker (FGF15) changes at doses as low as 0.01 mg/kg/day.

[redacted]

[redacted]

[redacted]

Pharmacokinetics

LJN452 has moderate oral bioavailability in animals (≥10%). Plasma terminal T1/2 was moderate to long. Clearance and volume distribution were low to moderate. After repeated doses, the exposure was dose proportional with no consistent sex difference. No accumulation was observed in dog, but mild accumulation noted in rat. LJN452 is highly plasma protein bound (>97%). [redacted]

[redacted]

[redacted]

[redacted]

[redacted]

[redacted]

[redacted]

[redacted]

1.2 Study purpose

The purpose of Part 1 is to assess the safety, tolerability and efficacy of LJN452 administration for 4 weeks on markers of cholestasis in patients with Primary Biliary Cholangitis (PBC), to enable decisions regarding the further development of LJN452 in the indication of PBC. In addition, data obtained in Part 1 will inform dose selection for Part 2.

The purpose of Part 2 is to further assess the safety and efficacy of two dose levels of LJN452, when administered for longer (12 weeks) and in a wider patient population (both those taking and not taking UDCA). This will provide data to support further development of LJN452 in patients with PBC.

[redacted]

2 Study objectives

2.1 Primary objectives

Objective	Endpoint
• To determine the effect of LJN452 on cholestatic markers in patients with PBC. More specifically, in Part 2 to determine the dose-response relationship of LJN452 on GGT following 12 weeks of treatment	• Liver function tests, specifically GGT
• To determine the safety and tolerability of daily dosing of LJN452 in patients with PBC	• All safety endpoints (including physical exam, vital signs, ECG, safety laboratory tests)<br>• Serious adverse events, adverse events

2.2 Secondary objectives

Objective	Endpoint
• To evaluate the pharmacokinetics (PK) of LJN452 in patients with PBC	• PK Blood collection
• To evaluate the change in overall disease specific quality of life	• PBC40
• To determine the change in the itch domain of PBC40 questionnaire	• PBC40
• To evaluate the change in itch based on 100 mm visual analog score (VAS)	• Global Itch Visual Analogue Scale
• Part 2: To determine the dose-response relationship of LJN452 on ALP in patients with PBC following 12 weeks of treatment	• ALP at baseline and week 13

[redacted]

[redacted]

3 Investigational plan

3.1 Study design

This is a randomized, double-blind, placebo-controlled, multi-part study to assess safety, tolerability and efficacy of LJN452 in patients with Primary Biliary Cholangitis (PBC).

Part 1 uses an escalating multiple dose design in PBC patients with incomplete biochemical response to, but still taking, ursodeoxycholic acid (UDCA).

Part 2 is a parallel-group, 12-week study to assess the safety, tolerability and efficacy of two doses of LJN452 compared to placebo in PBC patients with an incomplete biochemical response to, but still taking, UDCA or those not currently taking UDCA. The two LJN452 doses for Part 2 will be selected based on safety, tolerability and efficacy data from Part 1.

Once approximately 40 patients from Part 2 have completed 8 weeks of treatment, an interim analysis will be conducted to re-assess the sample size for Part 2. Recruitment will continue while the interim analysis is performed. Figure 3-1 shows how Parts 1 and 2 are connected.

[redacted]

Figure 3-1 Study design

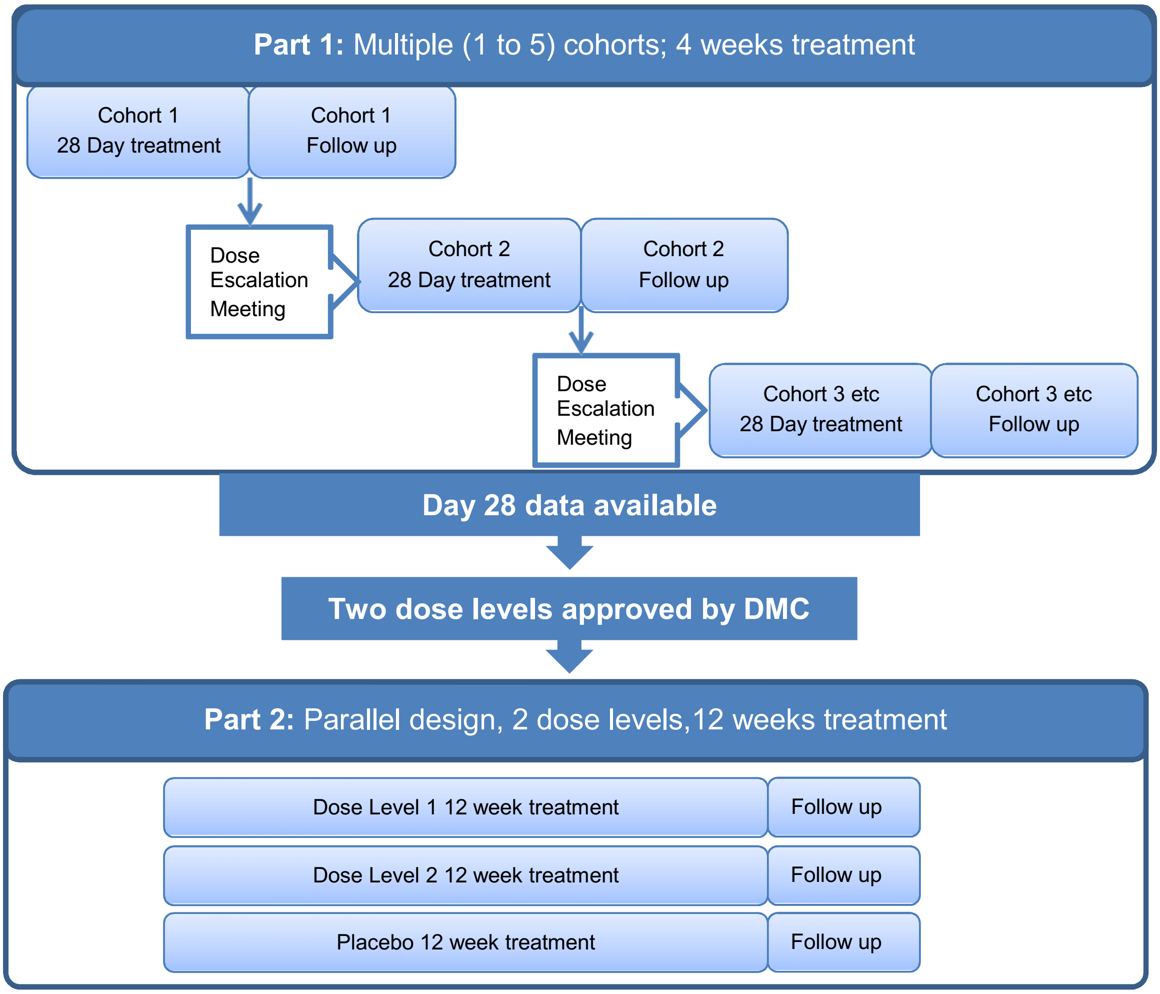

Part 1

A minimum of 2 and maximum of 5 cohorts of approximately 15 patients with PBC with incomplete biochemical response, but still taking UDCA treatment will be enrolled into Part 1, with the aim of a minimum of 12 patients per cohort completing. Patients withdrawing for reasons other than AEs may be replaced throughout the study. Patients withdrawing from a cohort may not be enrolled in a subsequent cohort.

Figure 3-2 shows the study design of Part 1. At the beginning of Part 1, a screening visit will take place, where a patient's eligibility to enter the study will be assessed. Eligible patients will be admitted to the study site and re-evaluated for inclusion / exclusion criteria during the baseline visit. All baseline safety evaluation results must be available and reviewed prior to first dosing.

[redacted]

Figure 3-2 Study design Part 1

Study Day		1	7	14	21	28	29	56	84
Study visit ↑	↑	↑	↑	↑	↑	↑	↑ (optional)	↑	↑
Screening	BL	<-	4 week treatment period			->		Follow up	EOS

BL = Baseline assessments
EOS = End of Study visit
↑ = Study visit ↑ = Optional study visit

Patients will be asked to arrive at the study site approximately 2 hours prior to dosing on Day 1. Patients in Cohort 1 will be randomized in a 2:1 ratio to receive the starting dose of 0.03 mg LJN452 or placebo. Safety assessments will be performed for 4 hours post dose and pharmacokinetic [redacted] assessments will be performed for up to 8 hours post first dose (see Assessment schedule for details). Prior to discharge, the patients will be given a supply of LJN452 or placebo and instructed to take first thing in the morning with water, prior to eating every day.

Patients will then return on Days 7, 14 and 21 for safety, pharmacokinetic [redacted] assessments and dosing. Patients will be asked to return to the study site on Day 28, for the final dosing visit, when safety, pharmacokinetic [redacted] assessments will be performed. Patients will be asked to return to the study site for an optional ambulatory visit on Day 29, where 24 hour post dose pharmacokinetic [redacted] assessments will be performed.

Patients are asked to return to the study site on approximately Day 56 for follow up assessments. An end of study visit will take place at approximately Day 84.

A dose escalation review and an interim analysis are planned when at least 12 patients have completed dosing and Day 28 assessments in Cohort 1. The main purpose of the interim analysis will be to evaluate the observed variability in GGT levels and, if needed, consider a sample size re-estimation.

The Lead PI from each active country and Investigators with patients recruited into the cohort will be invited to be involved in the review of blinded data from that cohort and the decision on the dose for the next cohort of subjects (if applicable). Full details of the process are outlined in the Dose Escalation Charter and the SOM.

Additional cohorts will follow the same schedule as Cohort 1. Further cohorts may be added depending on emerging safety and PK/PD data. Additional interim analyses may be performed after a minimum of 12 patients in each cohort finish dosing and Day 28 assessment. The sponsor will be unblinded and the Clinical Trial Team may be unblinded when all subjects in a cohort have completed 28 day of study hence at the time of the planned IAs.

[redacted]

Part 2

Approximately 88 patients with PBC, with an incomplete biochemical response to, but still taking, UDCA or those not currently taking UDCA are planned to be recruited into Part 2, with the aim that 80 will complete the study. Patients who participated in Part 1 may be recruited to Part 2, provided they meet all required eligibility criteria and have not received study medication within 3 months of Randomization, (or longer if required by local regulations) – see exclusion criteria.

Figure 3-3 Study design Part 2

Week	-12 to -2	1	2	3		5				9				13	17
Study visit	↑	↑	TC	↑		↑				↑				↑	↑
Screening		<------------------- 12 week treatment period ------------------->													Follow up
		BL													EOS

BL = Baseline assessments (performed pre-dose Day 1)

↑ = Study visit

TC = Telephone Contact

EOS = End of Study visit

A screening visit will take place, where patient's eligibility to enter the study will be assessed.

Eligible patients will be asked to arrive at the study site approximately 2 hours prior to dosing on Day 1 to perform Baseline assessments.

Patients will be randomized in a 3:3:2 ratio to receive either LJN452 Dose level 1, LJN452 Dose level 2 or placebo. Following drug administration, safety, pharmacokinetic [redacted] assessments will be performed at two hours post dose.

Prior to discharge, the patients will be given a supply of LJN452 or placebo and instructed to take their dose first thing in the morning with water, prior to eating every day. New supplies will be dispensed on a monthly basis during regular study visits.

Patients will then return at weeks 3, 5, 9 and 13 for safety, tolerability, pharmacokinetic [redacted]. A telephone contact will be made one week after start of dosing to check their progress and check they have no questions.

Patients will be asked to return to the study site in week 17 for follow up assessments and to complete their end of study assessments.

If a patient has a medically important AE that is considered related to study drug and which has not resolved or stabilized at their scheduled end of study visit (week 17), they will be followed, until resolution or stabilization of the AE.

The following will be considered as medically important:

i) An AE with CTCAE Grade ≥3

ii) SAE

iii) hepatic and renal events should be followed up per the guidance in Table 15-1 and Table 16-1

[redacted]

These patients will be monitored by the investigator and have unscheduled visits to collect safety and/or laboratory data. It is advised that the first follow up of these patients with such events will be scheduled at approximately week 21, or sooner based on Investigator's discretion. If events are not resolved or stabilized at this time point, further follow up will be scheduled at the Investigator's discretion and unscheduled visits will be used to collect safety and/or laboratory data. As soon as the event is resolved and/or stabilized, and as long as there are no additional medically important AEs from the treatment period ongoing, no further follow up is required.

3.2 Rationale of study design

This randomized, multi-center, double-blind, placebo-controlled, parallel-group, multi-part study is designed to evaluate safety, tolerability and efficacy of LJN452 (in doses of 0.01 mg and above) relative to placebo in patients with PBC. This will be assessed by a reduction in the cholestatic marker GGT after 4 weeks of double-blind treatment in Part 1 and 12 weeks of double-blind treatment in Part 2.

In Part 1 up to 5 dose levels will be explored to allow characterization of dose exposure response relationship in PBC patients. Randomization in Part 1 has been chosen on a 2:1 ratio in order to rapidly obtain safety and PK data for LJN452. Since 2 cohorts are initially anticipated, the chosen ratio will provide a similar number of patients treated at each dose level to compare with placebo.

In Part 2 two dose levels of LJN452 will be selected based on the safety, tolerability and efficacy data from Part 1.

Randomization in Part 2 has been chosen on a 3:3:2 ratio to enable more safety data to be obtained from patients receiving LJN452. Thus assessment of the safety, tolerability and efficacy of 12 weeks treatment with two dose levels of LJN452 will be assessed, to provide data to support dose selection for future development. In addition, an interim analysis is planned in Part 2 to determine the effect of LJN452 on GGT and itch parameters and to assess the variability for sample size re-estimation, if needed.

In order to maintain the scientific integrity of the study, the investigator and patient will remain blinded to their treatment assignment until database lock (either at the end of the study, or earlier in the event of a staggered lock of Part 1 or cohorts in Part 1).

The use of multiple cohorts of ascending dose in Part 1 will allow evaluation of safety, PD and PK starting at a dose below the maximum dose tested so far in healthy volunteers. The multiple cohort dose escalation design will allow characterization of the dose-exposure-response relationship in PBC patients. Escalation will depend on safety parameters and the increment in dose will not exceed 1.67 fold at doses greater than 0.09 mg.

Part 1 will provide safety and efficacy data for LJN452 in a population who have not had satisfactory biochemical response to the first line standard of care UDCA and hence are at increased risk of disease progression to hepatic decompensation, transplantation or death. Data obtained in Part 1 will allow selection of doses to be used in Part 2.

[redacted]

Part 2 will provide safety and efficacy data from a longer treatment duration with LJN452 in the same population as well as widening the population to include patients who are not currently taking standard of care, UDCA (patients will be stratified across the treatment groups based on their UDCA status). This group has a high unmet clinical need and is likely to make up a significant minority population in later phase studies.

Patients will also be stratified across the treatment groups based on the severity of any pruritus. Patients with a PBC-40 score at screening of <7 will be classed as low severity and those with a score ≥7 will be classed as high severity.

[redacted]

[redacted]

[redacted]

3.4 Rationale for choice of comparator

A placebo will be used as a comparator in this study to ensure the effects of LJN452 are related to the study drug. Recently, the FXR agonist obeticholic acid (Ocaliva™, Intercept Pharmaceuticals) was approved for those with PBC non-responsive to or intolerant of UDCA. However, obeticholic acid is not universally utilized for treatment of PBC. Furthermore, PBC patients with severe itch were excluded from Phase 3 POISE study (Nevens et al 2016). In order to allow those with severe itch to be enrolled into the study placebo will be used as a comparator.

[redacted]

3.5 Purpose and timing of interim analyses/design adaptations

The first interim analysis will be performed when at least 12 patients in Cohort 1 have completed 4 weeks of dosing at 0.03 mg and Day 28 post dose assessments. The purpose of this interim analysis is to assess patient safety, to evaluate the assumption on intra-subject variability of GGT, which was used for sample size calculations, and to select a dose for the next cohort. An ANCOVA model will be fitted to estimate the change from baseline in GGT and its variability, and an unblinded sample size re-estimation conducted. The results may be used to adjust the size of the next cohort.

Doses in subsequent cohorts of Part 1 will be decided based on accumulated information collected from all previous cohorts including safety, PK and PD data.

Additional interim analyses will be performed when at least 12 patients in subsequent cohorts of Part 1 have completed 4 weeks of dosing and Day 28 assessments. Placebo data from all cohorts will be pooled in the ANCOVA model to evaluate the treatment difference between each dose level of LJN452 versus placebo. No multiplicity adjustment will be made due to the exploratory nature of the study.

Results from Part 1 will be used to select the doses for Part 2, which may be initiated prior to the completion of Part 1. Part 2 will not be initiated if the estimated change from baseline in GGT at Day 28 is smaller than 20% at the highest dose level of LJN452 in Part 1.

Unblinded interim analysis results from Part 1 will be reviewed by an internal Novartis Interim Analysis Team which may include some CTT members. The Interim Analysis Team may communicate interim results to relevant Novartis teams for information, consulting and/or decision purposes.

In Part 2 of the study, an interim analysis is planned when approximately 40 patients have completed their Week 8 visit. The purpose of this interim analysis is to review the sample size (calculated based on GGT and ALP changes) to assess if it is also adequate to provide precise enough treatment difference estimates in itch parameters, a key tolerability endpoint.

This study will utilize a staggered lock of the clinical database, so that the data can be locked after completion of Part 1, or a set of Cohorts in Part 1.

Once a Cohort's data has been locked, treatment codes for the relevant cohort(s) will be released and made available for data analysis.

The results from the locked Part 1 interim analysis can be communicated beyond Novartis to groups including, but not limited to individuals treating the study's subjects, Health Authorities and reported on clinical registries.

Additional interim analyses [redacted] may be conducted to support decision making concerning the current clinical study, the sponsor's clinical development projects in general or in case of any safety concerns. No dose escalation is planned in Part 2.

Additional information is presented in Section 11.8.

[redacted]

3.6 Risks and benefits

There is no benefit expected for subjects participating in this short-term study.

Preliminary data from study CLJN452X2101 have demonstrated isolated increases in transaminases ALT and AST [redacted]. These changes normalized spontaneously have not been observed at the proposed starting dose.

The risk to subjects in this trial will be minimized by adherence to the eligibility criteria, close clinical monitoring, and stopping rules.

A maximum of 500 mL of blood is planned to be collected over a period of 16 weeks, from subjects as part of the study (Part 1 sample volume will be approximately 490 mL and Part 2 will be approximately 450 mL). Additional samples for monitoring of any safety findings may be required, and would be in addition to this volume. This is not considered to be a risk for this population.

There may be unknown risks of LJN452 which may be serious and unforeseen.

4 Population

The study population will be comprised of patients with PBC.

In Part 1, a total of approximately 15 patients will be enrolled in each cohort of the study and randomized.

In Part 2, a total of approximately 88 patients will be enrolled and randomized. Final sample size for Part 2 will be informed by the interim analysis.

The investigator must ensure that all subjects being considered for the study meet the following eligibility criteria. No additional criteria should be applied by the investigator, in order that the study population will be representative of all eligible subjects.

Subject selection is to be established by checking through all eligibility criteria at screening and baseline (for Part 1). A relevant record (e.g., checklist) of the eligibility criteria must be stored with the source documentation at the study site.

Deviation from **any** entry criterion excludes a subject from enrollment into the study.

In Part 1 replacement subjects may be enrolled to replace subjects who discontinue the study for reasons other than safety.

4.1 Inclusion criteria for Parts 1 and 2

Patients eligible for inclusion in this study have to fulfill all of the following criteria:

1. Written informed consent must be obtained before any assessment is performed.
2. Age ≥ 18 years
3. Diagnosis of PBC as demonstrated by the presence of at least 2 of the following 3 diagnostic criteria:
   - History of Alkaline Phosphatase elevated above upper limit of normal for at least 6 months

[redacted]

- Positive antimitochondrial antibodies (AMA) titer or if AMA negative or in low titer (<1:80) PBC specific antibodies (anti-GP210 and/or anti-SP100 and/or antibodies against the major M2 components (PDC-E2, 2-oxo-glutaric acid dehydrogenase complex))
- Previous liver biopsy findings consistent with PBC

4. At least 1 of the following markers of disease severity:
   - ALP ≥ 1.67× upper limit of normal (ULN)
   - Total bilirubin > ULN but < 1.5 × ULN

   In addition, patients must meet the following biochemical criteria at enrollment
   - ALT or AST ≤ 5 × ULN
   - Total bilirubin ≤ 1.5 × ULN
   - INR ≤ ULN
5. Subjects must weigh at least 40 kg to participate in the study, and must have a body mass index (BMI) within the range of 18 - 40 kg/m^2. BMI = Body weight (kg) / $[Height(m)]^2$.
6. Able to communicate well with the investigator, to understand and comply with the requirements of the study.

4.1.1 Additional Inclusion Criteria for Part 1

All patients enrolled in Part 1 must meet the following criteria:

1. Taking UDCA for at least 12 months, or for at least 6 months and has reached maximal response to UDCA with a plateau in alkaline phosphatase, (with no changes in dose for ≥ 3 months) prior to Day 1.

4.1.2 Additional Inclusion Criteria for Part 2

All patients enrolled in Part 2 must meet the following criteria:

1. Patients not currently taking UDCA and who have not taken UDCA for ≥ 3 months prior to Day 1 (for example UDCA naïve patients, patients who were unable to tolerate UDCA or had stopped taking due to lack of efficacy) OR Patients taking UDCA for at least 12 months, or for at least 6 months and has reached maximal response to UDCA with a plateau in alkaline phosphatase, (with no changes in dose for ≥ 3 months) prior to Day 1

4.2 Exclusion criteria for Parts 1 and 2

Patients fulfilling any of the following criteria are not eligible for inclusion in this study. No additional exclusions may be applied by the investigator, in order to ensure that the study population will be representative of all eligible patients.

1. Presence of other concomitant liver diseases including:
   - Active Hepatitis B or C virus (HCV, HBV) infection
   - Primary sclerosing cholangitis (PSC)
   - Alcoholic liver disease
   - Definite autoimmune hepatitis
   - Nonalcoholic steatohepatitis (NASH)

[redacted]

- Gilbert's Syndrome

2. Women of child-bearing potential, defined as all women physiologically capable of becoming pregnant, unless they are using highly effective methods of contraception for 30 days before randomization, and will continue to use it during dosing and for 30 days following the end of treatment. Highly effective contraception methods include:
   - Total abstinence (when this is in line with the preferred and usual lifestyle of the subject). Periodic abstinence (e.g., calendar, ovulation, symptothermal, post-ovulation methods) and withdrawal are not acceptable methods of contraception
   - Female sterilization (have had surgical bilateral oophorectomy with or without hysterectomy) or tubal ligation at least six weeks before taking study treatment. In case of oophorectomy alone, only when the reproductive status of the woman has been confirmed by follow up hormone level assessment
   - Male sterilization (at least 6 m prior to screening). For female subjects on the study, the vasectomized male partner should be the sole partner for that subject
   - Use of oral (estrogen and progesterone), injected or implanted combined hormonal methods of contraception or placement of an intrauterine device (IUD) or intrauterine system (IUS), or other forms of hormonal contraception that have comparable efficacy (failure rate <1%), for example hormone vaginal ring or transdermal hormone contraception

   In case of use of oral contraception women should have been stable on the same pill for a minimum of 3 months before taking study treatment and should complete at least one full cycle after completing dosing.
3. Cirrhosis with complications, including history or presence of:
   - Variceal bleed
   - Uncontrolled ascites
   - Encephalopathy
   - Spontaneous bacterial peritonitis
4. Significant hepatic impairment as defined by Child-Pugh classification of B or C, history of liver transplantation, current placement on a liver transplant list or current Model for End Stage Liver Disease (MELD) score ≥15.
5. History of medical conditions other than PBC that may cause increases in ALP (e.g., Paget's disease).
6. History of malignancy of any organ system (other than localized basal cell carcinoma of the skin or treated cervical intraepithelial neoplasia), treated or untreated, within the past 5 years, regardless of whether there is evidence of local recurrence or metastases.

[redacted]

8. History of non-adherence to medical regimens, or patients who are considered to be unable to reliably comply with the requirements of the study.

[redacted]

9. Donation or loss of 400 mL or more of blood within eight (8) weeks prior to initial dosing, or longer if required by local regulation.
10. Use of other investigational drugs or immunosuppressive drugs at the time of enrollment, or within 5 half-lives/30 days of randomization, whichever is longer; (or longer if required by local regulations). Use of high dose oral steroids to treat co-morbid conditions (e.g. airways disease) will be allowed but must be properly documented as such in concomitant medications.
11. Pregnant or nursing (lactating) women, where pregnancy is defined as the state of a female after conception and until the termination of gestation, confirmed by a positive hCG laboratory test.
12. History of drug or alcohol abuse within the 12 months prior to dosing.
13. Significant illness which has not resolved within two (2) weeks prior to initial dosing.
14. Acute or chronic renal disease with a screening serum creatinine > ULN.
15. Elevated liver function tests at screening defined as follows:
    - potential Hy's Law case (defined as ALT or AST > 3 × ULN and TBL > 2 × ULN without notable increase in ALP to > 2 × ULN) or
    - ALT or AST > 3 × ULN combined with INR > 1.5.
    - Total bilirubin > ULN combined with albumin outside of the normal range.
16. Currently taking obeticholic acid or have taken obeticholic acid within 30 days of Randomization.
17. Participation in CLJN452X2201 and received study medication within three months of Randomization (or longer if required by local regulations).
18. History of immunodeficiency diseases, including a positive HIV (ELISA and Western blot) test result.

No additional exclusions may be applied by the investigator, in order to ensure that the study population will be representative of all eligible patients.

5 Restrictions for Study Subjects

During recruitment, screening/informed consent review, and baseline visit, the subjects must be informed and reminded of the following restrictions:

5.1 Contraception requirements

Please refer to exclusion criteria (Section 4) for details of contraception requirements for the study.

[redacted]

5.2 Prohibited treatment

Obeticholic acid: 30 days prior to randomization, during treatment and until completion of the End of Study visit.

Investigational drugs: within 5 half-lives/30 days of randomization, whichever is longer; (or longer if required by local regulations).

Immunosuppressive drugs: within 30 days of randomization. Exception; the use of high dose oral steroids to treat co-morbid conditions (e.g. airways disease) will be allowed but must be properly documented as such in concomitant medications.

No medication or herbal remedies likely to have an impact on LJN452 metabolism will be allowed from the first dosing until day 28 (Part 1) or End of Study (Part 2) evaluations have been conducted. [redacted]

[redacted]

A full list of prohibited medications will be provided in the SOM.

5.3 Dietary restrictions and smoking

- No alcohol consumption is allowed for 8 hours before dosing and each study visit until Study Completion evaluation.
- In Part 1: all subjects will fast (i.e., no food and liquid except water) for at least 8 hours prior to administration of study drug and will continue to fast for at least 2 hours thereafter. No fluid intake apart from the fluid given at the time of drug intake is allowed from 2 h before until 2 h after dosing.
- In Part 2: all patients will fast (i.e. no food and liquid except water) for at least 8 hours prior to each dose and should refrain from drinking for at least 30 min post dose, and refrain from eating for at least 60 min post dose. A light breakfast may be taken after 60 minutes.

5.4 General restrictions

- No strenuous physical exercise (e.g., weight training, aerobics, football) until after Study Completion evaluation.

Note: Patients should be reminded by site staff that on the day of a study visit during the treatment period to not take their study medication at home that morning, and to come fasted and bring their study medication with them to take once their pre-dose assessments have been completed.

[redacted]

6 Treatment

6.1 Study treatment

Details on the storage and management of study medication, randomization and instructions for prescribing and taking study treatment are outlined in Section 3 of the Site Operations Manual.

6.1.1 Investigational treatment

The investigational drug, LJN452 0.01 mg, 0.03 mg or 0.1 mg and matching placebo will be prepared and supplied by Novartis as a single blind patient specific packs to be dispensed by the unblinded pharmacist at the investigator site.

In Part 1, once a patient is confirmed as eligible after completion of their Baseline assessments, a Randomization Number can be requested, following the process in the Site Operations Manual. The Pharmacist/unblinded designee then dispenses the study medication according to the treatment assignment for that Randomization number, according to the supplied randomization schedule (provided via Treatment Allocation Cards or Electronic Randomization list).

In Part 2, once a patient is confirmed as eligible after completion of their Screening assessments, the site will contact the Interactive Response Technology (IRT) system, which will randomize the patient to the appropriate treatment. For further details, please see the Site Operations Manual and NIRT Site user manual.

6.2 Treatment arms

Part 1

Patients in each cohort will be assigned to one of the following treatment arms in a ratio of 2:1. The number of patients enrolled in Cohorts 2-5 may change based on data from IAs.

Cohort 1:

- LJN452 0.03 mg daily for 28 days (n=10) (given as 3 x LJN452 0.01 mg)
- LJN452 Placebo daily for 28 days (n=5)

Cohort 2:

- LJN452 0.06 mg daily for 28 days (n=10)
- LJN452 Placebo daily for 28 days (n=5)

Cohort 3:

- LJN452 0.09 mg daily for 28 days (n=10)
- LJN452 Placebo daily for 28 days (n=5)

Cohorts 4-5 (if required) may receive different doses:

- LJN452 TBD mg daily for 28 days (n=10)
- LJN452 Placebo daily for 28 days (n=5)

[redacted]

Part 2

Patients in Part 2 will be assigned to one of the following treatment arms in a ratio of 3:3:2.

- LJN452 Dose level 1: TBD [redacted] daily for 12 weeks (n=33)
- LJN452 Dose level 2: TBD [redacted] daily for 12 weeks (n=33)
- LJN452 Placebo daily for 12 weeks (n=22)

Part 2 dose levels will be selected based on a balance of safety, tolerability and efficacy. The dose levels selected in Part 2 will be approved by the DMC and [redacted]

6.2.1 Permitted dose adjustments and interruptions of study treatment

Part 1

Within a cohort, once a dose is decided on, study drug dose adjustments are not permitted. Interruption to study drug may occur based on the criteria outlined in Table 15-1 (Liver events) and Table 6-1 (Pruritus event definitions and required actions).

In case of notable adverse events or safety concerns during dose escalation of the study, the following changes to the next planned dose level may be considered:

- Administration of a dose below the starting dose
- Administration of an intermediate dose between the current and preceding dose
- Administration of an intermediate dose between the current and next planned dose
- Repeated administration of the current dose
- Termination of any further dose escalation

These changes must be recorded on the Dosage Administration Record CRF.

Part 2: Option for dose reduction when ALT or AST > 2 × Baseline value AND ≤8 x ULN

For patients who experience ALT or AST elevations meeting the definitions outlined in Table 15-1, a dose interruption or dose reduction to the next lower dose level, (or to placebo if receiving the lowest dose level) is permitted. One dose reduction step is allowed per patient. If, despite being on a reduced dose, a patient has persistent (i.e. remains > 90% of the original elevated level) or new ALT or AST elevation, they must be discontinued from study treatment. Increasing the dose to the original level is not permitted.

If the patient has symptoms or other LFTs are also elevated please refer to Appendix 1 for appropriate actions.

Patients with AST or ALT values meeting the requirement will be instructed to return to the clinic for an additional visit, where repeat tests will be performed, including a physical examination and laboratory assessment including ALT, AST, GGT, alkaline phosphatase, total bilirubin, albumin and PT/INR.

[redacted]

The central laboratory should be used, if this is not possible, the repeats can be performed at a local laboratory to monitor the safety of the patient. If tests are performed at local laboratory, results will be captured in source documents only. In all cases, a physical examination should be performed and recorded in source documents.

Table 15-1 in Appendix 1 outlines the required testing frequency and resultant actions dependent on the outcome of the repeat assessments.

If the additional laboratory evaluation confirms the increase of ALT and/or AST values, the study treatment will be reduced to the next dose level in a blinded manner (Table 6-1). The procedure for dose reduction is outlined in the Site Operations Manual.

If the additional laboratory evaluation does not confirm the significant increase of ALT values, the patient will continue with the assigned/original dose level.

If the additional laboratory evaluation shows a higher elevation of ALT and/or AST (e.g. > 8× ULN) as compared to the original elevation (e.g. > 5xULN but ≤ 8xULN), the guidance for this higher elevation (e.g. > 8× ULN) should be followed.

Table 6-1 Part 2 Permitted dose reductions

Original treatment (daily dose)	Reduced treatment (daily dose)
Dose Level 1	Placebo
Dose Level 2	Dose Level 1
Placebo	Placebo

Repeat/second dose reductions are not permitted. Returning to the original treatment is not permitted.

Patients with a persistent or a second cycle of elevated ALT or AST while on the reduced dose will be instructed to return to the clinic for repeat tests as outlined in Table 15-1. If the repeat tests confirm the elevated ALT or AST values, the patient will discontinue study treatment. If the repeat tests do not confirm elevation, the patient will continue with the reduced dose.

6.3 Treatment assignment

Randomization numbers will be assigned in ascending, sequential order to eligible subjects (see Site Operations Manual for details).

The randomization numbers will be generated using the following procedure to ensure that treatment assignment is unbiased and concealed from subjects and investigator staff.

In Part 1 a randomization list will be produced by or under the responsibility of Novartis Drug Supply Management using a validated system that automates the random assignment of treatment arms to randomization numbers in the specified ratio. The investigator will enter the randomization number on the CRF.

[redacted]

In Part 2 a subject randomization list will be produced by the IRT provider or by a delegate under their supervision, using a validated system that automates the random assignment of treatments to randomization numbers. These randomization numbers are linked to the different treatment arms.

In Part 2 randomization will be stratified by whether the patient is taking UDCA or not and the severity of pruritus.

Patients who participated in Part 1 may be enrolled in Part 2, provided they meet all the eligibility criteria. They will be assigned a new screening and randomization number. The screening number a subject had in Part 1 will be recorded as part of the CRF data collected for Part 2, if a subject participates in both parts.

The randomization scheme for all subjects will be reviewed and approved by a member of the Randomization Office.

6.4 Treatment blinding

This is a double-blind study: subjects, investigator staff (with the exception of unblinded pharmacy staff or authorized designee responsible for dispensing study medication), persons performing the assessments, and data analysts will remain blinded to the identity of study treatments.

The identity of the treatments will be concealed by the use of study drugs that are all identical in packaging, labeling, schedule of administration, appearance and odor. In Part 2, the blind for the different dose levels will be maintained through the use of a double-dummy design, with additional placebo capsules given in active treatment groups when needed to maintain the blind.

Randomization data are kept strictly confidential, and are accessible only to authorized personnel (unblinded pharmacist or authorized designee), until un-blinding of the trial as described in the table in the Blinding levels table in the Site Operations Manual.

The bioanalyst will request a copy of the randomization to facilitate analysis of the samples. The bioanalyst will provide the sample data to the team under blinded conditions. The bioanalyst will keep this information confidential until interim analysis (Part 1 only) or final clinical database lock.

Unblinding will only occur in the case of patient emergencies (see Section 6.5), at the time of the interim analysis and upon database lock, either at the conclusion of the study or completion of a Cohort or Part of the study.

Part 1

The sponsor will be unblinded and the randomization released to the CTT including the Modeler when the interim analysis is performed after subjects in a cohort have completed Day 28 of the study.

The Interim Analysis Team may communicate interim analysis results (e.g., dose escalation decision, evaluation of PoC criteria or information needed for planning/modifying another study) to relevant Novartis teams for information, consulting and/or decision purposes.

[redacted]

Cohorts in Part 1 may have their data cleaned and locked before the conclusion of the whole study. In event of database lock for a Cohort or Part of the study, treatment codes for the subjects included in the locked cohort or part will be released and the data may be disseminated.

Part 2

The sponsor will remain blinded to the identity of study treatments during the study. An unblinded interim analysis will be performed by a study independent statistician and programmer when approximately 40 patients have completed their Week 8 visit in Part 2. A selected Novartis team not involved in the clinical conduct of the study (DMC) will be unblinded to the Week 8 results and will advise on whether sample size adjustment is required (see Section 11.8).

6.5 Emergency breaking of assigned treatment code

Emergency un-blinding should only be undertaken when it is essential to treat the subject safely and efficaciously. Most often, study treatment discontinuation and knowledge of the possible treatment assignments are sufficient to treat a study subject who presents with an emergency condition.

In Part, 1 a complete set of emergency code break cards will be provided to the investigator sites and a complete set will be available at Novartis. All code break cards must be retained until the end of the study and returned to Novartis. They must be stored in a secure place but be accessible in case of emergency. The investigator will receive a blinded code break card for each subject, with the details of drug treatment covered by a removable, scratch-off cover. In an emergency, the scratch-off cover can be removed to determine the treatment. The scratch-off covers are not to be removed for any reason other than an emergency. When the investigator removes the scratch-off cover he/she must note the date, time, and reason for removing it and retain this information with the case report form documentation. **The unblinded treatment code should not be recorded on the CRF**. The investigator must also immediately inform the Novartis local monitor that the code has been broken.

In Part 2 emergency treatment code breaks are performed using the IRT. When the investigator contacts the system to break a treatment code for a subject, he/she must provide the requested subject identifying information and confirm the necessity to break the treatment code for the subject. The investigator will then receive details of the investigational drug treatment for the specified subject and a fax or email confirming this information. The system will automatically inform the study monitor for the site and the Study Team that the code has been broken.

It is the investigator's responsibility to ensure that there is a procedure in place to allow access to the code break cards or IRT in case of emergency. If appropriate, the investigator will inform the subject how to contact his/her backup in cases of emergency when he/she is unavailable.

[redacted]

6.6 Treatment exposure and compliance

Pharmacokinetic parameters (measures of treatment exposure) will be determined in all subjects treated with LJN452, as detailed in Section 8.5.

To aid compliance, patients will be issued with a paper diary to record the time of dosing throughout the study. Information collected will include a confirmation that the patient took their UDCA and the time they took their study drug. In addition, patients will be asked to record details of any medication used in response to any adverse events of itch.

The Investigator (or deputy) should review the diary with the patient to make sure they are familiar with the diary and understand what information needs to be completed.

The data collected will be kept as source documentation.

6.7 Recommended treatment of adverse events

Medication used to treat AEs must be recorded on the Concomitant medications/Significant non-drug therapies CRF.

6.8 Rescue medication

Use of rescue medication must be recorded on the Concomitant medications/Significant non-drug therapies CRF after start of study drug. Patients with high pruritus scores at baseline, or a history of pruritus, should have a suitable supply of anti-pruritic therapy at home.

6.9 Concomitant treatment

All prescription medications, over-the-counter drugs and significant non-drug therapies (including physical therapy and blood transfusions) administered or taken within the timeframe defined in the entry criteria prior to the start of the study and during the study, must be recorded on the Concomitant medications/ Significant non-drug therapies section of the CRF.

Medication entries should be specific to trade name, the single dose and unit, the frequency and route of administration, the start and discontinuation date and the reason for therapy.

7 Discontinuation and study completion

7.1 Discontinuation of study treatment

Discontinuation of study treatment for a subject occurs when study treatment is stopped earlier than the protocol planned duration. Discontinuation of study treatment can be decided by either the subject or the investigator.

Study treatment **must** be discontinued under the following circumstances:

- Subject decision - subjects may choose to discontinue study treatment for any reason at any time.
- The investigator believes that continuation would negatively impact the safety of the subject or the risk/benefit ratio of trial participation.

[redacted]

- Pregnancy.
- Hypersensitivity reaction to LJN452.
- Hepatic decompensation event such as variceal bleed, uncontrolled ascites, encephalopathy, spontaneous bacterial peritonitis.
- A Liver or Renal event indicating discontinuation as defined in Table 15-1 - Follow up requirements for Liver events or Table 15-2- Follow up requirements for renal events
- An AE with a CTCAE rating of Grade 3 that are suspected to be related to study drug (apart from pruritus, liver or renal parameters, see Table 15-1, Table 15-2 and Table 16-1 for required actions).
- An AE with a CTCAE rating of Grade 4 or higher, regardless of attribution to study drug
- Emergence of the following adverse events: Diarrhea, nausea, vomiting, recurrent positive fecal occult blood or other GI symptoms that are deemed to be intolerable.

The appropriate personnel from the study site and Novartis will assess whether study treatment should be discontinued for any subject whose treatment code has been broken inadvertently for any reason.

If discontinuation of study treatment occurs, the investigator must determine the primary reason for the subject's premature discontinuation of study treatment and record this information on the Dosage Administration CRF.

After study treatment discontinuation, at a minimum, in abbreviated visits, the following data should be collected at clinic visits or via telephone/email contact:

- new / concomitant treatments
- adverse events/Serious Adverse Events

Subjects who discontinue study treatment or who decide they do not wish to participate in the study further should NOT be considered withdrawn from the study UNLESS they withdraw their consent (see Section 7.3). Where possible, they should return for follow up assessments. If they fail to return for assessments for unknown reasons, every effort (e.g., telephone, e-mail, letter) should be made to contact them as specified in Section 7.2.1

7.2 Study completion and post-study treatment

Each subject will be required to complete the study in its entirety and thereafter no further treatment with study drug will be made available to them.

Study completion is defined as when the last subject completes their End of Study visit, and any repeat assessments associated with this visit have been documented and followed-up appropriately by the Investigator, or in the event of an early study termination decision, the date of that decision.

[redacted]

All randomized subjects should have a safety follow-up call conducted 30 days after last administration of study treatment. For most subjects completing the study per protocol this will fall before or at the same time as their end of study visit, so no call will be necessary. If however a subject will not have their end of study visit 30 days after last administration of study treatment (e.g. in the event of withdrawal of consent) a safety follow-up call must be conducted. In this instance the information collected is kept as source documentation. All SAEs reported during this time period must be reported as described in Section 9.2 and the Site Operations Manual. Documentation of attempts to contact the subject should be recorded in the source documentation.

Continuing care should be provided by investigator or referring physician.

7.2.1 Lost to follow-up

For subjects whose status is unclear because they fail to appear for study visits without stating an intention to discontinue or withdraw, the investigator should show "due diligence" by documenting in the source documents steps taken to contact the subject, e.g., dates of telephone calls, registered letters, etc. A subject should not be formally considered lost to follow-up until his/her scheduled end of study visit would have occurred.

7.3 Withdrawal of informed consent

Subjects may voluntarily withdraw consent to participate in the study for any reason at any time.

Withdrawal of consent occurs only when a subject does not want to participate in the study anymore **and** does not want any further visits or assessments **and** does not want any further study related contact **and** does not allow analysis of already obtained biologic material.

If a subject withdraws consent, the investigator must make every effort to determine the primary reason for this decision and record this information. Study treatment must be discontinued and no further assessments conducted. All biological material that has not been analyzed at the time of withdrawal must not be used. Further attempts to contact the subject are not allowed unless safety findings require communicating or follow-up.

7.4 Study Stopping rules

Study "Stopping Rules"

In Part 1, the study will be placed on hold to further enrollment, no new patients may be dosed, and the study may be stopped based on a full review of all available clinical safety data and discussion with the Investigators if any of the following occur:

- Two or more subjects on study drug experience a similar adverse event that is a CTCAE Grade 3 or higher (excluding pruritus, liver or renal parameters, see Table 15-1, Table 15-2 and Table 16-1), unless it is caused by an accident that could not reasonably be attributable to the drug.
- One subject on study drug experiences any adverse event that is CTCAE Grade 4 or higher that is classified as related to study drug.

[redacted]

- The Principal Investigator and the Sponsor consider that the number and/or severity of adverse events justify discontinuation of the study.
- The Sponsor unilaterally requests it.

In Part 2, in addition to frequent review of the emerging study data by the study team, the DMC will review safety, including AEs and laboratory parameters, on a regular basis. In addition, in the event that more than 3 patients develop an AE of CTCAE Grade 3 or higher in the same system organ class, the DMC Chair will be alerted and may call an *ad hoc* DMC meeting. Further details regarding relevant data and actions will be specified in the separate DMC charter. The DMC may call for the study to be placed on hold to further enrollment, and the study may be stopped based on a full review of all available clinical safety data.

Part 1 Cohort “Stopping Rules”

Further enrollment and dose escalation will be placed on hold pending full review of all available clinical safety data and discussion with the Investigator if any of the following occur:

Note: subjects already enrolled may continue dosing provided there are no safety issues in these subjects.

- Two or more subjects experience a similar adverse event of CTCAE Grade 2 or higher severity (apart from pruritus, ALT or AST elevation; see Table 15-1, Table 15-2 and Table 16-1) suspected to be related to the study drug.
- Two or more subjects experience a similar adverse event that is a CTCAE Grade 3 or higher severity regardless of drug causality, unless it is caused by an accident that could not reasonably be attributable to the drug.
- One subject has an adverse event of CTCAE Grade 4 or higher.
- Two or more subjects with an elevation of serum TBL to greater than 2 × ULN (or 2 × baseline if initially elevated) or an increase in aminotransferase enzymes (ALT or AST) as described below:
    - In the case of normal baseline transaminases an increase of ALT or AST to greater than 3 × ULN.
    - In the case of initially elevated transaminases an increase of ALT or AST to greater than 2 × baseline.
- The Principal Investigator and the Sponsor consider that the number and/or severity and/or system organ class of adverse events justify discontinuation of drug administration within the cohort.
- The Sponsor unilaterally requests it.

Safety reviews will be conducted jointly between medically qualified representatives of the Sponsor and Investigator and a joint decision will be made. If a dose level is identified to be intolerable, the preceding dose level will be defined as the maximum tolerated dose.

The severity of adverse events will be graded by the study site Investigator (or his designee) based on clinical judgment and captured in the CRF AE page. This information will be used to quantify events that may lead to subject’s discontinuation or stopping dose escalation.

[redacted]

7.5 Early study termination

The study can be terminated at any time for any reason by Novartis. Should this be necessary, subjects should be seen as soon as possible and treated as a prematurely withdrawn subject. The investigator may be informed of additional procedures to be followed in order to ensure that adequate consideration is given to the protection of the subject's interests.

The investigator will be responsible for informing IRBs/IECs of the early termination of the trial.

8 Procedures and assessments

Subjects should be seen for all visits on the designated day, with the assessments performed as per schedule, within the allowed "visit/assessment window" specified in the Site Operations Manual.

Subjects will be seen at the investigational site or at an alternate location to which the Investigator has delegated responsibility. The alternate location will have all required ethical approvals in place, all staff will be appropriately trained in the protocol and assessments to be performed and will be documented on the delegation of duties log. The purpose of this flexibility, where available, is to offer patients the option of having their visits performed at a more convenient location, as it is anticipated patients may live a significant distance from the main Investigational site. Visits where this is an option are outlined in the assessment schedule.

[redacted]

Table 8-1 Part 1 Assessment schedule

Study Phase	Screening	Baseline	Dosing Period																		Follow Up		EOS
Visit Numbers[1]	V1	V2	V3							V4	V5	V6	V7								V8	V9	V777
Study Day(s)	-84 to -2	-14 to -1	1							7[2]	14[2]	21[2]	28								29	56	84
Time (post-dose)	-	-	predose	0h	1h	2h	4h	6h	8h				predose	0h	1h	2h	3h	4h	6h	8h	24h	-	-
Informed consent	X																						
Inclusion/Exclusion Criteria	S	S																					
Medical history	X	X																					
Demography	X																						
Alcohol Test and Drug Screen	S	S																					
Physical Examination	S	S	S							S			S										S
Hepatitis Screen	S																						
HIV Screen	S																						
Pregnancy test	X	X	X										X									X	X
Drug dispensing[3]				X																			
Dose administration (at site)[3]				X						X	X	X		X									
Body height	X																						
Body weight	X	X	X							X	X	X	X									X	X
Body temperature	X	X	X							X	X	X	X									X	X
Pulse rate	X	X	X			X	X			X	X	X	X									X	X
Blood pressure	X	X	X			X	X			X	X	X	X									X	X
ECG evaluation	X	X	X				X						X					X					
Hematology	X	X	X							X	X	X	X									X	X
Blood chemistry	X	X	X							X	X	X	X									X	X
Urinalysis	X	X	X							X	X	X	X									X	X
Fasting lipid panel		X	X							X	X	X	X									X	X
Liver function tests	X	X	X							X	X	X	X									X	X
alpha-fetoprotein	X																						
PK blood collection			X		X	X	X	X[6]	X[6]	X[4]	X[4]	X[4]	X		X	X	X	X	X	X[6]	X[5,6]	X	
Blood type	X																						
[redacted]																							
VAS (Visual Analog Scale)		X	X							X	X	X	X									X	X
PBC40		X	X										X									X	X
[redacted]																							
Concomitant therapies	As required																						
Adverse events	As required																						
Serious adverse events	As required																						
Comments	As required																						
Study completion information																							X

[redacted]

S = Information remains as source data

[1] Visit structure given for internal programming purposes only

[2] Assessments to performed pre-dose

[3] Daily dosing for 28 days

[4] PK sample to be drawn pre-dose

[5] PK sample to be taken 24 hours post dose on Day 28

[redacted]

Note: The intention was to have study visits during the dosing period 7 days apart, with permitted visit windows as outlined in the SOM. However it has been noted that the assessment schedule has the visit 3 labelled as Day 1 rather than Day 0 resulting in 6 days between this and the next visit. Since this error was noted after the study has started and patients dosed, the current visit names will remain unchanged, however sites should aim to schedule the visits 7 days apart, +/- the permitted visit window.

[redacted]

Table 8-2 Part 2 Assessment schedule

Study Phase	Screening	Dosing Period								EOS	Unsch-eduled
Visit Numbers	V1	V2			V3	V4	V5	V6	V7	V777	V999
**Weeks**	**-12 to -2**	**1**			**2**	**3**	**5**	**9**	**13**	**17**	**>21**
Study Day(s)	-84 to -14	1			8	15	29	57	85	113	>141
Time (post-dose)	-	Baseline	0h	2h		pre dose	pre dose	pre dose	24h[5]	-	-
Informed consent	X										
Inclusion/Exclusion Criteria	S										
Medical history	X	X									
Demography	X										
Alcohol Test and Drug Screen	S										
Physical Examination	S	S				S			S	S	
Hepatitis Screen	S										
HIV Screen	S										
Pregnancy test	X	X					X	X	X	X	
alpha-fetoprotein	X										
Blood type	X										
Drug dispensing			X				X	X			
Dose administration at site following completion of other assessments			X			X	X	X	X		
Body height	X										
Body weight	X	X				X	X	X	X	X	
Body temperature	X	X				X	X	X	X	X	
Pulse rate	X	X		X		X	X	X	X	X	
Blood pressure	X	X		X		X	X	X	X	X	
ECG evaluation	X	X		X		X	X	X	X	X	
Hematology	X	X				X	X	X	X	X	X
Blood chemistry	X	X				X	X	X	X	X	X
Urinalysis	X	X				X	X	X	X	X	X
Fasting lipid panel	X	X				X	X	X	X	X	
Liver function tests	X	X				X	X	X	X	X	X
PK blood collection		X		X		X	X	X	X	X	
[redacted]											
VAS (Visual Analog Scale)	X	X				X	X	X	X	X	
PBC40	X	X					X	X	X	X	
[redacted]											
Telephone follow up					X					X[4]	
Concomitant therapies	As required										
Adverse events	As required										
Serious adverse events	As required										
Comments	As required										
Study completion information										X	X

Key

S = Information remains as source data

EOS = End of Study visit

[redacted]

4 Telephone follow up call required 30 days post dose, if EoS visit not performed at this stage

5 To be scheduled approximately 24 hours post Day 84 dose

8.1 Informed consent procedures

Eligible subjects may only be included in the study after providing written (witnessed, where required by law or regulation), IRB/IEC-approved informed consent.

Informed consent must be obtained before conducting any study-specific procedures (i.e., all of the procedures described in the protocol). The process of obtaining informed consent should be documented in the subject source documents.

The date of signing of informed consent (and withdrawal, if later withdrawn) should be documented in the CRF.

Novartis will provide to investigators a proposed informed consent form that complies with the ICH GCP guideline and regulatory requirements and is considered appropriate for this study. Any changes to the proposed consent form suggested by the investigator must be agreed to by Novartis before submission to the IRB/IEC.

Pregnancy outcomes must be collected for the female partners of any males who took study treatment in this study. Consent to report information regarding these pregnancy outcomes should be obtained from the mother.

[redacted]

In the event that Novartis wants to perform testing on the samples that are not described in this protocol, additional Institutional Review Board and/or Ethics Committee approval will be obtained.

A copy of the approved version of all consent forms must be provided to the Novartis monitor after IRB/IEC approval.

8.2 Subject demographics/other baseline characteristics

Subject demographic and baseline characteristic data will be collected on all subjects.

Relevant medical history/current medical conditions data includes data until signature of informed consent. Where possible, diagnoses and not symptoms will be recorded.

Investigators have the discretion to record abnormal test findings on the medical history CRF whenever in their judgment, the test abnormality occurred prior to the informed consent signature.

8.3 Efficacy / Pharmacodynamics

Pharmacodynamic assessments are specified below, with the methods for assessment and recording specified in the Study Operations Manual. Assessments will be performed/samples collected at the timepoint(s) defined in the Assessment schedule.

In order to better define the PD profile, the timing of the sample collection may be altered based on emergent data. The number of samples/blood draws and total blood volume collected will not exceed those stated in the protocol.

8.3.1 Liver Function Tests

GGT, Albumin, AST, ALT, ALP, total bilirubin will be assessed.

The methods for assessment and recording are specified in the laboratory manual and SOM. Liver function tests may be completed as part of the blood chemistry panel.

8.3.2 Patient-Reported Outcome

PBC 40

The PBC-40 is a paper-based patient-derived, disease specific quality of life patient reported outcome (PRO) measure. It was developed and validated for use in subjects suffering from PBC (Jacoby et al 2005). It is designed for self-completion and takes approximately 5 minutes to complete. It consists of 40 questions arranged in 8 domains with between 3 and 11 questions in each domain. Each question is scored from 1 to 5 in increasing order of severity. The difference in total sum score between each treated group and placebo at Day 28 will be investigated.

PBC40 itch domain

The domain specifically relates to cholestatic itch symptomatology. In addition to the investigation of the total PBC-40 sum score, the sum scores from the 3 question itch sub-domain of the PBC40 questionnaire will be determined and used to test for an effect of LJN452 relative to placebo.

VAS for Itch and Sleep

The Global Itch Visual Analogue Scale, a 100 mm visual analogue scale (VAS) will be used to assess the severity of patients itch (ranging from 0 = none at all to 10 = the worst imaginable itch) and the Sleep Disturbance Visual Analogue Scale will be used to assess the impact of nocturnal itch on sleep (from 0 = no sleep loss to 10 = cannot sleep at all). The score (distance from left) on the VAS will be recorded for both parameters by the patient marking with a line and used to test for an effect of LJN452 over placebo.

8.4 Safety

Safety assessments are specified below; methods for assessment and recording are specified in the laboratory manual and Site Operations Manual, with the Assessment Schedule detailing when each assessment is to be performed.

8.4.1 Physical examination

Details on the physical examination will be provided in the Site Operations Manual.

8.4.2 Vital signs

- Body temperature
- Blood pressure (BP)
- Pulse

[redacted]

8.4.3 Height and weight

- Height
- Body weight
- Body mass index (BMI) will be calculated (Body weight (kg) / [Height (m)]^2)

8.4.4 Laboratory evaluations

Clinically relevant deviations of laboratory test results occurring during or at completion of the study must be reported and discussed with Novartis personnel. The results should be evaluated for criteria defining an adverse event and reported as such if the criteria are met. Repeated evaluations are mandatory until normalization of the result(s) or until the change is no longer clinically relevant. In case of doubt, Novartis personnel should again be contacted.

Hematology

Hemoglobin, hematocrit, red blood cell count, white blood cell count with differential and platelet count will be measured.

Clinical chemistry, including fasted lipid panel

Sodium, potassium, bicarbonate, creatinine, urea, uric acid, chloride, calcium, alkaline phosphatase, total bilirubin, LDH, GGT, AST, ALT, albumin, aPTT, PT/INR, CK, glucose, total cholesterol, HDL, LDL, triglycerides, magnesium, phosphate and vitamin D. If the total bilirubin concentration is increased above 1.5 times the upper limit of normal, direct and indirect reacting bilirubin should be differentiated.

Urinalysis

Urine test by dipstick e.g., Combur9: leucocytes, nitrite, pH, protein, glucose, ketones, urobilinogen, bilirubin, blood/ hemoglobin.

If the dipstick result is positive for protein, nitrite, leucocytes and/or blood, the sample will be sent for microscopic analysis of WBC, RBC and casts.

Special clinical laboratory evaluations

- Alpha-fetoprotein (performed only at screening)
- Blood type (performed only at screening)

Detailed information on special clinical laboratory assessments will be provided in the laboratory manual and the SOM.

8.4.5 Electrocardiogram (ECG)

PR interval, QRS duration, heart rate, RR, QT, QTc

The Fridericia QT correction formula (QTcF) should be used for clinical decisions.

[redacted]

8.4.6 Pregnancy

All pre-menopausal women who are not surgically sterile will have a serum pregnancy test at Screening followed by a urine pregnancy test at Baseline Visit before study drug administration. The urine pregnancy test will be repeated every four weeks up to the follow up visit (see Table 8-1 and Table 8-2). The tests will be performed at the clinical center.

A positive test at Screening and/or Baseline is an exclusion criterion for participating in the study. A positive urine pregnancy test after start of study drug requires immediate interruption of study drug until serum hCG is performed and found to be negative. If positive, the patient will enter the post-treatment follow up period. See also Section 9.5.

8.5 Pharmacokinetics

8.5.1 PK Blood collection and processing

A complete schedule of blood sampling for pharmacokinetics (including sampling time as sampling volumes) can be found in the blood log tables (provided in the SOM) while explicit details related to PK blood collection and processing, labeling, and shipment instructions, are located in the standalone central laboratory manual.

All samples will be given a unique sample number (as listed in the SOM). The actual sample collection date and time will be entered on the PK blood collection page of the eCRF. Sampling problems will be noted in the Comments page of the eCRFs.

8.5.2 Pharmacokinetic analytical method(s)

LJN452 will be determined in plasma using a validated LC-MS/MS method. [redacted]

[redacted]

[redacted]

[redacted]

[redacted]

[redacted]

[redacted]

[redacted]

[redacted]

9 Safety monitoring

9.1 Adverse events

An adverse event (AE) is any untoward medical occurrence (i.e., any unfavorable and unintended sign [including abnormal laboratory findings], symptom or disease) in a subject or clinical investigation subject **after providing written informed consent** for participation in the study. Therefore, an AE may or may not be temporally or causally associated with the use of a medicinal (investigational) product.

The occurrence of adverse events should be sought by non-directive questioning of the subject at each visit during the study. Adverse events also may be detected when they are volunteered by the subject during or between visits or through physical examination, laboratory test, or other assessments.

Abnormal laboratory values or test results constitute adverse events only if they fulfill at least one of the following criteria:

- they induce clinical signs or symptoms,
- they are considered clinically significant,
- they require therapy.

Clinically significant abnormal laboratory values or test results should be identified through a review of values outside of normal ranges/clinically notable ranges, significant changes from baseline or the previous visit, or values which are considered to be non-typical in patients with underlying disease. Investigators have the responsibility for managing the safety of individual subject and identifying adverse events. Alert ranges for liver related events are included in Section 9.3.

[redacted]

Adverse events must be recorded on the Adverse Events CRF for subjects that pass screening and enter into the study. The adverse events should be reported according to the signs, symptoms or diagnosis associated with them, and accompanied by the following information:

1. The Common Toxicity Criteria (CTC) AE grade (version 4). If CTC-AE grading does not exist for an adverse event, use:
   - 1=mild,
   - 2=moderate,
   - 3=severe
   - 4=life threatening.

   CTC-AE grade 5 (death) is not used, but is collected in other CRFs (e.g., Study Completion, Death/Survival).
2. its relationship to the study treatment (no/yes)
3. its duration (start and end dates) or if the event is ongoing an outcome of not recovered/not resolved should be reported.
4. whether it constitutes a serious adverse event (SAE) See Section 9.2 for definition of SAE
5. action taken regarding investigational treatment.

   All adverse events should be treated appropriately. Treatment may include one or more of the following:
   - no action taken (i.e., further observation only)
   - study treatment dosage adjusted/temporarily interrupted
   - study treatment permanently discontinued due to this adverse event
   - concomitant medication given
   - non-drug therapy given
   - subject hospitalized/subject's hospitalization prolonged

Once an adverse event is detected, it should be followed until its resolution or until it is judged to be permanent, throughout the study, and assessment should be made at each visit (or more frequently, if necessary) of any changes in severity, the suspected relationship to the study drug, the interventions required to treat it, and whether the AE is ongoing at the end of study visit.

Information about common side effects already known about the investigational drug can be found in the Investigator Brochure (IB) or Core Data Sheet (for marketed drugs) or will be communicated between IB updates in the form of Investigator Notifications. This information will be included in the subject informed consent and should be discussed with the subject during the study as needed.

The investigator should also instruct each subject to report any new adverse event (beyond the protocol observation period) that the subject, or the subject's personal physician, believes might reasonably be related to study treatment. This information should be recorded in the investigator's source documents, however, if the AE meets the criteria of an SAE, it must be reported to Novartis.

[redacted]

9.2 Serious adverse event reporting

9.2.1 Definition of SAE

An SAE is defined as any adverse event (appearance of (or worsening of any pre-existing) undesirable sign(s), symptom(s) or medical conditions(s)) which meets any one of the following criteria:

- is fatal or life-threatening
- results in persistent or significant disability/incapacity
- constitutes a congenital anomaly/birth defect
- requires inpatient hospitalization or prolongation of existing hospitalization, unless hospitalization is for:
  - routine treatment or monitoring of the studied indication, not associated with any deterioration in condition
  - elective or pre-planned treatment for a pre-existing condition that is unrelated to the indication under study and has not worsened since the start of study drug
  - treatment on an emergency outpatient basis for an event not fulfilling any of the definitions of a SAE given above and not resulting in hospital admission
  - social reasons and respite care in the absence of any deterioration in the patient's general condition
- is medically significant, i.e., defined as an event that jeopardizes the subject or may require medical or surgical intervention.

Life-threatening in the context of a SAE refers to a reaction in which the subject was at risk of death at the time of the reaction; it does not refer to a reaction that hypothetically might have caused death if more severe.

All malignant neoplasms will be assessed as serious under "medically significant" if other seriousness criteria are not met.

Any suspected transmission via a medicinal product of an infectious agent is also considered a serious adverse reaction.

Medical and scientific judgment should be exercised in deciding whether other situations should be considered serious reactions, such as important medical events that might not be immediately life threatening or result in death or hospitalization but might jeopardize the patient or might require intervention to prevent one of the other outcomes listed above. Examples of such events are intensive treatment in an emergency room or at home for allergic bronchospasm, blood dyscrasias or convulsions that do not result in hospitalization or development of dependency or abuse.

All AEs (serious and non-serious) are captured on the CRF, SAEs also require individual reporting to Chief Medical Office and Patient Safety as per Section 9.2.2.

[redacted]

9.2.2 SAE reporting

To ensure subject safety, every SAE, regardless of causality, occurring after the subject has provided informed consent and until 30 days after the last administration of study treatment must be reported to Novartis within 24 hours of learning of its occurrence as described below. Any SAEs experienced after this should only be reported to Novartis if the investigator suspects a causal relationship to study treatment.

Recurrent episodes, complications, or progression of the initial SAE must be reported as follow-up to the original episode, regardless of when the event occurs. This report must be submitted within 24 hours of the investigator receiving the follow-up information. An SAE that is considered completely unrelated to a previously reported one should be reported separately as a new event.

Information about all SAEs (either initial or follow up information*)* is collected and recorded on the paper Serious Adverse Event Report Form. The investigator must assess the relationship to each specific component of study treatment (if study treatment consists of several drugs) complete the SAE Report Form in English, and send the completed, signed form by fax within 24 hours after awareness of the SAE to the local Novartis Drug Safety and Epidemiology Department, notifying the Clinical Trial Leader. Contact information is listed in the Site Operations Manual.

The original copy of the SAE Report Form and the fax confirmation sheet must be kept with the source documentation at the study site. Follow-up information should be provided using a new paper SAE Report Form stating that this is a follow-up to a previously reported SAE.

Follow-up information provided should describe whether the event has resolved or continues, if and how it was treated, whether the treatment code was broken or not and whether the subject continued or withdrew from study participation. Each re-occurrence, complication, or progression of the original event should be reported as a follow-up to that event regardless of when it occurs.

If the SAE is not previously documented in the Investigator's Brochure or Package Insert (new occurrence) and is thought to be related to the investigational treatment a Drug Safety and Epidemiology Department associate may urgently require further information from the investigator for Health Authority reporting. Novartis may need to issue an Investigator Notification (IN) to inform all investigators involved in any study with the same investigational treatment that this SAE has been reported. Suspected Unexpected Serious Adverse Reactions (SUSARs) will be collected and reported to the competent authorities and relevant ethics committees in accordance with EU Guidance 2011/C 172/01 or as per national regulatory requirements in participating countries.

[redacted]

9.3 Liver safety monitoring

To ensure subject safety and enhance reliability in determining the hepatotoxic potential of an investigational drug, a standardized process for identification, monitoring and evaluation of liver events has to be followed.

The following two categories of abnormalities / adverse events have to be considered during the course of the study:

- Liver laboratory triggers, which will require repeated assessments of the abnormal laboratory parameter
- Liver events, which will require close observation, follow-up monitoring and completion of the standard base liver CRF pages

Please refer to Appendix 1 for complete definitions of liver laboratory triggers and liver events and their required follow up requirements.

These follow up actions may include:

- Repeating the LFT* to confirm elevation as defined in Table 15-1, interruption of dosing while result is pending
- Dose reduction (Part 2 only)
- Discontinuation of the investigational drug
- Hospitalization of the subject if appropriate
- A causality assessment of the liver event via exclusion of alternative causes (e.g., disease, co-medications)
- An investigation of the liver event which needs to be followed until resolution. These investigations can include serology tests, imaging and pathology assessments, hepatologist's consultancy, based on investigator's discretion.

*These LFT repeats should be performed using the central laboratory if possible. If this is not possible, then the repeats can be performed at a local laboratory to monitor the safety of the patient. Repeats laboratory should then be performed at central laboratory as soon as possible. All follow-up information, and the procedures performed should be recorded as appropriate in the CRF, using the Liver Event Checklist as guide, which should be kept as source data.

9.4 Renal safety monitoring

Renal events are defined in Appendix 1, with their required follow up requirements.

9.5 Pregnancy reporting

To ensure patient safety, each pregnancy in a subject on study drug must be reported to Novartis within 24 hours of learning of its occurrence. The pregnancy should be followed up to determine outcome, including spontaneous or voluntary termination, details of the birth, and the presence or absence of any birth defects, congenital abnormalities, or maternal and/or newborn complications. The study drug must be discontinued, though the subject may stay in the study, if she wishes to do so. All assessments that are considered as a risk during pregnancy must not be performed. The subject may continue all other protocol assessments.

[redacted]

Pregnancy must be recorded on a Pharmacovigilance Pregnancy Form and reported by the investigator to the local Novartis Drug Safety and Epidemiology Department. Pregnancy follow-up should be recorded on the same form and should include an assessment of the possible relationship to the Novartis study drug of any pregnancy outcome. Any SAE experienced during pregnancy must be reported on an SAE Report Form.

Pregnancy outcomes must be collected for the female partners of any males who took study treatment in this study. Consent to report information regarding these pregnancy outcomes should be obtained from the mother.

9.6 Early phase safety monitoring

The Investigator will monitor adverse events in an ongoing manner and inform the Sponsor of any clinically relevant observations. Any required safety reviews will be made jointly between medically qualified personnel representing the Sponsor and Investigator. Such evaluations may occur verbally, but the outcome and key discussion points will be summarized in writing (e-mail) and made available to both Sponsor and all Investigator(s).

The Sponsor will advise the Investigator(s) at all sites in writing (e-mail) (and by telephone if possible) of any new, clinically relevant safety information reported from another site during the conduct of the study in a timely manner.

10 Data review and database management

10.1 Site monitoring

Before study initiation, at a site initiation visit or at an investigator's meeting, a Novartis representative will review the protocol and CRFs with the investigators and their staff. During the study Novartis employs several methods of ensuring protocol and GCP compliance and the quality/integrity of the sites' data. The monitor will visit the site to check the completeness of subject records, the accuracy of entries on the CRFs, the adherence to the protocol and to Good Clinical Practice, the progress of enrollment, and to ensure that study drug is being stored, dispensed, and accounted for according to specifications. Key study personnel must be available to assist the monitor during these visits.

The investigator must maintain source documents for each subject in the study, consisting of case and visit notes (hospital or clinic medical records) containing demographic and medical information, laboratory data, electrocardiograms, and the results of any other tests or assessments. All information on CRFs must be traceable to these source documents in the subject's file. The investigator must also keep the original informed consent form signed by the subject (a signed copy is given to the subject).

The investigator must give the monitor access to all relevant source documents to confirm their consistency with the CRF entries. Novartis monitoring standards require full verification for the presence of informed consent, adherence to the eligibility criteria, documentation of SAEs, and the recording of data that will be used for all primary and safety variables. Additional checks of the consistency of the source data with the CRFs are performed according to the study-specific monitoring plan. No information in source documents about the identity of the subjects will be disclosed.

[redacted]

10.2 Data collection

Designated investigator staff will enter the data required by the protocol into the Electronic Case Report Forms using fully validated software that conforms to 21 CFR Part 11 requirements. Designated investigator site staff will not be given access to the EDC system until they have been trained. Automatic validation programs check for data discrepancies and, by generating appropriate error messages, allow the data to be confirmed or corrected before transfer of the data to the CRO working on behalf of Novartis. The Investigator must certify that the data entered into the Electronic Case Report Forms are complete and accurate. After final database lock, the investigator will receive a CD-ROM or paper copies of the subject data for archiving at the investigational site.

Data not requiring a separate written record will be defined in the Site Operations Manual and assessment schedule and can be recorded directly on the CRFs. All other data captured for this study will have an external originating source (either written or electronic) with the CRF not being considered as source.

All data should be recorded, handled and stored in a way that allows its accurate reporting, interpretation and verification.

CRO working on behalf of Novartis review the data entered into the eCRFs by investigational staff for completeness and accuracy and instruct the site personnel to make any required corrections or additions. Queries are sent to the investigational site using an electronic data query. Designated investigator site staff is required to respond to the query and confirm or correct the data. If the electronic query system is not used, a paper Data Query Form will be faxed to the site. Site personnel will complete and sign the faxed copy and fax it back to the CRO working on behalf of Novartis who will make the correction to the database.

Concomitant medications entered into the database will be coded using the WHO Drug Reference List, which employs the Anatomical Therapeutic Chemical classification system. Medical history/current medical conditions and adverse events will be coded using the Medical dictionary for regulatory activities (MedDRA) terminology.

Laboratory samples will be processed centrally and the results will be sent electronically to Novartis (or a designated CRO).

Randomization codes will be tracked using IRT for Part 2. The system will be supplied by a vendor, who will also manage the database. The database will be sent electronically to Novartis (or a designated CRO).

This study will utilize a staggered lock of the clinical database, so that the data can be locked after completion of Part 1, or set of Cohorts in Part 1.

At the conclusion of Part 1 of the study or lock of a set of Cohorts from Part 1, in the event of a staggered lock, the occurrence of any emergency code breaks will be determined after return of all code break reports and unused drug supplies to Novartis.

For Part 2, each occurrence of a code break via IRT will be reported to the clinical team and monitor. The code break functionality will remain available until study shut down or upon request of Novartis.

[redacted]

The occurrence of any protocol deviations will be determined. After these actions have been completed and the database has been declared to be complete and accurate, it will be locked and the treatment codes will be unblinded and made available for data analysis. Any changes to the database after that time can only be made by joint written agreement by Novartis management.

All actions described above to lock the database apply to a lock occurring after completion of Part 1 (or Cohorts from Part 1) and at the end of the study. Once all necessary actions for database lock on the relevant data have been completed and the relevant data has been declared to be complete and accurate, it will be locked and the relevant treatment codes will be released and made available for data analysis. Any changes to the relevant data after that time can only be made by joint written agreement by Novartis management.

[redacted]

10.3 Data Monitoring Committee

A Data Monitoring Committee (DMC) has been assembled for this compound to monitor safety data. This committee is comprised of Novartis personnel not directly involved with the study. The DMC will convene as outlined in the DMC Charter.

Further, the DMC will review available data from Part 1 and approve the planned doses for Part 2 prior to the doses being confirmed.

The DMC will review safety, including AEs and laboratory parameters, on a regular basis. In addition, in the event that more than 3 patients develop an AE of CTCAE grade 3 or higher in the same system organ class, the DMC Chair will be alerted. Further details regarding relevant data and actions will be specified in the separate DMC charter. In addition, adverse events, including clinical laboratory data will be continuously reviewed by the Novartis medical monitor and as indicated, by the Novartis pharmacovigilance team.

10.4 Adjudication Committee

Not required.

[redacted]

11 Data analysis

11.1 Analysis sets

For all analysis sets, subjects will be analyzed according to the study treatment(s) received. Subjects with dose reduction due to ALT/AST will be analyzed according to the treatment they received up to the dose reduction in Part 2.

The safety analysis set will include all subjects that received any study drug.

The PK analysis set will include all subjects with at least one available valid (i.e., not flagged for exclusion) PK concentration measurement, who received any study drug and experienced no protocol deviations with relevant impact on PK data.

The PD analysis set will include all subjects with available PD data and no protocol deviations with relevant impact on PD data.

Details of protocol deviations which warrant exclusion from either the PK or PD analyses data sets will be provided in the RAP document Module 3.

11.2 Subject demographics and other baseline characteristics

All data for background and demographic variables will be listed by treatment group and subject. Summary statistics will be provided by treatment group.

Relevant medical history, current medical conditions, results of laboratory screens, drug tests and any other relevant information will be listed by treatment group and subject.

11.3 Treatments (study drug, rescue medication, other concomitant therapies, compliance)

Data for study drug administration (rescue medication) and concomitant therapies will be listed by treatment group and subject.

11.4 Analysis of the primary variable(s)

The primary efficacy objective is to evaluate the treatment effect of LJN452 over placebo in terms of reduction in cholestasis. The primary efficacy endpoint is the fold change in serum gamma-glutamyl transferase (GGT) from baseline to Day 28 and Day 85 for Part 1 and Part 2, respectively.

11.4.1 Variable(s)

The primary efficacy endpoint is the fold change in serum gamma-glutamyl transferase (GGT) from baseline. The Serum GGT values at all time points will be logarithmically transformed prior to analysis. The rationale for the log transformation is to achieve more approximate normality and to be able to estimate a treatment effect on a ratio scale. Y=log(GGT+0.001) will be considered only if there are many observations with GGT result as zero.

[redacted]

11.4.2 Statistical model, hypothesis, and method of analysis

Changes from baseline for log transformed serum GGT will be analyzed by repeated measures analysis of covariance (ANCOVA) with a fixed effect for the treatment, visit, treatment*visit interaction, log-transformed baseline as well as log(baseline)*visit interaction. The UDCA and pruritus stratification factor will also be included as a fixed effect in the ANCOVA model for Part 2 analysis. A saturated covariance structure will be used for observations within the same subject. The model will be fitted using SAS Proc Mixed and the restricted maximum likelihood scoring method (reml). Two-sided 90% confidence intervals for the difference between each LJN452 dose and placebo will be evaluated for each visit. This will then be "back-transformed" to the original scale to give a fold change from baseline. Dose response may be assessed using appropriate contrasts. The analysis at the 28 day visit and 85 day visit will constitute the primary analysis for Part 1 and Part 2, respectively. The analysis will be done for each study part using pooled placebo data within parts 1 and 2, respectively. No adjustment for multiplicity will be done. [redacted]

Similar analysis as mentioned above can be performed for key LFT parameters.

Additionally a repeated measures ANCOVA treating all post dose reduction measurements as missing will be performed as well.

11.4.3 Handling of missing values/censoring/discontinuations

All missing GGT values will be considered missing at random. No imputation or adjustment to the primary analysis will be done.

11.4.4 Supportive analyses

If it turns out that the primary analysis cannot be fitted because data cannot support a saturated model, a simpler covariance matrix such as the compound symmetric covariance structure may be fitted.

11.5 Analysis of secondary [redacted] variables

11.5.1 Efficacy / Pharmacodynamics

The secondary efficacy/PD variables are the PBC-40 total score, the PBC-40 itch domain subscore, and the VAS scale PRO as well as ALP.

The difference between LJN452 and placebo will be assessed by Wilcoxon rank sum test for change from baseline in total PBC score and itch subdomain score at each visit. A two-sided p-value for a treatment difference will be reported with no adjustment for multiplicity. In addition, summary statistics (mean median std min max) will be reported at each applicable time point.

[redacted]

As an additional secondary end point, a 100 mm visual analogue scale (VAS) will be used to assess the severity of patients itch as well as loss of sleep. The score (distance from left) on the VAS will be recorded for both parameters by the patient marking with a line. The distance marked will be converted to a score between 0 and 10.

The change from baseline VAS scores for itch and sleep loss will be analyzed by analysis of covariance with a baseline covariate, a fixed effect for treatment, visit, and treatment by visit interaction as well as visit by baseline interaction. An unstructured covariance matrix will be used 90% two-sided confidence intervals for the difference between each LJN452 dose and placebo will be presented. The UDCA and pruritus stratification factor will also be included as a fixed effect in the model for Part 2 analysis.

ALP data will be analyzed using the same approach as described in Section 11.4.2. Additionally the number and percentage of subjects who have ALP<1.67xULN with > 15% reduction from baseline and a normal total bilirubin level will be tabulated by part, treatment and visit.

11.5.2 Safety

Vital signs

All vital signs data will be listed by part, treatment, subject, and visit/time and if ranges are available abnormalities (and relevant orthostatic changes) will be flagged. Summary statistics will be provided by part, treatment and visit/time.

ECG evaluations

All ECG data will be listed by part, treatment, subject and visit/time, abnormalities will be flagged. Summary statistics will be provided by treatment and visit/time.

Clinical laboratory evaluations

All laboratory data will be listed part, by treatment, subject, and visit/time and if normal ranges are available abnormalities will be flagged. Summary statistics will be provided by treatment and visit/time.

Adverse events

All information obtained on adverse events will be displayed by treatment and subject.

The number and percentage of subjects with adverse events will be tabulated by body system and preferred term with a breakdown by treatment. A subject with multiple adverse events within a body system is only counted once towards the total of this body system.

[redacted]

Other safety evaluations

Not applicable.

Immunogenicity

Not applicable.

11.5.3 Pharmacokinetics

11.5.3.1 Pharmacokinetic parameters

For standard pharmacokinetic abbreviations and definitions see the list provided at the beginning of this protocol.

All subjects with at least one period of evaluable pharmacokinetic (PK) parameter data will be included in the pharmacokinetic data analysis.

In Part 1, the pharmacokinetic parameters include (but are not limited to) AUC, Cmax, Tmax for LJN452.

Plasma concentrations will be expressed in ng/mL. All concentrations below the limit of quantification or missing data will be labeled as such in the concentration data listings. Concentrations below the Limit of Quantification will be treated as zero in summary statistics for concentration data only. They will not be considered for calculation of PK parameters (with the exception of the pre-dose samples).

Pharmacokinetic parameters will be determined using the actual recorded sampling times and non-compartmental method(s) with Phoenix (Version 6.2 or higher).

11.5.3.2 Statistical methods for pharmacokinetic analyses

Descriptive statistics of pharmacokinetic parameters will include mean, SD, and CV, min and max. When a geometric mean will be presented it will be stated as such. Since Tmax is generally evaluated by a nonparametric method, median values and ranges will be given for this parameter.

At day 1 and Day 28, AUC and Cmax will be summarized for each cohort along with 90% two-sided confidence intervals computed on the log-scale and back-transformed. All subjects with available PK parameters and no protocol deviation excluding them from the PK analysis set will be part of this computation.

If 3 or more cohorts are included in part 1 of the study, then dose-proportionality will be explored for PK parameters (AUCtau and Cmax) by fitting a power model (PK parameter = α dose $^{\beta}$). A natural logarithmic transformation will be applied for the power model, which implies to a general linear model to the log transformed PK parameter with a fixed continuous log-dose effect. The model becomes log (PK parameter) = $\log(\alpha) + \beta \log(\text{Dose}) + \varepsilon$. The estimate of the common slope β, which is the exponent in the power model, and the 90% confidence interval of the exponent will be obtained. Dose proportionality will be considered if the 90% confidence interval for β is completely contained within the pre-specified critical region (bL, bU).

[redacted]

LJN452 plasma concentration data will be listed by treatment, subject, and visit/sampling time point. Descriptive summary statistics will be provided by treatment and visit/sampling time point. Additionally summary statistics may also be provided for the LJN452 plasma concentration data with a breakdown by UDCA stratum as appropriate.

Summary statistics will include mean (arithmetic and geometric), SD, CV (arithmetic and geometric), median, minimum and maximum. An exception to this is Tmax where median, minimum and maximum will be presented. Concentrations below LLOQ will be treated as zero in summary statistics and for PK parameter calculations. A geometric mean will not be reported if the dataset includes zero values. Pharmacokinetic parameters will be calculated as described in Section 8.5 and will be listed by treatment and subject.

11.5.4 Pharmacokinetic / pharmacodynamic interactions

The relationship between PK and key primary and/or secondary PD parameters will be explored. [redacted]

[redacted]

11.6 Sample size calculation

A recently published study of a compound with similar mechanism (Mason et al 2010b; Hirschfield et al 2015) on a similar population (study of INT-474 in combination with UDCA in patients with PBC) suggests that the treatment difference over placebo at Day 28 is approximately -50% with a standard deviation ~ 0.28. In Part 1, with 12 patients per cohort randomized to LJN452 or placebo with a randomization ratio of 2:1, there is approximately 85% probability that the upper limit of a 90% two-sided confidence interval of treatment difference between LJN452 versus placebo will exclude 0, if the underlying treatment difference is about -50% in terms of percent change from baseline. A total of up to 5 cohorts of LJN452 at different dose levels may be studied. Assuming a dropout rate of approximately 20%, 15 patients per cohort will be enrolled in Part 1, with a total of at least 30 (up to 75) patients.

Part 2 of the study will be conducted on patients currently taking UDCA and UDCA naïve patients. A published study (Jones et al 2011) (study of INT-474 as monotherapy in patients with PBC) suggests that the effect size of change from baseline in GGT is bigger in naïve patients than in the non-responder population studied in Part 1. So, we can assume a treatment effect of at least 50% as well as a standard deviation of 0.28. Based on power calculation for Part 1, enrolling at least 30 patients to receive an LJN452 dose and 20 patients to receive the placebo treatment in Part 2 will provide at least 95% power that a two-sided significance test at nominal level 5% will detect a difference (same analysis as in Part 1). A total of 80 patients are planned to be randomized to LJN452 or placebo with a 3:3:2 ratio (Dose Level 1:Dose Level 2:Placebo). Assuming a dropout rate of approximately 10%, 88 patients will be enrolled in Part 2.

11.7 Power for analysis of key secondary variables

Data from 30 patients receiving each LJN452 dose and 20 patients receiving placebo in Part 2 will provide 81% power to detect a 15% treatment difference in change from baseline ALP between an LJN452 dose and placebo, based on a 2-sided t-test at the 0.05 significance level assuming a standard deviation of 17.8%, obtained from the interim analysis of the first 2 cohorts in Part 1.

11.8 Interim analyses

Planned interim analyses will take place in Parts 1 and 2 of the study.

Planned interim analyses will be performed in Part 1; a) when at least 12 patients in Cohort 1 have completed 4 weeks of dosing and have finished the day 28 post dose assessments; b) when the same time point is reached for at least 12 subjects in each cohort of Part 1.

The purpose of the first interim analysis is to assess patient safety, to evaluate the assumption on intra-subject variability of GGT which was used for sample size calculations, and to select a dose for the next cohort. An ANCOVA model will be fitted to estimate the change from baseline in GGT to day 28 and its variability, and an unblinded sample size re-estimation conducted. The results may be used to adjust the size of the next cohort.

[redacted]

In the subsequent interim analyses in Part 1, placebo data from all cohorts will be pooled in the ANCOVA model to evaluate the treatment difference between each dose level of LJN452 versus placebo. The model described in the primary analysis section will be fitted, as it is indeed the same analysis which will be included in the final study report.

Unblinded interim analysis results from Part 1 will be reviewed by an internal Novartis Interim Analysis Team which may include some CTT members. The Interim Analysis Team may communicate interim results to relevant Novartis teams for information, consulting and/or decision purposes. The composition of the interim analysis team will be decided before the FPFV and a roster of membership maintained in the trial master file.

The Interim Analysis Team may communicate interim results (e.g., evaluation of PoC criteria or information needed for planning/modifying another study) to relevant Novartis teams for information, consulting and/or decision purposes.

No further dissemination of interim results based on un-locked data should occur, in particular not with individuals involved in treating the study's subjects or assessing clinical data (e.g., ECGs, images, symptoms) obtained in the study.

In Part 2 of the study an interim analysis is planned when approximately 40 patients have completed their Week 8 visit. The purpose of this interim analysis is to review the sample size (calculated based on GGT and ALP changes) to assess if it is also adequate to provide precise enough treatment difference estimates in itch parameters, a key tolerability endpoint. A blinded sample size re-estimation will be performed and a proposal to increase the sample size submitted to the DMC as needed. The DMC will decide whether it is appropriate to increase the sample based on an unblinded interim analysis, which will be performed by a study independent statistician and programmer with outputs provided to the DMC only. Recruitment will continue while the IA is being performed.

11.9 Interim analyses on locked data

This study will utilize a staggered lock of the clinical database, so that the data can be locked after completion of Part 1, or a set of Cohorts in Part 1.

Once a Cohort's data has been locked, treatment codes for the relevant cohort(s) will be released and made available for data analysis.

The results from the locked interim analysis can be communicated beyond Novartis to groups including, but not limited to individuals treating the study's subjects, Health Authorities and on clinical registries.

[redacted]

12 Ethical considerations

12.1 Regulatory and ethical compliance

This clinical study was designed and shall be implemented and reported in accordance with the ICH Harmonized Tripartite Guidelines for Good Clinical Practice, with applicable local regulations (including European Directive 2001/20/EC, US Code of Federal Regulations Title 21, and Japanese Ministry of Health, Labor, and Welfare), and with the ethical principles laid down in the Declaration of Helsinki.

12.2 Responsibilities of the investigator and IRB/IEC

Before initiating a trial, the investigator/institution should obtain approval/favorable opinion from the Institutional Review Board/Independent Ethics Committee (IRB/IEC) for the trial protocol, written informed consent form, consent form updates, subject recruitment procedures (e.g., advertisements) and any other written information to be provided to subjects. Prior to study start, the investigator is required to sign a protocol signature page confirming his/her agreement to conduct the study in accordance with these documents and all of the instructions and procedures found in this protocol and to give access to all relevant data and records to Novartis monitors, auditors, Novartis Quality Assurance representatives, designated agents of Novartis, IRBs/IECs, and regulatory authorities as required. If an inspection of the clinical site is requested by a regulatory authority, the investigator must inform Novartis immediately that this request has been made.

For multi-center trials, a Coordinating Investigator will be selected by Novartis around the time of Last Patient Last Visit to be a reviewer and signatory for the clinical study report.

12.3 Publication of study protocol and results

Novartis assures that the key design elements of this protocol will be posted in a publicly accessible database such as clinicaltrials.gov. In addition, upon study completion and finalization of the study report the results of this trial will be either submitted for publication and/or posted in a publicly accessible database of clinical trial results.

13 Protocol adherence

This protocol defines the study objectives, the study procedures and the data to be collected on study participants. Additional assessments required to ensure safety of subjects should be administered as deemed necessary on a case by case basis. Under no circumstances should an investigator collect additional data or conduct any additional procedures for any research related purpose involving any investigational drugs.

Investigators must apply due diligence to avoid protocol deviations. If the investigator feels a protocol deviation would improve the conduct of the study this must be considered a protocol amendment, and unless such an amendment is agreed upon by Novartis and approved by the IRB/IEC/REB it cannot be implemented. All significant protocol deviations will be recorded and reported in the CSR.

13.1 Protocol Amendments

Any change or addition to the protocol can only be made in a written protocol amendment that must be approved by Novartis, Health Authorities where required, and the IRB/IEC prior to implementation.

Only amendments that are intended to eliminate an apparent immediate hazard to subjects may be implemented, provided the Health Authorities and the reviewing IRB/IEC are subsequently notified by protocol amendment.

Notwithstanding the need for approval of formal protocol amendments, the investigator is expected to take any immediate action required for the safety of any subject included in this study, even if this action represents a deviation from the protocol. In such cases, the CTL should be informed and (serious) adverse event reporting requirements (Section 9) followed as appropriate.

[redacted]

14 References

Available upon request.

Calkin AC, Tontonoz P (2012) Transcriptional integration of metabolism by the nuclear sterol-activated receptors LXR and FXR. Nat Rev Mol Cell Biol; 13(4):213-24.

Carbone M, Mells G, Pells G, et al (2013) Sex and age are determinants of the clinical phenotype of Primary Biliary Cirrhosis and response to Ursodeoxycholic acid. Gastroenterology; 144:560-569

[redacted]

Chen F, Ananthanarayanan M, Emre S, et al (2004) Progressive familial intrahepatic cholestasis, type 1, is associated with decreased farnesoid X receptor activity. Gastroenterology; 756-64.

Hirschfield G, Mason A, Luketic V, et al (2015) Efficacy of obeticholic acid in patients with primary biliary cirrhosis and inadequate response to ursodeoxycholic acid. Gastroenterolgy; 148(4), 751-761.

Jacoby A, Rannard A, Buck D, et al (2005) Development, validation, and evaluation of the PBC-40, a disease specific health related quality of life measure for primary biliary chirrhosis. Gut; 54(11): 1622-1629

Jones D, Kowdley K, Chapman R, et al (2011) The first new monotherapy therapeutic PBC study in a decade? An interventional study evaluating the farnesoid X receptor agonist obeticholic acid in PBC. Gut; 60 (Suppl 2).

Kowdley KV, Luketic VA, Jones DE, et al (2011) The first new monotherapy therapeutic PBC study in a decade? An international study evaluating the farnesoid X receptor agonist obeticholic acid in PBC. Hepatology; (54): 416A-417A.

Makishima M, Okamoto AY, Repa JJ, et al (1999) Identification of a nuclear receptor for bile acids. Science; 1362-5.

Mason AL, Luketic VA, Lindor KD, et al (2010a) Farnesoid-x receptor agonists: A new class of drugs for the treatment of PBC? An international study evaluating the addition of obeticholic acid (INT-747) to ursodeoxycholic acid. Hepatology; 52 (357A).

Mason AL, Luketic VA, Lindor KD, et al (2010b) Farnesoid-x receptor agonists: A new class of drugs for the treatment of PBC? An interventional study evaluating the addition of INT-747 to ursodeoxycholic acid. Journal of Hepatology; 52 S1-S21.

Nevens F, Andreone P, Mazzella G, et al (2016) A placebo-controlled trial of obeticholic acid in primary biliary cholangitis. N Engl J Med; 375(7):631-43.

Parks DJ, Blanchard SG, Bledsoe RK, et al (1999) Bile acids: natural ligands for an orphan nuclear receptor. Science; 1365-8.

[redacted]

Treinen-Moslen M, Kanz MF (2006) Intestinal tract injury by drugs: Importance of metabolite delivery by yellow bile road. Pharmacol Ther;112(3):649-67.

Wang H, Chen J, Hollister K, et al (1999) Endogenous bile acids are ligands for the nuclear receptor FXR/BAR. Mol Cell; 543-53.

Zollner G, Wagner M, Moustafa T, et al (2006) Coordinated induction of bile acid detoxification and alternative elimination in mice: role of FXR-regulated organic solute transporter-alpha/beta in the adaptive response to bile acids. Am J Physiol Gastrointest Liver Physiol;290(5):G923-3.

[redacted]

15 Appendix 1: Liver and Renal events definitions and required actions

The following tables define Liver and Renal events in the study that require specific follow up and further action. These actions include discontinuation of study drug, reduction in dose, a dose interruption and increased frequency of safety monitoring. Please see Table 15-1 (Liver events) and Table 15-2 (Renal events) for further information.

[redacted]

Table 15-1 Liver Triggers and Events: definition and Follow-up Requirements

Criteria	First Action	Result	Second Action	Result	Third Action
**ALT or AST**					
**Potential Hy's Law case** defined as: **ALT or AST > 3 × ULN and** **TBL > 2 × ULN without notable increase in ALP to > 2 × ULN**	• **Discontinue study drug**<br>• Hospitalize if clinically appropriate<br>• Establish causality and complete liver event checklist (in SOM)	--	Continue monitoring with Repeat tests[a] until resolution [c]	--	--
**ALT or AST** **> 3 × ULN** **and INR > 1.5**	• **Discontinue study drug**<br>• Hospitalize, if clinically appropriate<br>• Establish causality and complete liver event checklist (in SOM)	--	Continue monitoring with Repeat tests[a] until resolution [c]	--	--
**ALT or AST** **> 8 × ULN**	• **Discontinue study drug**<br>• Hospitalize if clinically appropriate<br>• Establish causality and complete liver event checklist (in SOM)	--	Continue monitoring with Repeat tests[a] until resolution [c]	--	--
**ALT or AST** **> 2 X Baseline** **AND** **> 3 × ULN** **AND accompanied by symptoms[b]**	• **Discontinue study drug**<br>• Hospitalize if clinically appropriate<br>• Establish causality and complete liver event checklist (in SOM)	--	Continue monitoring Repeat tests[a] until resolution [c]	--	--

[redacted]

Criteria	First Action	Result	Second Action	Result	Third Action
**ALT or AST**<br>**> 2 X Baseline**<br>**AND**<br>**> 5 to ≤ 8 × ULN**	• Repeat tests[a] within 48 hours of notification of result<br>• **HOLD study drug** until repeat test results available<br>• Establish causality and complete liver event checklist (in SOM)	⬆	**Part 1**<br>• **<u>Discontinue</u> study drug**		• Continue monitoring with Repeat tests[a] 2-3 times per week until resolution [c]<br>• Update liver event checklist
			**Part 2**<br>• **Reduce dose** (Table 6-1).<br>• Restart study drug at reduced dose<br>• Repeat tests[a] 2-3 times a week after re-starting study drug<br>• Update liver event checklist (in SOM)	⬆	**Part 2**<br>**If elevated after re-starting study drug, discontinue study drug**<br>• Continue monitoring with Repeat tests[a] until resolution [c]<br>• Update liver event checklist
				⬇	**If values normalize, continue with reduced dose.**<br>• Repeat tests[a] two and three weeks after re-starting study drug<br>**If levels remain ≥ 2x Baseline 3 weeks after dose reduction, <u>discontinue study drug</u>**<br>• Continue monitoring with Repeat tests[a] until resolution [c]<br>• Update liver event checklist

Criteria	First Action	Result	Second Action	Result	Third Action
		⬇	**Part 1**<br>• Restart study drug at assigned dose level<br>• Repeat tests[a] 2-3 times a week after re-starting study drug<br>• Update liver event checklist<br>**Part 2**<br>• **Maintain assigned dose level**<br>• Restart study drug at assigned dose level<br>• Repeat tests[a] 2-3 times a week after re-starting study drug<br>• Update liver event checklist	⬆	**Both Parts**<br>**If elevated after re-starting study drug, discontinue study drug**
				⬇	**Both Parts**<br>• Continue study drug at assigned dose level
**ALT or AST**<br>**> 2 X Baseline**<br>**AND**<br>**≤ 5 × ULN**<br>(patient is asymptomatic)	• Repeat tests[a] within 48 hours of notification of result	⬆	**Part 1**<br>• **<u>Discontinue</u> study drug**		**Part 1**<br>• Continue monitoring with Repeat tests[a] 2-3 times per week until resolution [c]<br>• Update liver event checklist
			**Part 2**<br>• Repeat tests[a] visit within 48 hours<br>• **HOLD study drug** until repeat test results available<br>• Complete liver event checklist	⬆	**Part 2**<br>**A. Reduce dose** (Table 6-1).<br>• Restart study drug at reduced dose<br>• Repeat tests[a] 2-3 times a week after re-starting study drug<br>**B. If elevated after re-starting study drug, discontinue study drug**<br>• Continue monitoring with Repeat tests[a] until resolution [c]<br>• Update liver event checklist

Criteria	First Action	Result	Second Action	Result	Third Action
				↓	**Part 2**<br>• **Maintain assigned dose level**
		↓	**Both Parts**<br>• **Maintain assigned dose level**		
**Alkaline phosphatase**					
**ALP (isolated)**<br>**> 2 × ULN if normal baseline or**<br>**>2 x baseline if elevated before drug exposure (in the absence of known bone pathology)**	• Repeat tests[a] within 48 hours of notification of result	↑	**Both Parts**<br>• Monitor LFT within 1 to 4 weeks or at next visit<br>• Establish causality and complete liver event checklist (in SOM)		-
**Total bilirubin**					
**> 2 × ULN if normal baseline or**<br>**>2 x baseline if elevated before drug exposure**<br>**(in the absence of known Gilbert syndrome)**	• Repeat tests[a] within 48 hours of notification of result<br>• Test for hemolysis (e.g., reticulocytes, haptoglobin, unconjugated [indirect] bilirubin)	↑	**Both Parts**<br>• **Discontinue study drug**<br>• Initiate close monitoring (evaluate 2-3 times a week with repeat tests[a])<br>• Hospitalize if clinically appropriate<br>• Establish causality and complete liver event checklist (in SOM)		**Both Parts**<br>• Continue monitoring with Repeat tests[a] until resolution [c]
**> 1.5 to ≤ 2 × ULN**<br>**(patient is asymptomatic)**	• Repeat tests[a] within a week of notification of result	↑	**Both Parts**<br>If elevation from baseline is confirmed, initiate close observation of the patient i.e. evaluate 2-3 times a week with repeat tests[a]		**Both Parts**<br>• Repeat tests[a] within 1 to 4 weeks or at next visit

[redacted]

Criteria	First Action	Result	Second Action	Result	Third Action
**Jaundice**	• **Discontinue study drug**<br>• Initiate close monitoring (evaluate 2-3 times a week with repeat tests[a])<br>• Hospitalize if clinically appropriate<br>• Establish causality and complete liver event checklist (in SOM)		**Both Parts**<br>• Continue monitoring with Repeat tests[a] until resolution [c]		
**Any AE potentially indicative of a liver toxicity***	• Consider study drug interruption or discontinuation<br>• Hospitalization if clinically appropriate<br>• Establish causality and complete liver event checklist (in SOM)		**Both Parts**<br>Investigator discretion		

*These events cover the following: hepatic failure, fibrosis and cirrhosis, and other liver damage-related conditions; the non-infectious hepatitis; the benign, malignant and unspecified liver neoplasms

[a] Repeat tests: Physical examination and PT/INR and LFT: Liver function test: ALT, AST, GGT, alkaline phosphatase, total bilirubin, and albumin (see Section 6.2.1);

[b](General) malaise, fatigue, abdominal pain, nausea, or vomiting, or rash with eosinophilia

[c] Resolution is defined as an outcome of one of the following: (1) return to baseline values, (2) stable values at three subsequent monitoring visits at least 2 weeks apart, (3) remain at elevated level after a maximum of 6 months, (4) liver transplantation, and (5) death.

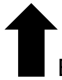 Elevation confirmed or persisting, i.e. remain > 90% of the original elevated level

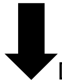 Decreasing elevation, i.e. decrease to ≤ 90 % of the original elevated level.

Table 15-2 Renal events: definition and follow up requirements

Renal Event	Actions
Serum creatinine ≥ 2 x BL or ≥1.5 mg/dL	**1. Hold study medication**<br>**2.** Confirm increase after 24-48h of notification of the value<br>**3.** If confirmed, **permanently discontinue study medication**<br>**4.** Follow up until resolution or stabilization of laboratory abnormality(ies)
Serum creatinine increase<br>25 – 49% compared to baseline during normal hydration status	Confirm 25% increase after 24-48h of notification of the value<br>Follow up within 2-5 days
Serum creatinine increase ≥ 50 % compared to baseline	Confirm ≥ 50 % within 24-48 hours after receipt of the abnormal value<br>If confirmation of abnormal value is not possible in this timeframe study drug must be withheld until further evaluation is possible<br>Consider drug interruption<br>Consider patient hospitalization / specialized treatment
Albumin- or Protein-creatinine ratio increase ≥ 2-fold compared to baseline<br>Albumin-creatinine ratio (ACR) ≥ 30 mg/g or ≥ 3 mg/mmol;<br>New dipstick proteinuria ≥ 1+<br>Protein-creatinine ratio (PCR) >ULN	Confirm value after 24-48h of notification of the value<br>Perform urine microscopy<br>Consider drug interruption / discontinuation
New dipstick glucosuria ≥ 1+ not due to diabetes	Blood glucose (fasting)<br>Perform serum creatinine, ACR
New dipstick hematuria not due to trauma	Urine sediment microscopy<br>Perform serum creatinine, ACR
Document contributing factors: co-medication, other co-morbid conditions, and additional diagnostic procedures performed in the CRF	
Monitor patient regularly (frequency at investigator's discretion) until one of the following:<br>Event resolution: (sCr within 10% of baseline or protein-creatinine ratio within 50% of baseline)<br>Event stabilization: sCr level with ±10% variability over last 6 months or protein-creatinine ratio stabilization at a new level with ±50% variability over last 6 months.	

[redacted]

16 Appendix 2: Pruritus event definitions and required actions

The following table defines options for management of different CTCAE grades of pruritus. These include: concomitant medication administration; study drug interruption or withdrawal of study drug.

Table 16-1 Pruritus event definitions and required actions

Pruritus Event	Actions
**CTCAE Grade 1**<br>Mild or localized; topical intervention indicated	Treat with concomitant medication according to Investigator's discretion<br>No option for drug interruption
**CTCAE Grade 2**<br>Intense or widespread; intermittent; skin changes from scratching (e.g., edema, papulation, excoriations, lichenification, oozing/crusts); oral intervention indicated; limiting instrumental ADL	Treat with concomitant medication according to Investigator's discretion<br>No option for drug interruption
**CTCAE Grade 3**<br>Intense or widespread; constant; limiting self-care ADL or sleep; oral corticosteroid or immunosuppressive therapy indicated	Treat with concomitant medication according to Investigator's discretion<br>Option for drug interruption of up to 1 week

[redacted]
