# Supplementary material for: Farnesoid X receptor agonist tropifexor attenuates cholestasis in a randomised trial in patients with primary biliary cholangitis
Source: JHEP Rep. 2022 Jul 21;4(11):100544. doi: 10.1016/j.jhepr.2022.100544 (PMC9576902; doi:10.1016/j.jhepr.2022.100544)
Supplement: Multimedia component 5 [file mmc5.docx]

**JHEP Reports**

**CTAT methods**

Tables for a “Complete, Transparent, Accurate and Timely account” (CTAT) are now mandatory for all revised submissions. The aim is to enhance the reproducibility of methods.

- Only include the parts relevant to your study
- Refer to the CTAT in the main text as ‘Supplementary CTAT Table’
- Do not add subheadings
- Add as many rows as needed to include all information
- Only include one item per row

**If the CTAT form is not relevant to your study, please outline the reasons why:**

| As this is a randomised clinical trial, we have provided link to the ClinicalTrials.gov page  where data have been shared. All other items are not applicable |
| --- |

- 1. **Antibodies** – Not applicable

| **Name** | **Citation** | **Supplier** | **Cat no.** | **Clone no.** |
| --- | --- | --- | --- | --- |
|  |  |  |  |  |

- 1. **Cell lines** – Not applicable

| **Name** | **Citation** | **Supplier** | **Cat no.** | **Passage no.** | **Authentication test method** |
| --- | --- | --- | --- | --- | --- |
|  |  |  |  |  |  |

- 1. **Organisms** – Not applicable

| **Name** | **Citation** | **Supplier** | **Strain** | **Sex** | **Age** | **Overall n number** |
| --- | --- | --- | --- | --- | --- | --- |
|  |  |  |  |  |  |  |

- 1. **Sequence based reagents** – Not applicable

| **Name** | **Sequence** | **Supplier** |
| --- | --- | --- |
|  |  |  |

- 1. **Biological samples** – Not applicable

| **Description** | **Source** | **Identifier** |
| --- | --- | --- |
|  |  |  |

- 1. **Deposited data**

| **Name of repository** | **Identifier** | **Link** |
| --- | --- | --- |
| ClinicalTrials.gov | NCT02516605 | <https://clinicaltrials.gov/ct2/show/NCT02516605> |

- 1. **Software** – Not applicable

| **Software name** | **Manufacturer** | **Version** |
| --- | --- | --- |
|  |  |  |

- 1. **Other (*e.g*. drugs, proteins, vectors etc.)** – Not applicable

|  |  |  |
| --- | --- | --- |
|  |  |  |

- 1. **Please provide the details of the corresponding methods author for the manuscript:**

| Not applicable |
| --- |

**2.0 Please confirm for randomised controlled trials all versions of the clinical protocol are included in the submission. These will be published online as supplementary information.**

| For the first submission, redacted versions of the final protocol amendment and the SAP  have been included as supplementary information. All versions of the protocol and SAP  will be included with the revised submission. |
| --- |
